# Inherent instability of simple DNA repeats shapes an evolutionarily stable distribution of repeat lengths

**DOI:** 10.1101/2025.01.09.631797

**Published:** 2025-01-10

**Authors:** Ryan J. McGinty, Daniel J. Balick, Sergei M. Mirkin, Shamil R. Sunyaev

**Affiliations:** 1 Department of Biomedical Informatics, Harvard Medical School, Boston, MA, USA; 2 Department of Biology, Tufts University, Medford, MA, USA

## Abstract

Using the Telomere-to-Telomere reference, we assembled the distribution of simple repeat lengths present in the human genome. Analyzing over two hundred mammalian genomes, we found remarkable consistency in the shape of the distribution across evolutionary epochs. All observed genomes harbor an excess of long repeats, which are prone to developing into repeat expansion disorders. We measured mutation rates for repeat length instability, quantitatively modeled the per-generation action of mutations, and observed the corresponding long-term behavior shaping the repeat length distribution. We found that short repetitive sequences appear to be a straightforward consequence of random substitution. Evolving largely independently, longer repeats (10+ nucleotides) emerge and persist in a rapidly mutating dynamic balance between expansion, contraction and interruption. These mutational processes, collectively, are sufficient to explain the abundance of long repeats, without invoking natural selection. Our analysis constrains properties of molecular mechanisms responsible for maintaining genome fidelity that underlie repeat instability.

## Introduction

Over 2.5% of human genomic DNA consists of simple DNA repeats^1^, Also known as short tandem repeats (STRs) or microsatellites, simple repeats consist of direct tandem repetitions of short sequence motifs, e.g., mononucleotides, dinucleotides, trinucleotides and so forth. In a randomized DNA sequence, the probability of encountering a simple repeat decreases as an exponential function of its length. Yet this relationship fails to predict the enormous overrepresentation of long simple repeats in genomic sequences, including in humans^2–4^. The origin of this overrepresentation remains to be elucidated.

This overrepresentation is even more striking in light of the existence of repeat expansion disorders (REDs), a growing list of severe human diseases caused by disruption of gene function owing to long simple repeats^5,6^. Decades of study have demonstrated that individual repeat tracts vary in length between and even within individuals^6^, owing to frequent expansion and contraction mutations; the rate of these mutations increases with the length of a repeat, a phenomenon known as repeat length instability^7^. Instability is commonly ascribed to DNA strand slippage during replication and/or DNA repair, although a variety of other molecular mechanisms can also contribute^7^. Instability rates differ between various repeat motifs, being particularly pronounced for motifs that form non-B DNA secondary structures^8^. Importantly, when repeat length exceeds a threshold of approximately 75-90 bp, carriers frequently transmit a substantially longer repeat to the next generation. Known as ‘genetic anticipation’, this effect continues to compound in subsequent generations, which leads to more severe presentation and/or earlier age of onset^6^. Recently-developed techniques such as ExpansionHunter^9^ and long-read sequencing have accelerated the discovery of pathogenic repeats, especially those lurking in introns and other non-coding regions^6^. Repeat expansions occur in various cancers^10–12^ and serve as hotspots for genomic rearrangements^13^.

While numerous studies focus on the instability of disease-length repeats, comparatively less is known about shorter repeats, including the so-called ‘long-normal’ alleles that sit immediately below the disease-length threshold. Carriers of long-normal repeat alleles are healthy, but risk transmitting a disease-length allele due to the higher rate of repeat expansion; additionally, some ‘long-normal’ alleles contain protective interruptions that may be lost^6^. Complementing our understanding of long disease-causing repeats, a recent finding identified an autosomal dominant thyroid disorder linked to a (TTTG)_4_ repeat, with a recurrent deletion to (TTTG)_3_ in affected individuals^14^. Additionally, instability of A_8_ and C_8_ repeats in the coding sequences of mismatch repair genes MSH3 and MSH6, respectively, promotes tumor adaptability via frequent frameshifts and subsequent reversions^15^. The latter examples suggest that relatively short repeats, which comprise a much larger portion of the genome, have at least some medical relevance.

In light of the rapidly-growing list of repeat-associated diseases, it is surprising to find repeats harbored in abundance in the genome. Interest in this discrepancy goes back at least three decades^2^ and has led to speculation that natural selection preserves longer repeat lengths despite the risk of disease^16^. The best-supported examples of functionality are specific to telomeric and centromeric repeats^8,16,17^, though some recent studies have suggested that simple repeats play a role in gene regulation^16^. However, before assuming the overabundance of repeats is evidence of functionality, a more basic explanation should be considered: the excess of repeats in the genome are solely a consequence of mutational processes. Several studies, mostly pre-dating the human genome era, considered this premise, but were limited by the availability of sufficiently long genome sequences, lacked robust direct measurements of repeat instability, and/or considered oversimplified mutational models^4,18–31^. Indeed, all such studies of simple repeats have been limited by long-standing technical challenges to sequencing repetitive regions^32–34^. Technological developments led to the release of the human Telomere-to-telomere genome (T2T-CHM13), which more than doubled the number of mapped simple repeats compared to the previous reference genome GRCH38^1^. This warranted a fresh look at the distribution of repeat lengths and whether mutational processes, in the absence of selection, can explain their abundance.

In this study, we measured genome-wide distributions of repeat lengths across mammals, observing that the distribution, including the prevalence of long repeats, is remarkably stable over evolutionary timescales. We modeled the effects of repeat length instability on evolution of the distribution, finding that the observed repeat length distribution can emerge and be maintained solely due to the interplay between distinct mutational processes. After incorporating empirical estimates and inference of repeat length instability rates, the most parsimonious explanation for the abundance and stability of long repeats does not require invoking selection; rather, extreme mutation rates cause long repeats to emerge as independently evolving elements. We discuss how inherent constraints of DNA replication and repair machineries could lead to persistent repeat length polymorphism.

## Results

### Features of the repeat length distribution and evolutionary stability

**Fig. 1.**
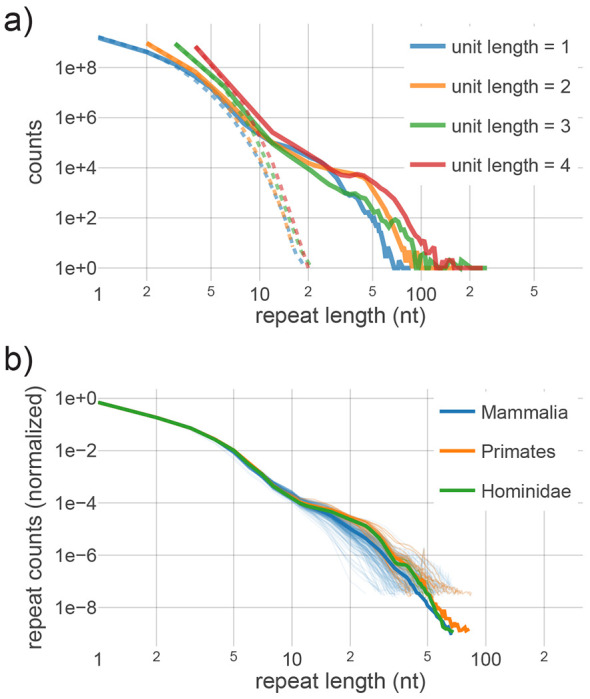
Repeat length distributions by motif length and across phylogenies. **a)** Counts of repeats in human T2T genome pooled by motif unit length (e.g., unit 1 pools distributions for A/T and C/G). Dashed lines represent exponentially-distributed counts in a randomly-shuffled human genome sequence. **b)** Normalized distributions of A_n_ repeats in mammals, primates and hominids. Solid line indicates median values per length bin. Thin transparent lines show individual species within the phylogeny. To appropriately compare assemblies with different genome lengths (after normalization), individual distributions are cut off at the shortest bin containing 30 counts; median calculated without a cutoff. Phylogenies are inclusive (e.g., primates are included as a subset of mammals). Similarity within phylogenies suggests long-term stability of the distribution.

We first assembled a genome-wide distribution of repeat lengths for each simple tandem repeat motif using T2T-CHM13. We naively compared these to distributions from a randomly-shuffled genome sequence, which confirmed an excess of mid- to long-length repeats in the human genome, including those of near-disease length (Fig. 1a). The repeat distribution for a given motif generally consists of at least two qualitatively distinct regimes; short-length repeats (under ~10 nt) appear to be roughly randomly distributed, while mid- to long-length repeats are overrepresented (Fig. 1a). We found this to be the case regardless of motif length or sequence, with the long-length tail displaying some variation between motifs (Fig. 1a, Fig. S1a).

Despite technological differences in sequencing and assembly between T2T-CHM13 and GRCH38, the genome-wide repeat length distributions are nearly identical after normalization (Fig. S1b). We interpret the normalized distribution of repeat lengths, henceforth *P(L)*, as an estimate of the probability of randomly sampling a repeat of length *L* from the distribution of all repeats. Additionally, the estimated *P(L)* from T2T-CHM13 completely overlaps estimates from two individual genomes assembled from moderate coverage short-read sequences (Fig. S1b). Thus, short read sequencing, which inherently limits the length of identifiable repeats, is nonetheless sufficient to reconstruct the well-populated length classes of *P(L)*; long-read sequencing is only required to identify extreme length repeats that occur in low numbers.

We estimated distributions from genome sequences of over 200 mammals from the Zoonomia project, which employed a mixture of short- and long-read sequencing^35^. Each estimated *P(L)* was truncated at the first length with occupancy below 30 in the non-normalized distribution to account for differences in assembly length that affect sampling noise. The normalized distributions appear to be highly conserved in hominids, remain so across primate evolution (with the exception of the loris outgroup), and show surprisingly little change through mammalian evolution despite dramatic changes in genome size (Fig. 1b, Fig. S2). This suggests that both the repeat length distributions and, as a corollary, maintenance of the underlying mechanisms, were largely stable throughout at least 70 million years of primate evolution. The highly conserved evolution of *P(L)* serves as an interesting case in which the empirical observations directly suggest the emergence of a steady state equilibrium.

Given that the distribution is maintained on evolutionary time scales, a prominent extended tail of longer repeats appears to be a generic and conserved feature of *P(L)*. This may be surprising because repeats on the extreme end of this length range have the propensity to develop into repeat-expansion disorders in subsequent generations^5^. One proposed explanation is that longer repeats confer a selective advantage due to some repeat length-specific biological function^16^. As an alternative, we propose that long repeats emerge and are maintained by the complex interplay between distinct mutational forces. Though these hypotheses are not mutually exclusive, we sought to understand the extent to which mutagenesis, alone, is capable of capturing the shape of the distribution, without introducing natural selection.

To avoid ambiguity related to repeat interruptions, all distributions were assembled by counting contiguous repetitions of each motif. This simultaneously defines length transitions due to each mutation type (either nucleotide substitutions or insertions/deletions), which can have one of four effects on a given repeat: lengthening, shortening, splitting one repeat into two (which we term ‘fission’), or joining two repeats into one (which we term ‘fusion’). Deletions and insertions of one or more entire repeat motif units are referred to as contractions and expansions, respectively, in contrast to partial deletions and non-motif insertions. The treatment of interrupted repeats as multiple distinct repeats is supported by previous studies, which have found that length-dependence of mutation rates associated with instability depends on the longest contiguous tract within an interrupted repetitive sequence^36–42^.

### Temporal evolution of the repeat length distribution

To understand the evolution of the repeat length distribution towards a steady state, we built a computational model that incorporates length-changing effects of substitutions, expansions, contractions, and non-motif insertions to follow the equilibration of *P(L)*. To reduce the computational time required to evolve a whole genome sequence and simultaneously track contiguous repeat lengths, we directly manipulated the repeat length distribution, treating the aggregate effects of mutation as deterministic. This deterministic assumption approximates the expectation of *P(L)* under the repeated application of mutations over many generations, ignoring stochasticity in the mutational process and due to factors like genetic drift. The elementary step of the process is deceptively complex, owing to a combination of non-linear transitions in length (i.e., fusion depends on sampling the length distribution twice in each generation) and changes in the total number of repeats (e.g., fission splits one repeat into two). The computational model uses explicit length-dependent rates for each mutational process as inputs, which, ideally, would be fully defined by de novo rate estimates from parent-child trios. However, existing estimates of this relationship^43–49^ lack granular information about each distinct mutagenic process, are available only for particular motifs, and/or are insufficient to estimate rates spanning the full range of observed repeat lengths. Instead, we used de novo data to motivate a parameterization (with minimal degrees of freedom) that characterizes a rapid increase in mutation rates with length, the hallmark of repeat instability. We search for parameters which show a close resemblance between computationally-propagated and empirical distributions.

**Fig. 2.**
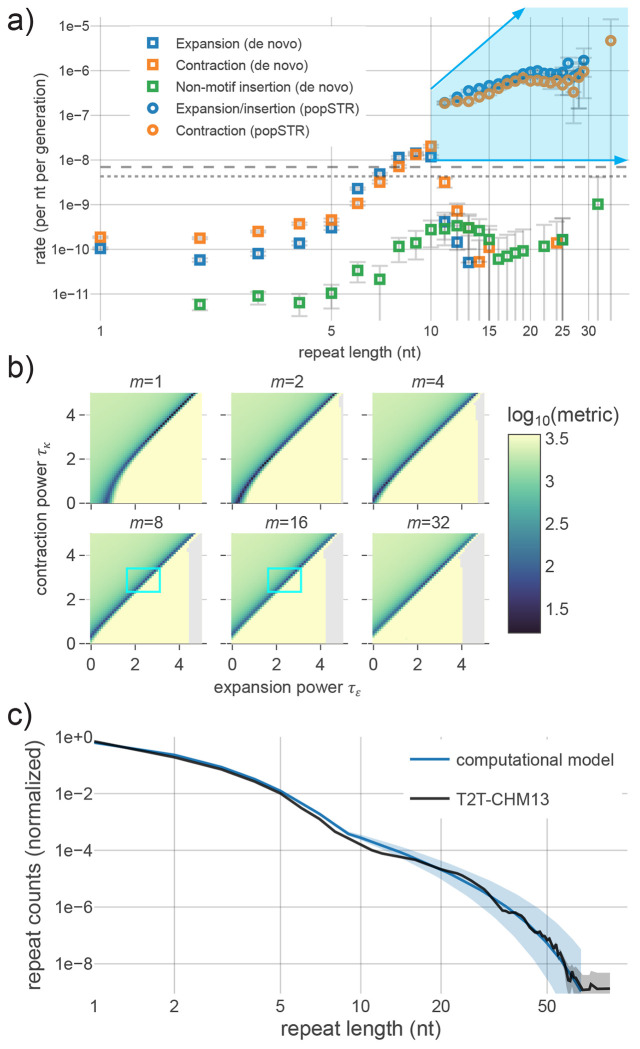
Estimated instability rates, paramerization, and consistency with maintenance of the repeat length distribution. **a)** Separate rate estimates from de novo and popSTR datasets for expansion, contraction and non-motif insertions for A_n_ repeats. Statistical error bars show 95% confidence intervals. Gray dashed and dotted lines show average substitution rates for A>B and B>A (B=C,G or T), respectively. Light blue box illustrates the approximate parameter space explored by computational model; arrows illustrate power law functions (rate ∝ *L*^τ^). **b)** Metric values comparing computationally-propagated and empirical distributions. Computational model runs across a range of parameters: multiplier *m*=[2^0^,2^5^], expansion and contraction power law exponents τ_ε_,τ_κ_=[0,5]. Plane of τ_ε_,τ_κ_ is shown for slices of constant *m*. Color specifies log_10_ of metric values. Gray masks parameter combinations exceeding the linear mutation bound. All runs were initialized using the equilibrium distribution under substitutions alone and were propagated until reaching an approximate steady-state, if applicable. Blue boxes show 99.9% confidence intervals estimated from popSTR data via linear regression. **c)** Comparison of normalized empirical distribution and computational model of best-fit parameters (*m*=2, τ_ε_=1.5, τ_κ_=1.8). Blue transparency shows 95% confidence intervals generated by statistical errors around estimated de novo mutation rates. Gray shading indicates 95% bootstrap confidence intervals on empirical distribution.

To arrive at a set of realistic parameters for repeat mutagenesis, we first estimated the rates of each distinct mutagenic process using de novo substitutions and indels from available short-read trio sequencing datasets (n=9,387 trios) (see Methods). The resulting estimates of expansion, contraction and non-motif insertion rates show a rapid increase for lengths between 5-10 nt (Fig. 2a, Fig. S3) but unexpectedly decrease after ~10nt, inconsistent with our conceptual understanding of repeat length instability. To resolve this, we looked to a recent study from the deCODE group that analyzed instability at mid- to long-length repeats in short-read trio data (n=6,084) using a population structure-aware repeat-length caller named popSTR^49^. We reanalyzed this data to estimate the length-dependency of insertion and deletion rates (here, we specify ‘insertion’ because popSTR cannot distinguish between expansions and non-motif insertions). Consistent with our understanding of repeat length instability, these rates monotonically increase with length until the estimate becomes noisy (roughly *L* >20-30 nt, depending on the motif) (Fig. 2a, Fig. S3). Based on direct contradiction between these two independent estimates, we believe that our de novo estimates display a technical artifact that results from the reduced ability to accurately discriminate length changes within repetitive sequences longer than 9 nt. However, publicly available popSTR calls omit measurements for *L*≤10 nt, extend only to *L*<30 nt, and, again, cannot differentiate expansions from non-motif insertions. Due to this conflation and unknown differences in the error profiles between estimates, we avoided directly merging the rate estimates for subsequent analyses. We further stratified each estimate by the number of units inserted or deleted per mutation (e.g., *L*>*L*+1, *L*>*L*+2, *L*>*L*+3, etc.) and observed that +1/−1 unit changes comprise the vast majority of indels, regardless of repeat length, in both de novo and popSTR data (Fig. S4). Because rate estimates for A_n_/T_n_ mononucleotide repeats are the most robust, we focused on this motif in depth. De novo estimates for repeats up to *L*=8 nt (i.e., where they are reliable) were directly incorporated into our computational model. To describe expansion and contraction rates for longer repeats, we introduced three free parameters (Fig. 2a): *m* describes the rate increase from *L*=8 to 9 (multiplying the rate at *L*=8 to generate the *L*=9 point), a second parameter, *τ_ϵ_*, determines the exponent of a power-law(i.e., the exponent *τ* in the dependence *L^τ^*) representing an instability-driven increase in the per-nucleotide expansion rate beginning at *L*=9, and the third parameter, *τ_K_*, describes an analogous exponent for the contraction rate. Given that our empirical estimates lose accuracy within a rapidly-changing length range, *m* can capture a possible continued extreme increase in the rates, oversimplified as a discrete jump at L=9, prior to a transition to power-law like behavior. To limit the number of degrees of freedom, we assumed that the length dependence of non-motif insertions is dictated by *τ_ϵ_*, the expansion rate exponent, due to their parallel increase in de novo rates (Fig. 2a) and because they likely arise from the same biological mechanism (e.g., synthesis of the inserted nucleotides by an error-prone polymerase). Power-law length dependencies were chosen as a minimal parameter family of curves that includes the possibility of a constant per-nucleotide rate (i.e., *τ*=0, analogous to the constant per-nucleotide substitution rates) and linearity (i.e., *τ*=1), a natural conceptual model for length dependence associated with repeat instability. This parametrization describes the large-length behavior on a scale consistent with the popSTR estimates, which superficially resembles this class of curves (Fig. 2a), but is not intended to represent a specific biological model; the true rate curves are likely more complex due to multiple contributing mechanisms.

### Consistency between computational model results and empirical data

We analyzed the temporal evolution of the repeat length distribution over a wide range of parameter values to determine if any of the late-time distributions produced by the computational model are consistent with the empirical distribution. After 10^9^ generations (sufficient time for substitution-based effects to equilibrate), we measured the distance between the computational and empirical *P(L)* using a goodness-of-fit metric explicitly sensitive to the distribution tail (Methods, Equation 1). Figure 2b displays metric values for a grid of parameters spanning *m*=1–32, *τ_ϵ_*=0–5, and *τ_κ_*=0–5. The parameter combination with lowest metric value (*m*=2, *τ_ϵ_*=1.7, *τ_κ_*=2.0) results in a late-time *P(L)* that closely resembles the observed distribution (Fig. 2b). A range of parameter combinations statistically consistent with the minimum appears along lines just above the diagonal defined by *τ_ϵ_*=*τ_κ_* for each multiplier (*τ_κ_*-*τ_ϵ_*≈0.3-0.4 with a weak *m* dependence; see Methods for description of statistical procedure). Inconsistent parameters broadly separate into two qualitative categories: *τ_κ_*- *τ_ϵ_*≳0.5 yields distributions that underestimate the long repeat tail, while *τ_κ_*- *τ_ϵ_*≲0.2 yields distributions vastly overestimating the long tail, most of which are subject to explosive growth in all length bins (Figs. S5, S6a). Variation within these two classes is largely characterized by the value of *τ_ϵ_-τ_κ_* (Fig. S5). Notably, a number of parameter combinations consistent with the best fitting parameters resemble the popSTR-based rate estimates (*m*~8-16, *τ*_*ϵ*_ and *τ_κ_*~1.5-3.5) (Fig. 2d, S6b). These popSTR-like parameters become consistent with the best-fitting combination within ~1-5x10^7^ generations (Fig. S6c), suggesting equilibration can occur within primate-divergence timescales, even from a highly diverged state. Collectively, these results show the plausibility that the observed repeat length distribution, including the excess of mid-to-long-length repeats, may simply be a result of the interplay between distinct mutational processes, rather than a consequence of selection.

### Maintenance of the repeat length distribution in steady state

To understand the complex interplay between mutational processes that shapes and stabilizes the distribution of repeat lengths, we constructed an analytic model of the dynamics. This analytic approximation captures the behavior of *P(L)* after the mutational process reaches steady state (see Methods and Supplementary Note SN1). A number of previous studies have constructed mathematical models of repeat instability to study repeat length evolution^19,22,25–29,31^, including a notable study by Lai and Sun^30^ that incorporates many of the elements detailed herein. However, the combination of empirical rate estimates, a robust genome assembly, and our phylogenetic observations motivated the construction of a model from first principles that is directly informed by this collection of observations. In particular, our analytic construction was influenced by the observation of pervasive expansion-contraction bias at most repeat lengths (Figs. 2a, S3), the incorporation of non-motif insertions, and the primarily single unit changes in length that result from repeat instability in each generation (Fig S4).

We first constructed a discrete equation for the change in the number of repeats at a given length in a single generation due to the deterministic action of mutations (i.e., in the absence of selection and stochasticity in the mutational process, consistent with our computational model). We then imposed a steady state condition by requiring that the sum of all changes in and out of each length class vanishes at each time step after equilibration. Despite the simplifying assumption of steady state, the full dynamical equation cannot be solved generically. However, our estimates of de novo mutation rates suggested a dichotomy exists in the primary driver of changes in length between short and longer repeats (i.e., primarily substitutions for L<8 A-mononucleotide repeats vs. expansions and contractions for L>10). Accordingly, short and long repeat dynamics can be treated as separable (i.e., under the approximation of a separation of repeat length scales), leading to simpler approximations of both length regimes. Transitions between the short and long repeat regimes, while present, remain negligible in all realistic scenarios (see SN1).

**Fig. 3.**
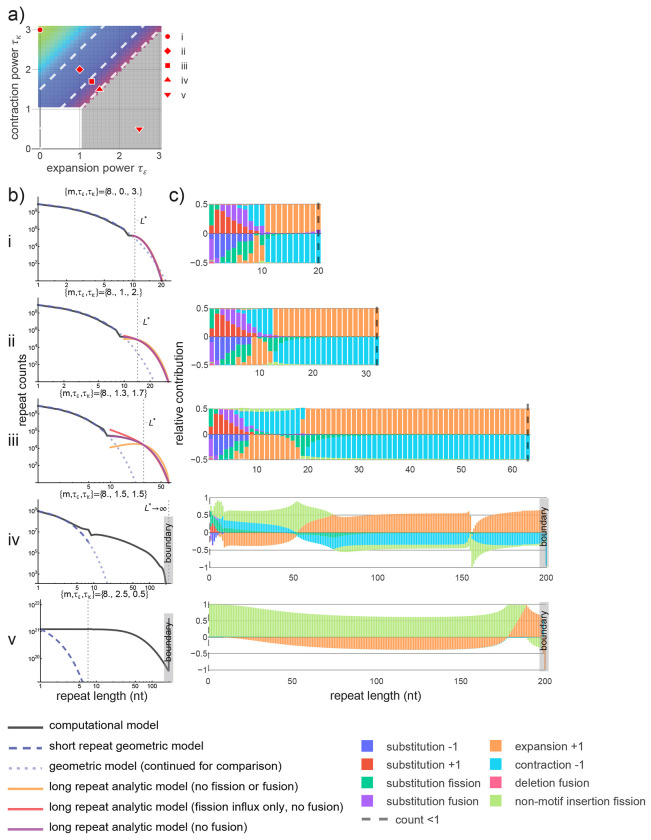
Dynamical regimes distinguishable by dominant mutational effects. **a)** Five parameter combinations with τ_ε_+τ_κ_=3 in distinct dynamical regimes, corresponding to plots in (b) and (c). Dotted lines divide the parameter space into approximate dynamical regimes. **b)** Comparisons between computational model results, analytic approximations and numerical solutions of approximate steady state equations (see Methods, Supplementary Note SN1). Short length regime at equilibrium is geometrically distributed (blue dashed lines). For long repeats, numerical solutions are shown for three nested approximations to the steady state equation in the continuum limit (*L*≫1) in the absence of fusion (due to neglibile rates). Local transitions (*L* to *L*+/−1) were modeled as a combination of symmetric (diffusive) and asymmetric (directional) components. Under strong contraction-bias (b:i), all three approximations remain valid indicating that the dynamics are well approximated by neglecting fission entirely (orange). Under moderate contraction bias (b:ii), outflux due to fission becomes non-negligible (red) at intermediate lengths (e.g., *L*~11-12). Both influx and outflux due to fission are required (purple) for low contraction bias (b:iii), including parameter combinations consistent with the empirical distribution. Plots (b:iv-v) display non-equilibrium dynamics leading to rapid increase in repeat counts (true distribution extends indefinitely above the boundary imposed at *L*=200 for computational feasibility) and explosive growth in genome size. Steady-state analytics do not apply. **c)** Computational model plots of relative contributions to net flux (in minus out) per length bin for different mutational transitions. Consistent with analytic predictions, fission is subdominant under strong contraction bias, has relevant outflux under moderate to weak contraction bias, and relavant influx at intermediate lengths under weak contraction bias. In equilibrium, the distribution is stabilized in detailed balance (net influx = outflux). Nonequilibrium dynamics (c:iv-v) are generated by fluxes that do not sum to zero, leading to indefinite genome growth; distribution extends to length boundary of computational model.

For short repeats, we treated indel mutations as negligible and showed that a geometric distribution (see Methods, Equation 2) exactly solves the steady state equation under two-way substitutions alone (see SN1). For longer repeats, we constructed a partial differential equation (PDE) that approximates the discrete equation and studied its properties in steady state. To facilitate future use cases (e.g., updated empirical estimates or the study of other organisms), the relevant equations are provided in Supplementary Note SN1 for generic length-dependent rates of expansion, contraction, and non-motif insertion. For direct comparison, we imposed the same parameterization of instability rates used in our computational model. To find the steady-state distributions, we simplified the PDE by treating fusion as negligible to long repeat dynamics (Methods Equations 3 and 6, SN1) and produced numerical solutions to the time-independent ordinary differential equation (ODE). For parameters that approach steady state, the late time behavior of the computational modeled distribution is accurately described for short and long repeats, respectively, by the geometric distribution and numerical solutions to the ODE without fusion. We further approximated the ODE (Methods Equations 4 and 5, SN1) to isolate the dominant effects within specific parameter regimes (Fig. 3a–b, SN1). Using our computational results, we decomposed the per-generation fluxes in and out of each length class into relative contributions from each mutational type to identify dominant mutational processes maintaining steady state (Fig. 3c, SN1); the accuracy of each approximation to the PDE was confirmed by analyzing the net magnitudes of fission and fusion within each length class and regime (Supplementary Note Figs. SN1, SN4–7).

We used this model to study the shape and stability of the empirical distribution and distinctions between repeats in different length regimes under mutational forces alone. Expansions and contractions remain non-negligible for any long repeat across the space of parameters that lead to stable late-time distributions, highlighting the importance of repeat length instability to the maintenance of long repeats. For extreme parameters that stabilize (i.e., *τ_κ_*≫*τ_ϵ_*), the dynamics of all long repeats are dominated by expansion and contraction, alone, leading to a distribution that truncates more rapidly than under substitutions alone (i.e., a *depletion* of long repeats relative to a geometric distribution). In contrast, for parameters consistent with the empirical A-mononucleotide distribution, an intermediate length regime emerges, characterized by the relevance of repeat fission. An accurate description of the shape of the distribution requires fission to account for the loss of repeats from the extreme tail (i.e., the longest populated length bins) and gain of intermediate length repeats. The relative contributions of fission due to substitutions and non-motif insertions are parameter-dependent; within the range of popSTR-consistent parameters, substitution is the primary driver of fission up to lengths of roughly 20 nt, while longer repeats are primarily interrupted by non-motif insertions (see SN1). Fission-based losses in the extreme tail are insufficient to fully counteract length increases due to expansion, independent of the mutational mechanism and parameter values. Instead, contraction is primarily responsible for truncating the distribution at finite repeat length but can be bolstered by both substitution- and non-motif insertion-based fission. The dynamics of the long repeat regime decouples from that of short repeats such that rapidly mutating long repeats effectively become independently evolving genomic elements, categorically distinct from random sequences of the same length. The abundance of long repeats in the genome may therefore be a consequence of their largely unencumbered evolution caused by rapid changes in length.

### Application to longer motif lengths

Increasing motif length results in successively fewer observed repeats, resulting in statistical noise that precluded direct use of our computational inference procedure. However, extending our analytic understanding beyond mononucleotides is straightforward. For all longer motifs, we observed that expansions and contractions result in predominantly single unit changes in length (Fig. S4), suggesting the mechanics of repeat length instability are analogous to mononucleotides. Our estimates show that a sharp increase in instability rates with length is a consistent feature of all motifs (Fig. S3), which again suggests separability of length scales into substitution-dominated and instability-dominated dynamical regimes. However, the effective rates of substitutions differ by motif length: a single substitution is sufficient to disrupt any motif, while increasing repeat length for extended motifs can require multiple substitutions (up to the unit length), depending on the sequence context of flanking regions We estimated substitution rates for each motif to account for context dependence and incorporated this into a computational model of substitutions alone (Methods). This confirmed that the reduction in length-increasing substitutions results in a more rapid decay of the geometric distribution for short repeats, relative to mononucleotides (Fig. S7). Assuming the same instability rates, this would result in a more immediate transition to the instability-dominated regime (counting in number of repeated units *L*); this is consistent with empirical results for longer unit lengths (Fig. 1a, S1a). Interestingly, by plotting the distributions (Figs. 1a, S1a) and rate curves (Fig. S3) in total nucleotide length (*L* x unit length), we found that the transition between the substitution- and instability-dominated regimes occurs in roughly the same range (8-12 nt) for unit lengths of 1-4 nt.

## Discussion

Motivated to understand the origin, prevalence and maintenance of simple tandem repeats in the genome, we constructed a model of repeat evolution under mutagenesis alone that bridges short- and long-timescale observations of repeat length instability. We demonstrate that mutation alone is capable of explaining the shape of the genome-wide distribution; furthermore, the observable transition to repeat length instability at some intermediate length, along with an apparent initial expansion bias, is largely responsible for the abundance of long repeats in the genome. This observation does not preclude selection at specific loci, whether beneficial or disease-associated, provided these comprise a small portion of repeats in the genome.

Length-dependent expansion-contraction bias is evident in our de novo estimates; incorporating associated degrees of freedom into the mutational process is sufficient to truncate the distribution at finite lengths due to substantial contraction-bias. This implicitly prevents the growth of repeats to disease-relevant lengths, suggesting natural selection as a disease-prevention mechanism may not be essential. Previous literature suggested that interruptions prevent disease by providing a stopping force that counteracts indefinite expansion^36,42,48,50–52^, thereby truncating the distribution; based on our analyses, the rate of interruptions is far too low and must be augmented by contraction. However, truncation of the distribution occurs at lengths inaccessible in our rate estimates, requiring parameterization. If selection, rather than contraction bias, is responsible for terminating the distribution below disease length, it would have to be enormously efficient to counteract instability-driven expansion rates and act globally across all sufficiently long repeats. Further disentangling the roles of mutation and selection will require a concrete model of both processes simultaneously and an accurate measurement of de novo rates across all lengths present in the genomic distribution (which remains a technical challenge).

Analysis of the steady state dynamics led to the qualitative insight that the dynamics of short and long repeats decouple due to the rapid onset, and subsequent dominance, of repeat length instability. Short repeat dynamics are dictated by substitutions alone such that repeats within this regime are roughly indistinguishable from random strings of nucleotides of the same length. The long length tail of the distribution is produced and maintained in a distinct dynamic balance between expansion, contraction, and fission. Long repeats are primarily subject to distinct mutational forces, exhibiting rapid changes in length and a higher rate of repeat fission, which increases their total number; mid-length repeats primarily experience fission due to substitution, while the longest repeats are primarily interrupted by non-motif insertions. In this sense, long repeats emerge as independently evolving genomic elements (with parallels to the concept of selfish genetic elements^53–55^), in stark contrast to short repetitive sequences. Repeat length instability dominates above roughly 10 nt, leading to a natural boundary for the shortest ‘unstable’ repeat. However, the underlying mutational mechanisms may be better informed by measuring the lowest length where expansion or contraction rates start exceeding the background indel rate (as low as two units for many motifs; Figs. 2, S3). The corresponding scientific goals associated with these distinctions (i.e., identifying mechanistic onset vs. differentiating independently evolving elements) are indeed central to a debate concerning the definition of unstable repeats^56^.

Provided selection plays little role in directly modifying repeat length, the conservation of the distribution in steady state implies that the underlying mutational mechanisms (i.e., DNA replication and repair) are highly conserved. Generically, such mechanisms play a broad role in maintaining sequence fidelity of the entire genome, primarily preventing single nucleotide mutations; due to the substantially larger target size, it is unlikely that machinery responsible for both single site mutations and instability-driven length changes are optimized to properties of the latter. The abundance of long repeats may thus be an inescapable consequence of the pleiotropic function of the machinery maintaining genome-wide sequence fidelity.

It remains unclear which biological mechanisms control key properties of repeat length instability. The proposed mechanism(s) should be able to explain length dependencies of instability rates (Fig. 2a, S3) that show: a) rapid onset from ~6-10nt, surpassing the rate of substitutions, b) greater-than-linear increase in the expansion/contraction rate per target above 10nt, c) generically asymmetric rates of expansion and contraction with initial expansion-bias and terminal contraction-bias, and d) primarily single-unit changes in length (Fig. S4). Surprisingly, these observations appear to be largely independent of both motif sequence and unit length (Fig. S3), suggesting a common biological origin.

Two widely studied mechanisms, replication slippage and mismatch repair (MMR), likely explains part of the story^7,8,57–61^. Slippage, when newly synthesized DNA partially unwinds and realigns out of register, should strongly depend on the unit length; however, we see only minor variation associated with unit length (Fig. S3). While slippage during DNA replication produces loop-outs on both strands symmetrically^60^, subsequent small loop-processing by MMR preferentially results in contractions^62^ due to bias towards the nascent strand^63^. Slipped-strand structures may be a motif-independent source of loop-outs subject to the same MMR-processing; in contrast, other secondary structures are motif-specific and therefore cannot be the primary source of repeat instability but can potentially explain differences between motifs^64^ (Fig. S1a). Importantly, the observation of mostly single-unit expansions/contractions rules out mechanisms involving larger structures (e.g., long hairpins that cannot be processed by MutS*β*^65–70^), as these would be expected to generate multi-unit indels.

Single-unit expansions have also been observed in a different context: Okazaki fragment processing by flap-endonuclease FEN1^71^. Incomplete flap removal of the displaced lagging-strand primer may lead to expansion bias^72^ and introduce an associated length scale. A secondary mechanism takes over when flaps exceed 30 nt^73^; speculatively, long repeats could give rise to long flaps. This illustrates how different mechanistic explanations may apply to repeats of distinct lengths, generating emergent properties like length-dependent expansion-contraction bias. Likewise, another nuclease, FAN1, is a genetic modifier of several repeat expansion disorders, may be involved in processing slip-outs resulting from replication and transcription, and has differential activity depending on flap length^74^.

In sum, our data implies that a simple mutational process accounts for the abundance of mid-length tandem DNA repeats regardless of their sequence composition, which in turn, predisposes the genome for further instability. Subsequent large-scale expansions leading to genetic diseases are attributed to a subset of these repeats that can form stable secondary structures (a.k.a. structure-prone DNA repeats), and they are governed by separate mechanisms discussed elsewhere^7^. Incorporation of a broad range of recent data revealed new insights about long-held tenets of repeat length instability, yet many open questions remain. Inclusion of more comprehensive estimates, experimentation and new analytic approaches may help elucidate the complex biology of instability and evolutionary stability of repetitive sequences in our genomes.

## Methods

### Genome sources

Genome fasta files for T2T-CHM13_2.0 were downloaded from UCSC: http://hgdownload.soe.ucsc.edu/downloads.html#human. Alternate human assemblies and mammalian genomes were downloaded from the NCBI genome database: https://www.ncbi.nlm.nih.gov/datasets/genome/

### Generation of empirical repeat length distributions

Repeat length distributions were generated by counting consecutive complete motifs (ie. perfect motifs, no interruptions and no partial motifs). For each motif, repeats were located by converting the sequence of each chromosome into a binary array of matching and non-matching positions via ‘regex’ parsing, which then allowed for the generation of a histogram according to the length of consecutive motifs (or consecutive non-motifs). For motifs of unit length *n*>1 nt, the genome was split into *n* distinct frames, and each frame was used to generate a histogram for each motif. All histograms for equivalent motifs (eg. A=A/T, AC=AC/CA/TG/GT) were then summed. Finally, each histogram was then normalized by the sum of the counts in all length bins to generate a probability distribution *P(L)*.

Bootstrap confidence intervals were generated around the T2T-CHM13 repeat length distribution. The genome was divided into 1Mb contiguous non-overlapping segments, discarding any sub-1Mb chromosome ends. Repeat length distributions were measured for each segment. A distribution for the full-length genome was then reconstituted by randomly sampling from these segments, allowing replacements, and summing the distributions from each segment. This process was repeated 1000 times, after which 95% confidence intervals were generated by separately taking the minimum and maximum in each length bin by removing the top and bottom 25 counts.

For the various mammalian genomes, the same counting procedure was applied. Assemblies generated from short-read sequencing frequently contain many short contigs which typically originate from poorly-sequenced regions containing transposable elements; any contig of length <10 kb was discarded. Taxonomic data was retrieved from https://ftp.ncbi.nlm.nih.gov/pub/taxonomy/. The median distribution of a given taxonomic group was assembled by gathering the *P(L)* distributions for every member of the group (i.e., for primates this includes humans, and for mammals this includes primates), and taking the value of the median species for each length bin.

### Bioinformatic estimation of substitution and indel rates

De novo mutation datasets, representing a total of 10,912 parent-child trios, were acquired as VCF files (or equivalent) from various published sources^75–82^. We assumed that all individuals have the same underlying mutation rates. De novo data was mapped to GRCh38 either in the original study, or subsequently, using pyliftover (pypi.org/project/pyliftover/). Because of the limitations of the VCF file format (e.g., no read depth information on unmutated positions), 100kb regions lacking any substitutions across the combined dataset were assumed to suffer from mappability issues and masked when estimating rates. The average substitution rate was estimated to be 1.2x10^−8^, calculated as: number of substitutions / approximate number of sequenceable nucleotides in the diploid genome / number of offspring genomes in the dataset. We classified substitutions according to six categories based on trinucleotide context and the motif in question, as follows: for the example of A_n_ motifs, using B to represent non-A nucleotides, we determined rates (in parentheses) of ABB>AAB and BBA>BAA (4.77x10^−9^) representing repeat-lengthening events, AAB>ABB and BAA>BBA (8.08x10^−9^) representing repeat-shortening events, ABA>AAA (2.87x10^−9^) representing fusion events, AAA>ABA (4.54x10^−9^) representing fissions, BBB>BAB (3.97x10^−9^) representing the rate of A_1_ creation, and BAB>BBB (6.45x10^−9^) representing the loss of A_1_. Rates of substitutions of B which do not create an A were not estimated.

We calculated indel rates as a function of repeat length. Using positional information, upstream and downstream sequences for each event were pulled from the reference genome, under the assumption that the sequence of the parental genome is identical to the reference genome. For every focal motif, we used the reference sequence to determine repeat length. Indel rates per repeat length per motif were estimated by dividing by the number of repeats of that length in the masked GRCh38 genome (see above). Each indel was classified as an expansion, contraction or non-motif insertion, additionally measuring how many motifs were added/removed in the event. We limited mutations in our computational model to +1/−1 unit changes in length at appropriate rates. We also measured the rate of indels for all B positions (with respect to each motif; A_n_ rates in parentheses), separately estimating the rates of BB>BBB (1.44x10^−10^), BBB>BB (4.56x10^−12^) and ABA>AA (2.89x10^−10^) events. Because B strings were not modeled as having length-dependent instability, we measured the average rate, i.e., the rate per unit.

The popSTR repeat instability dataset, representing 6,084 parent-child trios, was acquired from the supplement of ^49^ Files ‘bpinvolved_extended’ and ‘mutRateDataAll.gz’ were downloaded from https://github.com/DecodeGenetics/mDNM_analysisAndData. Due to our focus on uninterrupted repeats, we measured the longest contiguous repeat within the given coordinates for each event. We limited the dataset to loci where the popSTR-reported reference repeat length agreed without our own measurement in GRCh38. The popSTR dataset contains a mix of phased and unphased data; where the parental length for a given mutation was not assigned by phasing, we assumed that it originated from the parental copy which minimizes the difference in length between the proband repeat and any of the parental repeats. Skipping this phasing step and assuming that all events originated from the reference length allele (but retaining the size and direction of the event), as we do for the de novo dataset, results in only minor differences in counts per length bin. The ‘mutRateDataAll.gz’ file contains information on the number of trios where all three samples passed sequencing quality filters at a given locus, and the length of the repeat at each locus in GRCh38 but lacks information on the parental genotypes for each of these loci. For the denominator of the popSTR mutation rates, we thus generated a distribution of counts using the reference length for each locus multiplied by twice the number of passing trios (for two parental alleles).

For de novo substitution and indel rates, as well as popSTR rates, we calculated confidence intervals based on 200 Poisson samples of the mutation counts, removing the top 5 and bottom 5 values per length bin (see Fig. 2a, Fig. S3). We determined lines of best-fit (corresponding to empirical estimates of parameters *τ_ϵ_* and *τ_κ_*; see below) using the SciPy linregress package (https://docs.scipy.org/doc/scipy/reference/generated/scipy.stats.linregress.html) to perform linear regression in log-log space for popSTR estimates corresponding to A_12-19_ (i.e., the portion of the rate estimates resembling a power law and not subject to large uncertainty). The 99.9% confidence intervals for the slopes (i.e., power law exponents) were used to determine a range of intercepts at L=9 and the corresponding multiplier *m* (see below).

### Computational modeling of repeat length dynamics

We used a custom-written script in Python 3 that models repeat dynamics by directly manipulating the distribution of repeat lengths. We simultaneously tracked and manipulated the length distribution of B strings. As detailed above, we assumed a binary genome consisting only of A and B sites, where A is a repeat unit and B represents any non-A unit; as a result, B strings do not *a priori* represent repetitive sequences. Mutations are applied in aggregate such that, in each generation, repeats transition between integer length bins according to rules associated with each mutational process, while the B distribution is updated accordingly (e.g., a substitution that lengthens a repeat simultaneously shortens a B string). Mutation rates were restricted to be sufficiently low to model only a single mutation event per repeat per generation. The non-normalized distribution was evolved and subsequently normalized to create a probability distribution for comparison to empirical data. This approach is far more computationally efficient than simulating an entire genomic sequence, subsequently applying mutations and generating a distribution; computational time in our script scales with the number of length bins rather than with the length of the genome. Tracking only the distribution discards information about the location of particular mutations, instead generating an expected number of mutations for each category per length bin per generation. Except where specified, we used a deterministic approximation to assess the behavior of the expectation value of each bin as the distribution evolves toward steady state via repeated application of the mutation kernel. To understand the impact of stochastic fluctuations on the steady state distribution, we additionally implemented a model that represents fluctuations by Poisson sampling the expected change to each length bin per generation. We model stochastic fluctuations around the applicable rates by sampling mutational counts, but without constraining individual transitions (i.e., a net number of mutations may leave a given class, but the number introduced elsewhere, as a result, is appropriately distributed only on average due to an independent sampling procedure). All subsequent analyses were performed using the deterministic results, as modeling independent fluctuations in each bin showed no qualitative differences (Fig. S8a–b).

Mutations affect the distribution via the following well-defined rules for substitutions and indels. These rules assume that each mutation adds, subtracts or substitutes a complete repeat unit. Using the example of a repeat of *L*=5, a lengthening substitution subtracts one count from the *L*=5 bin and adds one to the *L*=6 bin. A shortening substitution subtracts one from the *L*=5 bin and adds one to the *L*=4 bin. A substitution causing repeat fission subtracts one from the *L*=5 bin and adds two new repeats, either one *L*=1 and one *L*=3, or two *L*=2 (when evolving the distribution as a whole, both occur simultaneously with appropriate relative rates). The reverse process of fission is fusion, in which an L=5 repeat can be generated by fusing an *L*=1 with an *L*=3, or by fusing two *L*=2 repeats, while the mutated B unit is replaced with an A unit and added to the repeat length. Lengthening and shortening substitutions act locally (i.e., counts leave the *L* bin and move to the adjacent *L*+1 and *L*−1 bins, respectively). Substitution of an *L*=1 in the A distribution also corresponds to fusion of B strings; the reverse, i.e., substitution of a length one B string, generates fusion in the A distribution. Fission and fusion substitutions inherently act non-locally: fission results in the loss of one count in the *L* bin and gain of two counts that are evenly distributed across all bins of length <=*L*-2; fusion evenly subtracts two counts from bins <=*L*-2 to add a count to *L*. The net effect of substitutions conserves the total length of the genome, i.e., the sum of the length of all A repeats plus the sum of the length of B strings remains constant under substitutions alone.

The rates of lengthening, shortening, fission and fusion substitutions per generation are separately estimated using the three-unit context: BBA>BAA (or ABB>AAB) for lengthening substitutions, AAB>ABB (or BAA>BBA) for shortening substitutions, AAA>ABA for fissions, and ABA>AAA for fusions. All substitution rates were assumed to be independent of repeat length, based on our previous observations showing little to no rate increase with increasing repeat length^83^. The target size for lengthening substitutions is two per repeat (i.e., the two sites adjacent to each repeat boundary). Likewise, the target size for shortening substitutions is also two per repeat, representing the two boundary units of the repeat (assuming *L*>1). The target size for fission substitutions is *L*−2 per repeat, representing all non-boundary units within the repeat. The target size for all fusion events is proportional to the *L*=1 count of the B distribution. Equations governing these processes are described in detail in Supplementary Note SN1.

Indel mutations operate under an analogous logic, but with a few important distinctions. Indels, by definition, do not conserve the length of the genome. Expansions and contractions act strictly locally, but the location of the event is indistinguishable within the repeat, affecting any of the units rather than just the boundaries; this results in a per-repeat target size *L* for these mutations, rather than 2. Non-motif insertions (i.e., AA>ABA) cause fission, resulting in the loss of one count in the *L* bin and gain of two counts that are evenly distributed across all bins of length ≤*L*−1; deletion of a B string of *L*=1 (i.e., ABA>AA) causes fusion, which evenly subtracts two counts from bins of length <=*L*−1 and adds one count to bin *L*. Indel rates for expansions and contractions are incorporated in a length-dependent manner, described above, in contrast to substitution rates. We did not model length dependence for B indels, as most B strings represent a combination of nucleotides and not necessarily STRs with any biological relevance. This assumption should not impact the evolution of the A distribution, which is only coupled to the *L*=1 class of the B distribution; this length class is dominated by substitution rate dynamics and not subject to repeat instability.

To model evolution of the distribution, we apply this mutational process for a large number of iterations *i*, using the geometric distribution under substitution alone as the initial condition. Low mutation rates associated with low lengths, which are substitution dominated, require excessive computational time to equilibrate (on the order of the inverse of the substitution rate). To reduce computational time, we introduced a time-rescaling factor *r* that multiplies all mutation rates by the same constant 10*^r^* such that each iteration represents 10*^r^* generations of evolution for a total of *T*=10*^ir^* generations (assuming a constant *r* at all time points). To ensure that we remain in the linear mutation regime (i.e., avoid introducing double, triple, etc. mutational events per repeat per generation), *r* was limited to values where the sum of all mutation rates per repeat per generation remains much less than one (with a maximum of 0.1) in all populated length bins. Imposing this bound inherently limits the space of explorable parameters for a given *r*; parameters with higher mutation rates must be run with lower *r*, which takes more computational time. Full exploration of the grid of allowed parameters would require infeasible computational times for low values of *r* (e.g., *r*=1), while large values of r (e.g., *r*=4) exit the linear mutation regime in at least one length class for a greater number of parameter combinations.

To compensate for this, we introduced a progressive time rescaling scheme within each run. Initially, *i*=10^4^ iterations are performed at *r*=5 (for a total of *T*=10^9^ generations) and parameters that do not exceed the total mutation rate bound are collected. For parameters that exceed the mutation rate bound at a given *r*, we set any individual mutation rate at a given length that exceeds 0.1 to 0.1; this results in deviation from a power-law length dependence due to saturation to a constant rate above this length. Using the final distribution from the first stage as the initial distribution, we subsequently repeat this procedure, running *i*=10^4^ iterations with *r*=4, followed by *i*=10^4^ iterations with *r*=3, *i*=10^4^ iterations with *r*=2, *i*=10^5^ iterations with *r*=1, and *i*=10^6^ iterations with *r*=0. This procedure approximates the equilibration for a total of *T*≥10^9^ generations of evolution. While larger values of *r* are poor approximations to a linear mutation regime (i.e., they aggregate mutations over too many generations, temporarily exceeding the linear mutation bound), the output of each step provides an initial distribution for the subsequent step that is progressively closer to equilibrium. As a result, the distributions reach steady state (when applicable) in far fewer total iterations. For the subset of parameters that could feasibly be run for *T*=10^9^ generations without progressive time rescaling, we confirmed that the grid of metric values is nearly identical (Fig. S8c) to those produced using the progressive time rescaling scheme (excluding parameter combinations that do not equilibrate, which are expected to differ). This allowed us to effectively model *T*≥10^9^ total generations of evolution to ensure that substitution-based processes with per-repeat rates on the order of 10^−8^ equilibrate to very near steady state.

We imposed a reflective boundary condition at very large lengths; this is necessary due to computation constraints, as the expansion, contraction, and non-motif insertion rates are length-dependent power laws that rapidly increase to unrealistic values at arbitrarily large lengths. To avoid indefinitely increasing rates, the reflective boundary maps all transitions to lengths above *L_boundary_* to the bin at *L_boundary_*. This introduces an artifact in the distribution associated with the change in transition rules representing mutations, which leads to an observable aggregation at the boundary for any computational run resulting in a distribution with finite counts at or beyond *L_boundary_*. This is primarily to limit computational time spent on unrealistic parameter combinations; this boundary must therefore be imposed at a length substantially longer than the longest repeats considered in the empirical distribution to avoid artifactual boundary effects for plausible parameter combinations. The resulting aggregation at *L_boundary_* also facilitates the identification of unstable parameter combinations that show a marked increase in counts at this boundary. When using progressive time-scaling, a count in excess of 1,000 in the *L*_boundary_ length class triggers the transition to the next speedup stage. *L*_boundary_ is specified as a command line option (default =100 for both A and B distributions).

The script relies on the following as inputs: an initial distribution for A and B (i.e., motif and non-motif) repeat lengths, per-target substitution rates in three-unit context, and per-target mutation rate curves for expansions, contractions and non-motif insertions. Substitution rates and length-dependent indel rate curves are imported from external files (see above for estimated substitution rates, below for generation of parameterized rate curves); these files, along with the initial repeat length distribution table, can be replaced with appropriate tables for other purposes, if desired. This table must specify rates for each mutational process at all lengths intended to be computationally modeled (i.e., from 1 to *L*_boundary_). For normalized length distributions that reach steady state, the initial distribution can be chosen arbitrarily; here, we used a distribution generated from the shuffled human genome sequence (T2T-CHM13), which is geometric in both A and B lengths.

Stochastics can be introduced using a command line option to model fluctuations in the mutational process; the number of mutations in and out of each length bin are separately Poisson sampled (using numpy.random.poisson) around the expected number of mutational counts in each iteration.

After each run, we output a file containing repeat length counts reported after every *ΔT*=10^6^ generations to show the temporal evolution of the distribution. We subsequently normalized the resulting distributions by dividing each length bin by the total number of repeats in the distribution.

The relative contribution of each mutational force was assessed by producing a single-generation plot of the transitions in and out of each length bin at the final time point (i.e., once steady state was reached, if applicable). To produce these plots, we applied the mutation kernel for a single generation and separately computed the number of fission, fusion and local changes for substitutions and indels. For each length bin, the magnitude of total flux in and out was normalized to one. Length bins that have equilibrated should contain equal fluxes in and out; steady state occurs only when all bins show equilibrated fluxes.

### Inference of optimal repeat instability rate curves

Given our observations indicating a stable distribution of repeat lengths over phylogenetic time scales, we sought to identify mutation rates capable of explaining this observation. To test the hypothesis that mutational forces alone can generate and stabilize the observed repeat distribution, we first incorporated the full extent of reliable empirical estimates of the substitution and indel rates described above. As discussed above, substitution rates were assumed to be length independent. We focused on A_n_ repeats, as both the distributions and rate curves were supported by the most empirical data. For expansion, contraction, and non-motif insertion rates for A_n_ repeats, reliable estimates from trio data extended only to repeat lengths of *L*=8, beyond which we parameterized the length dependence. Beginning at *L*=9, each instability rate was parameterized by a multiplier *m* describing a rapid local increase at *L*=9 relative to *L*=8, akin to an initial value of the asymptotic dependence at long lengths; the asymptotic rate curve was parameterized by a single parameter *τ* defining the exponent of a power law (i.e., rate(L) ∝ *m* L*^τ^*) extending from *L*=9 to a maximum computationally modeled length *L_boundary_*. We note that the choice of a power-law dependence was arbitrary and instead could be replaced with any family of monotonic single-parameter curves (e.g., exponential growth). Expansion and contraction rates were defined with the same multiplier *m*, limiting the number of independent parameters, but with separate asymptotic exponents, *τ_ϵ_* and *τ_κ_*, respectively. To further limit the number of inferred parameters, non-motif insertions were fixed to the same asymptotic exponent as expansions. This is consistent with the empirical observation that the rate curves are roughly parallel and the assumption that the same biological mechanisms are responsible for all insertion rates (i.e., expansion and non-motif insertions are both single unit insertions into a repeat). In total, each run required specifying three degrees of freedom (*m*, *τ_ϵ_* and *τ_κ_*) that collectively parameterize the instability rates at lengths beyond our direct estimates.

Using this parameterization, we ran the computational model over a discrete grid of parameter combinations: multipliers *m*={1, 2, 4, 8, 16, 32} and exponents in the range *τ_ϵ_*,*τ_κ_*=[0, 5] spaced in increments of Δ*τ*=0.1. These curves are restricted to monotonically increasing rates, consistent with our biological understanding of repeat instability, but includes a lower limit of *τ*=0 describing no length dependence above L=8 (i.e., a local jump in rates at L=9, with no further rate increase). Mutation rates are definitionally bounded at a maximum value of 1 (i.e., every target mutates in each generation). Excessive monotonic rate increase (i.e., *τ*≫1) would result in unrealistically saturated mutation rates at measurable length scales, which is inconsistent with our estimates of indel rates; this corresponds roughly to a maximum power law of *τ*=5 such that the grid fully explores the space of allowed instability rates.

To assess how closely each computationally modeled distribution *P*(*L*) (i.e., *P*( *L*, *t*| *m*, *τ_ϵ_*, *τ_κ_*) for each parameter combination {*m*, *τ_ϵ_*, *τ_κ_*} measured at the final time *t*=*T*) resembled the empirical distribution *P*_emp_(*L*), we defined the following metric. We compute the metric using the nonnormalized distributions (i.e., per genome counts at each length), denoted below as $\tilde{P}(\text{L})$ and ${\tilde{P}}_{emp}(\text{L})$ for the computationally modeled and empirical counts, respectively.


(Equation 1) $$metric=\sum_{L=1}^{L=L_{boundary}}{\left(log\left[Norm\left[\tilde{P}\left(L\right)+1\right]\right]-log\left[Norm\left[{\tilde{P}}_{emp}\left(L\right)+1\right]\right]\right)}^{2}$$

Here, *log* is the natural logarithm and *Norm* indicates that $\tilde{P}(\text{L})$ and ${\tilde{P}}_{emp}(\text{L})$ are normalized only after adding a pseudocount of one at each length. The pseudocount of one was added to each length class to appropriately handle bins with zero counts. The distribution was then normalized to approximate a probability distribution used to evaluate the metric. This is a least-squares distance metric in logarithmic space (i.e., taking the log of the distribution at each length *L*), which was chosen to upweight larger length classes that are more informative about the shape of the tail of the distribution relevant to repeat instability. In contrast, a likelihood-based measure, for example, upweights bins with the largest number of counts (i.e., the first few bins L=1, L=2, etc.), which are entirely driven by substitution-based dynamics and therefore uninformative about repeat instability rates. All information about the late-time length distribution of B strings was discarded. This metric value was computed for each parameter combination and minimized to identify the best fit parameter combination.

We then assessed the effect of statistical errors associated with our empirical estimates on our ability to identify parameter combinations consistent with the best fit metric value. The following procedure was used to generate errors associated with the repeat length distribution and subsequent metric value for the best fit parameters. First, for each motif, we estimated statistical errors associated with the empirically estimated mutation rates, by randomly sampling from a Poisson distribution with mean equal to the observed mutation counts; samples were obtained for each relevant three-unit context for substitutions and for each length bin for indels. For A_n_ motifs, this generated a sampled set of substitution rates and sampled expansion, contraction, and non-motif insertion rate curves for lengths *L*=1–8. We extended these rate curves using the parameter combination associated with the minimum metric value amongst those tested (i.e., the best fit parameter combination). Using these mutation rates, we repeated our computational procedure to produce a late time repeat length distribution reflecting these sampled rates. This procedure was repeated 200 times, each with a distinct set of sampled rate estimates. From the resulting distributions, we calculated two-sided 95% confidence intervals (CIs) separately around each length bin of the distribution by throwing out the top and bottom five values. Bootstrap errors associated with estimating the number of repeats in the genome (see Methods describing generation of empirical repeat length distributions) are negligible in comparison (Fig. 2c) and were not considered for subsequent analyses. To determine which parameter combinations were consistent with the minimum metric value, we then used each set of Poisson sampled rate curves and substitution rates to compute a metric value from the resulting distribution; a one-sided 95% CI was estimated by removing the top 10 metric values. Here, a one-sided CI was used, as all other parameter combinations have metric values above the minimum. Finally, we found the subset of parameter combinations consistent with the best fit parameters by identifying metric values within the 95% CI of the best fit value.

### Analytic modeling of repeat length dynamics

To better understand the underlying dynamics that generate with the genome-wide repeat length distribution, we attempted to analytically model the effect of each mutational type on the number of repeats at a given length *L* from first principles. We were interested in describing the steady state distributions that emerge for a subset of parameter combinations, as seen in the results of our computational model. Our goal was to capture the balance between relevant mutative forces, which can vary by repeat length, by writing an appropriate approximation to the steady state equation; the solutions to these equations describe the shape of the normalized repeat length distribution, *P*(*L*), restricted to the regime of validity of each approximation. Within this section, we have used the notation *P_L_* to represent the distribution *P*(*L*) more compactly when detailing the relevant equations. Each parameter combination defines a functional form for the per target (i.e., per unit) expansion, contraction, and non-motif insertion rates at lengths $L\geq 9:\epsilon (L\gg 1)=\epsilon_{0}L^{\tau_{\epsilon }}$, $\kappa (L\gg 1)=\kappa_{0}L^{\tau_{\kappa }}$, and $\iota (L\gg 1)=\iota_{0}L^{\tau_{\iota }}$, respectively, where the constants *ϵ*_0_, *κ*_0_ , and *ι*_0_ are set by the empirical value of these rates at *L*=8 and the multiplier *m* (noting that we set *τ_ι_* ≡ *τ_e_* to limit the number of free parameters; see inference Methods). Again, these length-dependent rates, in either discrete or continuous form, are denoted with a subscript *L* (e.g., *ϵ_L_* ≡ *ϵ*(*L*)) in this section for brevity. For substitutions, we refer herein to rates *μ* ≡ *μ*_A→B_ and *ν* ≡ *μ_B→A_* for lengthening and shortening mutations, respectively, but later specify separate mutation rates based on three-unit context (e.g., *μ_ABB→AAB_*) when comparing directly to computational model results. While the mutation rates may be well defined by these rates, the combined effect of substitutions and indels on the repeat length distribution requires a description of a number of complicated behaviors, including both local and non-local transitions between lengths across the distribution, non-conservation of the number of repeats due to fission and fusion, and non-linear dependence on the state of the distribution due to fusion (i.e., the generic dynamics are non-Markovian). As a result, our aim was not to describe an exact solution, but instead an expression for the effective dynamics that dominate the maintenance of the distribution in steady state, specifically in the asymptotic regimes associated with the shortest and longest length repeats. Note that this analytic description was motivated by and is strictly applicable to mononucleotide repeat dynamics, where the species of repeat length-changing mutations are fewer, but the conceptual findings may be generalizable to longer motif repeats (Fig. S3).

### Short repeat regime

First, we focused on the regime of asymptotically short repeats, as their behavior is more straightforward. By assessing the relative rates of substitution and indel processes in the estimated per-target rates (Fig. 2a), one can immediately see that substitutions must dominate the dynamics for the lowest length repeats. Short repeats can be entirely characterized by a straightforward balance between opposing types of substitutions, μ and *ν*, which is equivalent to sequence evolution under a two-way point mutation process. At steady state, the resulting distribution is equivalent to the probability of randomly assembling specific strings of length *L* when the whole genome is randomly sampled between A and B bases with probability *p*_*A*_ = *μ*/(*μ* + *ν*) and *p*_*B*_ = *ν*/(*μ* + *ν*), respectively. The frequency of a length *L* string of A’s (i.e., an A repeat) is geometrically distributed in proportion to ${p_{A}}^{L}$ (i.e., sampling an A, *L* successive times).


(Equation 2) $$P_{L\ll 10}\propto {\left(\frac{\mu }{\mu +\nu }\right)}^{L}$$

Here, we have omitted a normalization constant that determines the relative weight of this geometric distribution to the weight of the long repeat tail. For comparison to the computational model (or the empirical distribution), we fixed the normalization constant using the mass of the *L* = 1 bin. The approximation that the effects of expansion, contraction, and non-motif insertion are negligible breaks down at a length determined by the estimated relative rates in Fig. 2a; the regime of validity for this approximation extends roughly to lengths of order *L* = 10.

### Long repeat regime

The dynamics of long repeats, i.e., for asymptotically large repeat lengths *L* ≫ 1, the analysis is complicated by the numerous length-dependent (and parameter-dependent) forces that can potentially contribute to stabilizing the distribution. While expansion and contraction describe inherently local transitions from *L* to *L* + 1 and from *L* to *L* − 1, respectively, the effects of non-motif insertions and substitutions on extended repeats are not strictly local. To model this regime, we first wrote a finite difference equation that describes the change in the distribution in a single time step Δ*t*: Δ*P*_*L*_ ≡ *P_L_*(*t* + Δ*t*) − *P_L_*(*t*), where P_L_(t) = *P*(*L*, *t*; μ, ν, ϵ*_L_*, κ*_L_*, ι*_L_*) is implicitly dependent on the length scaling of each rate (see SN1). From this discrete equation, we derived a partial differential equation (PDE) in the large-length continuum limit Δ*L* = 1 ≪ *L* that approximates the dynamics in the large length regime (derivation provided in SN1). This PDE includes explicit terms depicting the combined local effects of repeat instability due expansion and contraction, each occurring at distinct length-dependent rates, and the separate effects of repeat fission and fusion, each introducing an integral that captures the aggregate effects of non-local transitions in length. Expansion and contraction collectively generate both symmetric (i.e., bidirectional) and asymmetric local length transitions, which correspond to a diffusion term represented by a second derivative and directional flux term expressed as a first derivative, respectively, each appropriately accounting for length dependent rates.

While local effects from substitutions and non-motif insertions exist (specifically, transitions *L* → *L* + 1 or *L* → *L* − 1), as well, they are negligible in comparison to expansion and contraction due to their low relative rates at long lengths and finite target size of two per repeat. Fission due to substitutions and non-motif insertions were both accounted for as separate non-local contributions to the change in *P_L_*. Importantly, the probability of fission due to substitution is proportional to the target size (*L* − 2) ≈ *L*; for insertions, the rate itself harbors an additional length dependence such that the per-repeat rate of fission scales as *L*^1+*τ_ϵ_*^. As a result, the relative importance of fission compared to local contributions is highly dependent on the parameters *τ_ϵ_* and *τ_κ_*; similarly, the relative importance of substitution- and insertion-based fission are parameter dependent due to distinct dependencies on length. Thus, a unified description across parameter space requires the inclusion of fission in full form and captures all four mutational effects. While we were able to explicitly describe the integral effects of length changes due repeat fusion in the continuum (see SN1), the inherent non-locality is additionally complicated by the nonlinearity introduced by pairing two repeats randomly sampled from the distribution. To make further progress, we proceeded under the assumption that fusion remains subdominant at large lengths, which we confirmed via our computational model to be generally true for parameters consistent with the empirical distribution. Stochastic fluctuations in the mutation rates were omitted, resulting in a deterministic approximation for the expected repeat length distribution.

Next, we imposed the assumption of steady state (i.e., *dP*/*dt* = 0), reducing the PDE to an ordinary differential equation in length to solve for the shape of the distribution in equilibrium. Despite excluding complications from fusion, the remaining approximation to the steady state equation is, strictly speaking, a second order integro-differential equation, for which no explicit closed-form solutions exist. The following equation approximates the steady state dynamics in the absence of fusion (i.e., when fusion is subdominant). Here, *∂_x_* represents a derivative with respect to *x* (noting that partial derivatives with respect to *L* become total derivatives in steady state) and *P_L_* is the steady state value of the continuous repeat length distribution at large length *L* ≫ 1 up to an overall normalization constant (along with an arbitrary constant set to zero). Again, all continuous functions describing mutation rates (e.g., ϵ_*L*_, κ_*L*_) are expressed here as per-target rates.


(Equation 3) $$\frac{dP_{L}}{dt}=0\approx \frac{1}{2}\partial_{L}^{2}\left[\left({\text{ϵ}}_{L}+\kappa_{L}\right)LP_{L}\right]-\partial_{L}\left[\left({\text{ϵ}}_{L}-\kappa_{L}\right)LP_{L}\right]-\left(\text{ν}+{\text{ι}}_{L}\right)LP_{L}+2\int_{L}^{\infty }d\lambda \left(\text{ν}+{\text{ι}}_{\text{λ}}\right)P_{L}$$

In order from left to right, the terms appearing on the right hand side describes: length-dependent diffusion (arising from local transitions due to expansion and contraction), a length-dependent local directional flux (due to the bias between expansion and contraction), a net loss of due to fissions that break up length *L* repeats (i.e., substitutions or insertions that interrupt the repeat sequence; referred to herein as *fission out*), and a net gain due fissions of repeats longer than *L* (referred to as *fission in*). Fission in represents the sole integral effect, which substantially complicates our analysis; elimination of the integral dependence is discussed below and results in a third order ordinary differential equation (ODE) that maps to this second order integro-differential equation.

### Contraction-biased rates stabilize the distribution

Importantly, we found that steady state could only be reached for the subset of parameter combinations with *τ_κ_* > *τ_ϵ_*, corresponding to cases for which local transitions are asymptotically contraction-biased: $\underset{L\to \infty }{\lim}\left(\kappa_{L}-{\text{ϵ}}_{L}\right)>0$ (note that the edge case where *τ_κ_* = *τ_ϵ_* is asymptotically expansion-biased based on observations at *L* = 8 and implications of our parameterization). We therefore denote this as the contraction-biased regime, which is characterized by defining the variable Δτ ≡ τ_κ_ − τ*_ϵ_*. When Δτ > 0, the distribution is stabilized at some arbitrarily large length *L* = *L_max_* by sufficiently large contraction rates in excess of all processes that increase repeat length; a truncation of the distribution (i.e., when less than one repeat is expected in a genome of given size) occurs due to the more rapid increase of contraction rates than expansion rates that leads to contraction-biased dynamics at some point *L* < *L_max_*. The necessity of asymptotic contraction-bias contrasts the notion that length-dependent interruptions (due to substitutions and non-motif insertions) counteract expansion at sufficiently long lengths, stabilizing the distribution^36,42,48,50–52^; based on our estimated mutation rates, this effect does not lead to a steady state in the absence of contractions, as the per-repeat rate of expansions far exceeds that of repeat fission (i.e., interruptions) at long lengths. As discussed below, the length at which the contraction rate is equal to the expansion rate L* (i.e., L* is the unique length *L* ≥ 8 where κ*_L_* = ϵ_*L*_, which may occur at non-integer values) is highly informative about the dynamics in each regime, as well as the behavior when all effects captured in Equation 3 are simultaneously relevant; L* is exponentially dependent on Δ*τ* and more weakly controlled by the multiplier *m*, notably occurring at the same length across lines of constant Δ*τ* in the parameter space (for a given *m*). For *m* ≫ 1, the dynamics are nearly identical for parameter combinations with the same Δτ, effectively collapsing the {*τ_ϵ_*, τ_κ_} plane to a single dimension. The functional dependence of L* on the parameters and further discussion is provided in Supplementary Note SN1.

### Effective equations approximating steady state dynamics

Given the complexity of Equation 3 introduced by the nonlocal effects of fission, we first searched for subsets of the contraction-biased parameter space that could be well approximated under a further reduction of the dynamics. Such simplifications are, in principle, possible because the length scaling of each term in Equation 3 is distinct; specifically, parameter combinations exist where the nonlocal behavior (i.e., the integral representing fission in) becomes subdominant and can be neglected in our analysis. Neglecting the integral results in a second order ODE approximation to the steady state equation. We identified two distinct dynamical regimes within the Δ*τ* > 0 region, which are each well-approximated by a subset of contributions that dominate the dynamics in their respective regimes of validity.

### Balance between local dynamics in the highly contraction-biased regime

For parameter combinations with very large positive values of Δ*τ* (i.e., for *τ_κ_* ≫ *τ_ϵ_*), the dynamics are entirely dominated by the diffusion and local directional flux terms appearing in Equation 3, as the contraction rate quickly outcompetes both the rate of fission in and fission out. This results in an effective steady state equation dominated only by local transitions.


(Equation 4) $$\frac{1}{2}\partial_{L}^{2}\left[\left(\epsilon_{L}+\kappa_{L}\right)LP_{L}\right]-\partial_{L}\left[\left(\epsilon_{L}-\kappa_{L}\right)LP_{L}\right]\approx 0$$

In this case, the contraction rate exceeds the expansion rate almost immediately above the short length regime (i.e., L* is of order 10) such that the dynamics are effectively uniform across the long length regime. The long length tail of the distribution decays in a super-exponential fashion such that the truncation occurs at low values of *L*_max_ ~ 20, which dramatically limits the lengths of repeats that occur in a genome of realistic size. In this regime, a further simplification leads to an approximate closed-form analytic solution for the rough asymptotic shape of the distribution, however this approximation is only valid near the truncation point and rapidly loses accuracy. A more general solution was obtained by numerically solving the effective steady state equation (Equation 4) for comparison to computational model results. To obtain numerical values, two additional constraints must be applied, as with any second order ODE, which conceptually correspond to an overall normalization constant (in this case, fixing the relative weights of the short length and long length distributions) and a linear coefficient that defines the relative weights of two real solutions, if both exist. These constraints can be imposed by fixing the value of the distribution at two specific lengths, *L*_1_ and *L*_2_, (i.e., fixing $P_{L_{1}}=P_{L_{1}}^{comp}$ and $P_{L_{2}}=P_{L_{2}}^{comp}$, where $P_{L}^{comp}$ is the value of the computationally modeled distribution at length *L*), with both lengths chosen to lie long length regime *L* > 10 where the continuum approximation remains valid. For consistency, we chose to constrain the numerical solutions at the two lengths of theoretical interest in stable distributions: *L*_1_ = L* (rounded to the nearest integer) and *L*_2_ = *L*_max_, both of which definitionally remain in the long length regime at a location with finite occupancy in a realistic genome and are well defined for all values of Δτ > 0. All numerical solutions were obtained using the *NDSolve* function in Mathematica 14.0^84^. Comparisons between computational model results and numerical solutions to Equation 4 showed that this approximate steady state equation remains highly accurate across the Δτ ≫ 1 regime (see SN1).

### Relevant effects of fission out in the intermediate contraction-biased regime

We found that at less extreme values of Δτ, roughly on the order of Δτ ~ 1, the integral contributions to Equation 3 remained subdominant, but the effects of fission could not be omitted completely. In this regime, fission out non-negligibly impacts the dynamics, leading to an effective steady state equation that only omits incoming contributions from fission.


(Equation 5) $$\frac{1}{2}\partial_{L}^{2}\left[\left(\epsilon_{L}+\kappa_{L}\right)LP_{L}\right]-\partial_{L}\left[\left(\epsilon_{L}-\kappa_{L}\right)LP_{L}\right]-\left(\nu +\iota_{L}\right)LP_{L}\approx 0$$

In this regime, contraction is aided by the length-reducing effects of fission out. However, the relevance of this contribution is limited roughly to lengths below L* ; above L*, the distribution remains well-described by Equation 4 (see SN1). This indicates that contraction is largely responsible for truncating the distribution, even when fission is involved in shaping the distribution. This defines a range of intermediate lengths below L* with distinguishable dynamics from asymptotic lengths, but this range is limited by the relatively small values of L* on the order of L* ~ 15 – 20. The approximation in Equation 5 is again a second order ODE but is complicated by the introduction of an additional length scaling associated with substitution-based fission. However, even when substitution rates are negligible (e.g., for *m* ≫ 1), no exact solution could be found due to the generic power laws associated with our parameterization. For comparison to the computational model, numerical solutions were obtained by again constraining the solution at lengths *L*_1_ = L* and *L*_2_ = *L*_max_. We found that the effective steady state equation (Equation 5) is a highly accurate approximation to the dynamics in this regime of moderate values of Δτ. Additionally, this approximation remains accurate at large values of Δ*τ* (i.e., Equation 5 is applicable to the full subspace Δτ ≳ 1), as the approximation in Equation 4 is nested in Equation 5; the latter includes the additional effect of fission out, which becomes negligible for Δ*τ* ≫ 1.

### Inclusion of the nonlocal dynamics in the weakly contraction-biased regime

For values Δτ < 1, the nonlocal effects described by the integral term in Equation 3 become relevant to the maintenance of steady state. To further analyze this regime, we first eliminated the integral dependence by applying an overall length derivative to all terms on the right-hand side of Equation 3 such that the equation becomes the following.


(Equation 6) $$\partial_{L}\left[\frac{dP_{L}}{dt}\right]=0\approx \frac{1}{2}\partial_{L}^{3}\left[\left(\epsilon_{L}+\kappa_{L}\right)LP_{L}\right]-\partial_{L}^{2}\left[\left(\epsilon_{L}-\kappa_{L}\right)LP_{L}\right]-\partial_{L}\left[\left(\nu +\iota_{L}\right)LP_{L}\right]-2\left(\nu +l_{L}\right)P_{L}$$

This third order ODE now represents a constraint on the net flux, which must equal a time-independent constant. This can be seen by swapping the order of the derivatives on the left-hand side of Equation 6: *∂_L_* [*dP_L_/dt*] = *d*[*∂_L_P_L_*]/*dt* = 0. Taking this overall length derivative maps the nonlocal contributions from the fission of all repeats longer than L to an effectively local boundary effect on the net flux *∂_L_P_L_* through length *L*. However, this is not equivalent to steady state until applying an additional constraint that this net flux vanishes (i.e., the special case where the constant is zero, *∂_L_P_L_* = 0). Obtaining numerical solutions to this third order ODE requires three constraints, which now includes the constraint that the net flux vanishes. For comparison to the computational model, this was imposed by again specifying *L*_1_ = L* and *L*_2_ = *L*_max_ along with the additional constraint $P_{L_{3}}=P_{L_{3}}^{comp}$ at length $L_{3}=L_{2}-1$, chosen for convenience. We found good agreement between the resulting numerical solutions and our computational model results. Additionally, solutions to this equation accurately describe the parameter regimes that are well approximated by Equations 4 and 5, as the latter represent nested dynamics characterized by Equation 6 that discard negligible contributions. Thus, Equation 6 has a regime of validity that extends across the entire set of parameter combinations that result in stable distributions Δτ > 0. As a corollary, the accuracy of this approximation to the full steady state dynamics across the space of computational model results indicates that the effects of repeat fusion remain negligible throughout. However, this statement is only applicable to the long repeat dynamics for L>10; the effects of repeat fusion are everywhere relevant for short repeats, which, in part, shape the geometric distribution at steady state.

Details on the derivation, relevant approximations, dynamical regimes, and comparison between numerical and computational model results are provided in depth in Supplementary Note SN1.

## Supplementary Material

Supplement 1

Supplement 2

3

## Data Availability

The datasets analyzed during the current study are freely available from the NCBI (https://www.ncbi.nlm.nih.gov/datasets/genome/), the UCSC Genome Browser (https://genome.ucsc.edu), and other studies as cited. Instructions for accessing specific datasets are further detailed in the code repository (see Code Availability). Repeat length distributions for mammalian genomes analyzed in this study are available in Supplementary Data File SF1. Repeat length instability rates calculated in this study are available in Supplementary Data File SF2.
