# Supplementary material for: Inherent instability of simple DNA repeats shapes an evolutionarily stable distribution of repeat lengths
Source: bioRxiv. 2025 Jan 10:2025.01.09.631797. Preprint. [Version 1] doi: 10.1101/2025.01.09.631797 (PMC11741425; doi:10.1101/2025.01.09.631797)
Supplement: 3 [file NIHPP2025.01.09.631797v1-supplement-3.pdf]

bioRxiv preprint doi: https://doi.org/10.1101/2025.01.09.631797; this version posted January 10, 2025. The copyright holder for this preprint (which was not certified by peer review) is the author/funder, who has granted bioRxiv a license to display the preprint in perpetuity. It is made available under aCC-BY 4.0 International license.

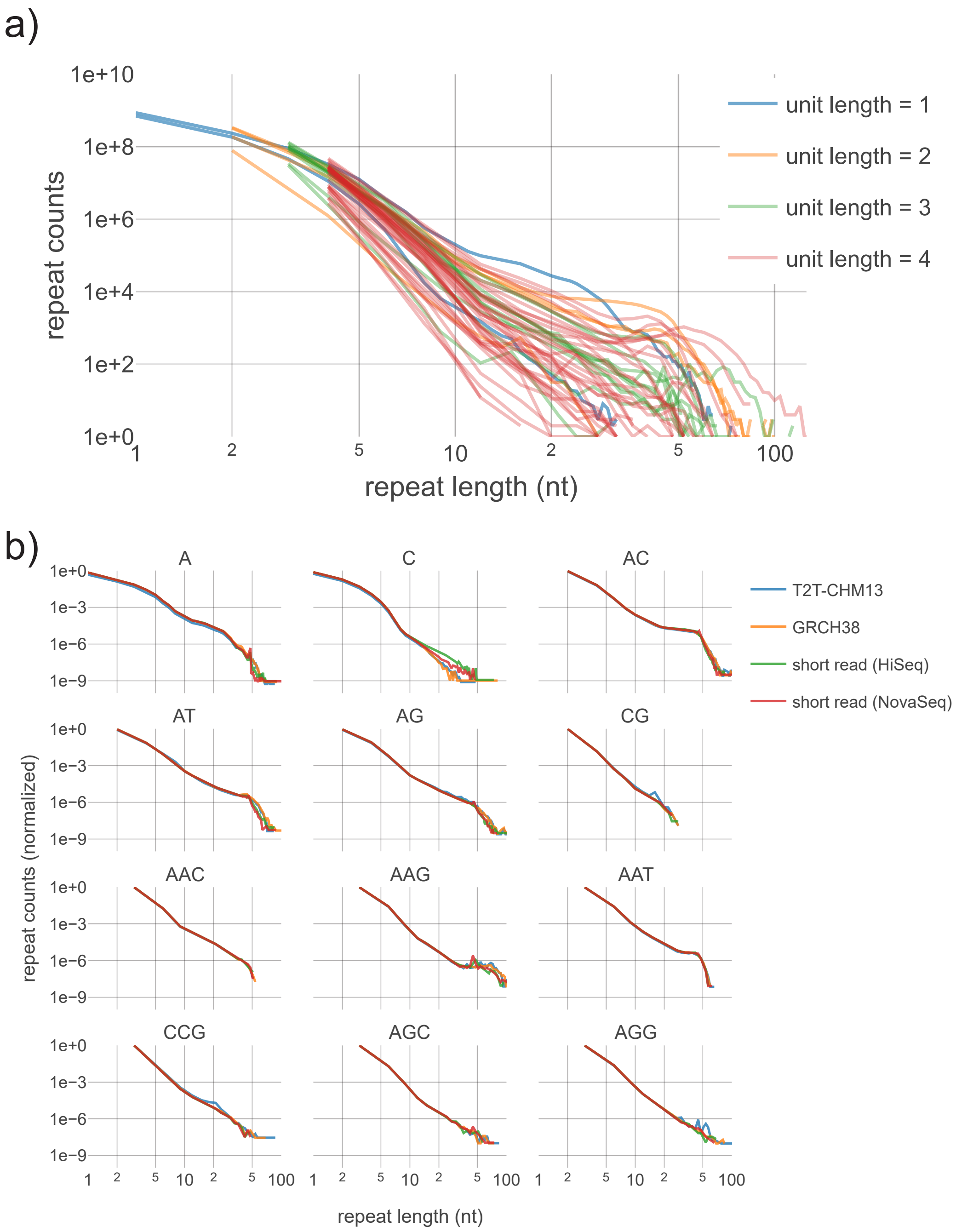

Fig. S1. *Repeat length distributions per motif and per sequencing technology.* **a)** Non-normalized distribution of repeat lengths for all distinct motifs for unit lengths 1-4. Motifs of the same unit length are shown in the same color. Distributions vary by motif but share similar qualitative features. **b)** Normalized distribution of repeat lengths in four distinct human genome assemblies with different sequencing technologies. T2T-CHM13 was assembled using multiple long read technologies, while other assembles employed shorter read technologies. Read length does not appear to affect the accurate counting of repeats of sub-disease lengths.

bioRxiv preprint doi: https://doi.org/10.1101/2025.01.09.631797; this version posted January 10, 2025. The copyright holder for this preprint (which was not certified by peer review) is the author/funder, who has granted bioRxiv a license to display the preprint in perpetuity. It is made available under aCC-BY 4.0 International license.

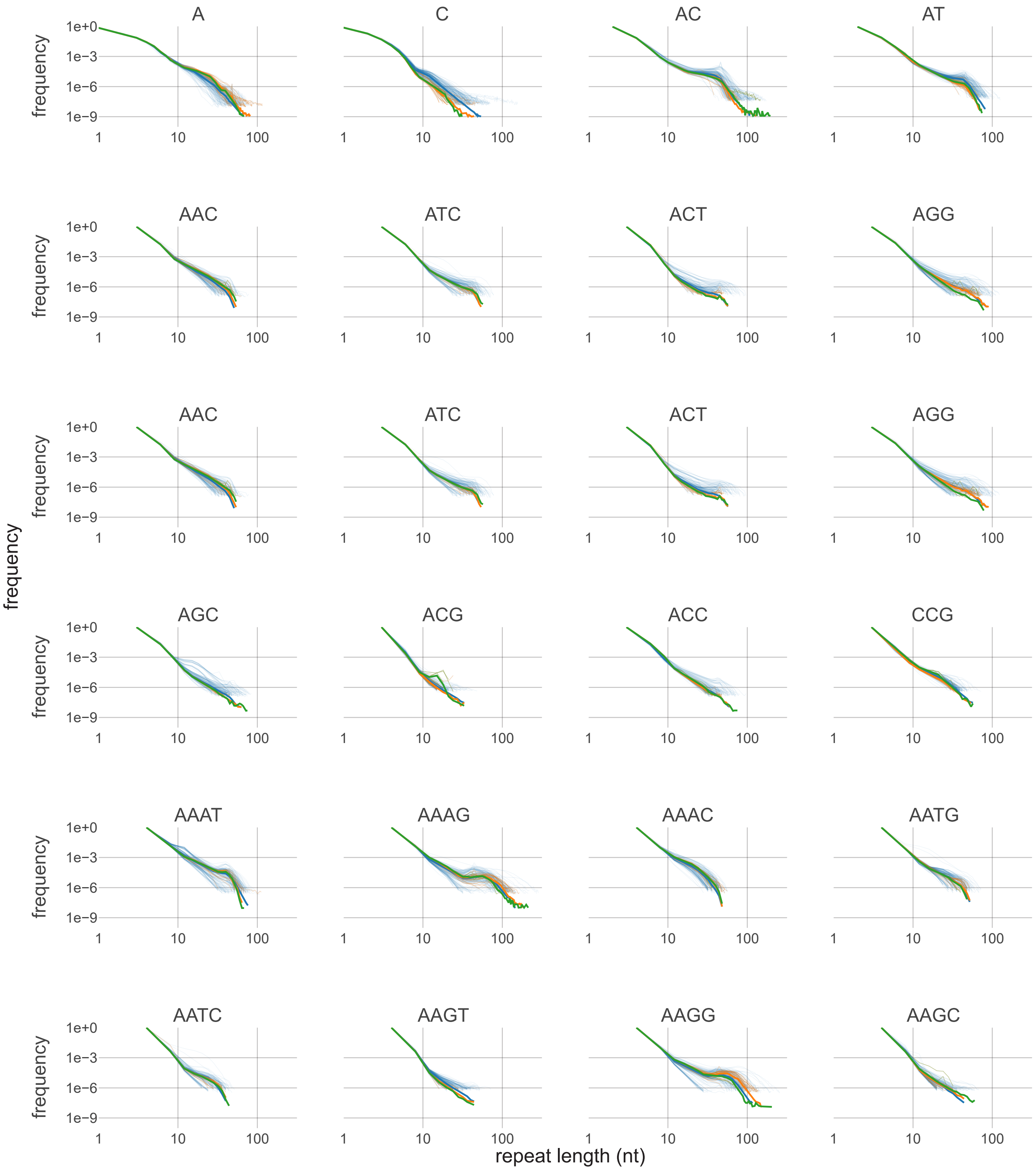

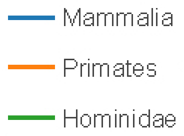

Fig. S2. *Repeat length distributions per motif across phylogenies.* Normalized distributions of repeat lengths for various motifs in mammals, primates and hominids. Solid line indicates median values per length bin for each phylogeny. Thin transparent lines show individual species within the phylogeny. Individual distributions are cut off at the shortest bin containing 30 counts. Phylogenies are inclusive (e.g., primates are included as a subset of mammals). Overlapping medians between primates and hominids suggests long-term stability of the distribution, while individual mammalian species display variability.

bioRxiv preprint doi: https://doi.org/10.1101/2025.01.09.631797; this version posted January 10, 2025. The copyright holder for this preprint (which was not certified by peer review) is the author/funder, who has granted bioRxiv a license to display the preprint in perpetuity. It is made available under aCC-BY 4.0 International license.

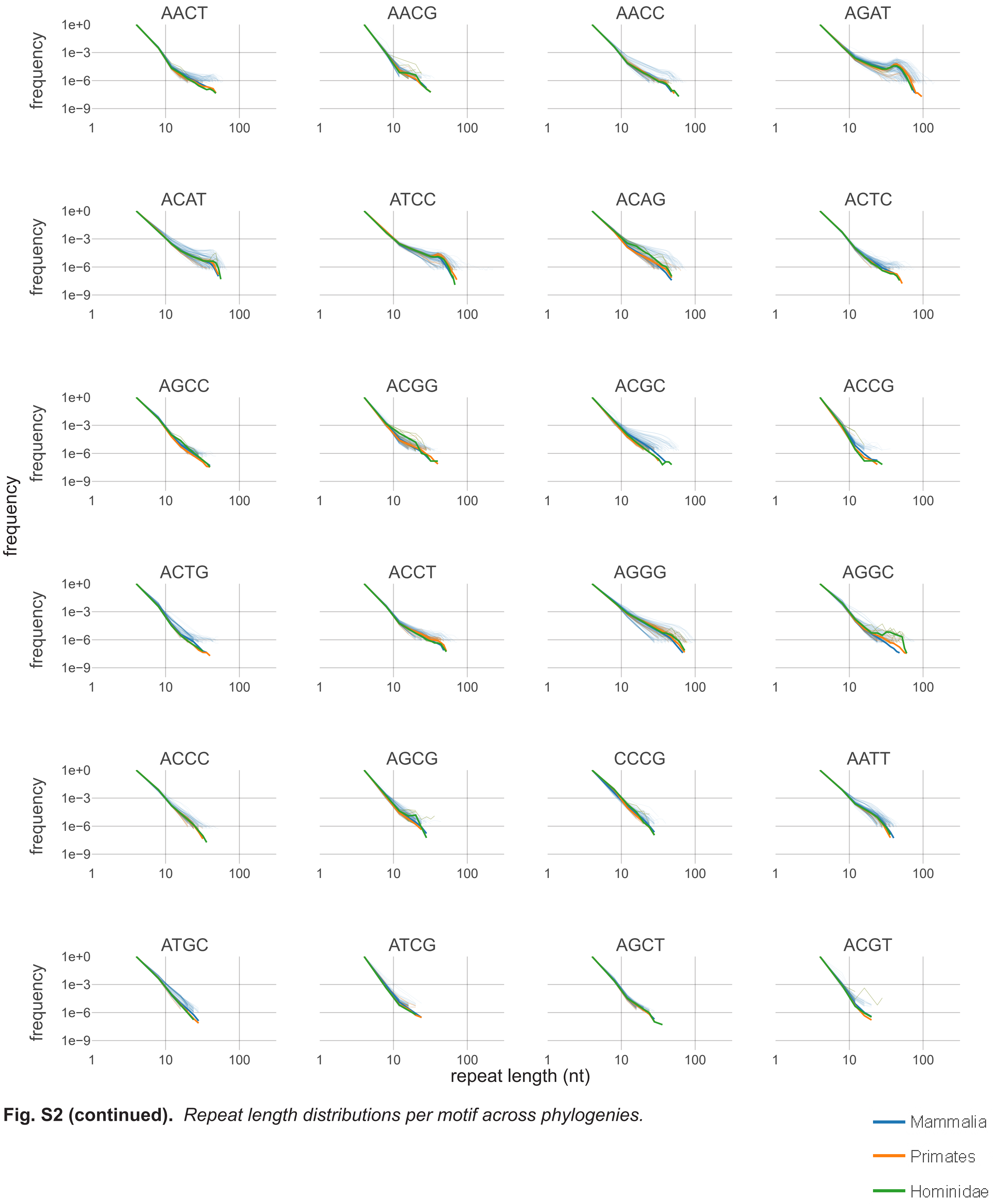

Fig. S2 (continued). *Repeat length distributions per motif across phylogenies.*

bioRxiv preprint doi: https://doi.org/10.1101/2025.01.09.631797; this version posted January 10, 2025. The copyright holder for this preprint (which was not certified by peer review) is the author/funder, who has granted bioRxiv a license to display the preprint in perpetuity. It is made available under aCC-BY 4.0 International license.

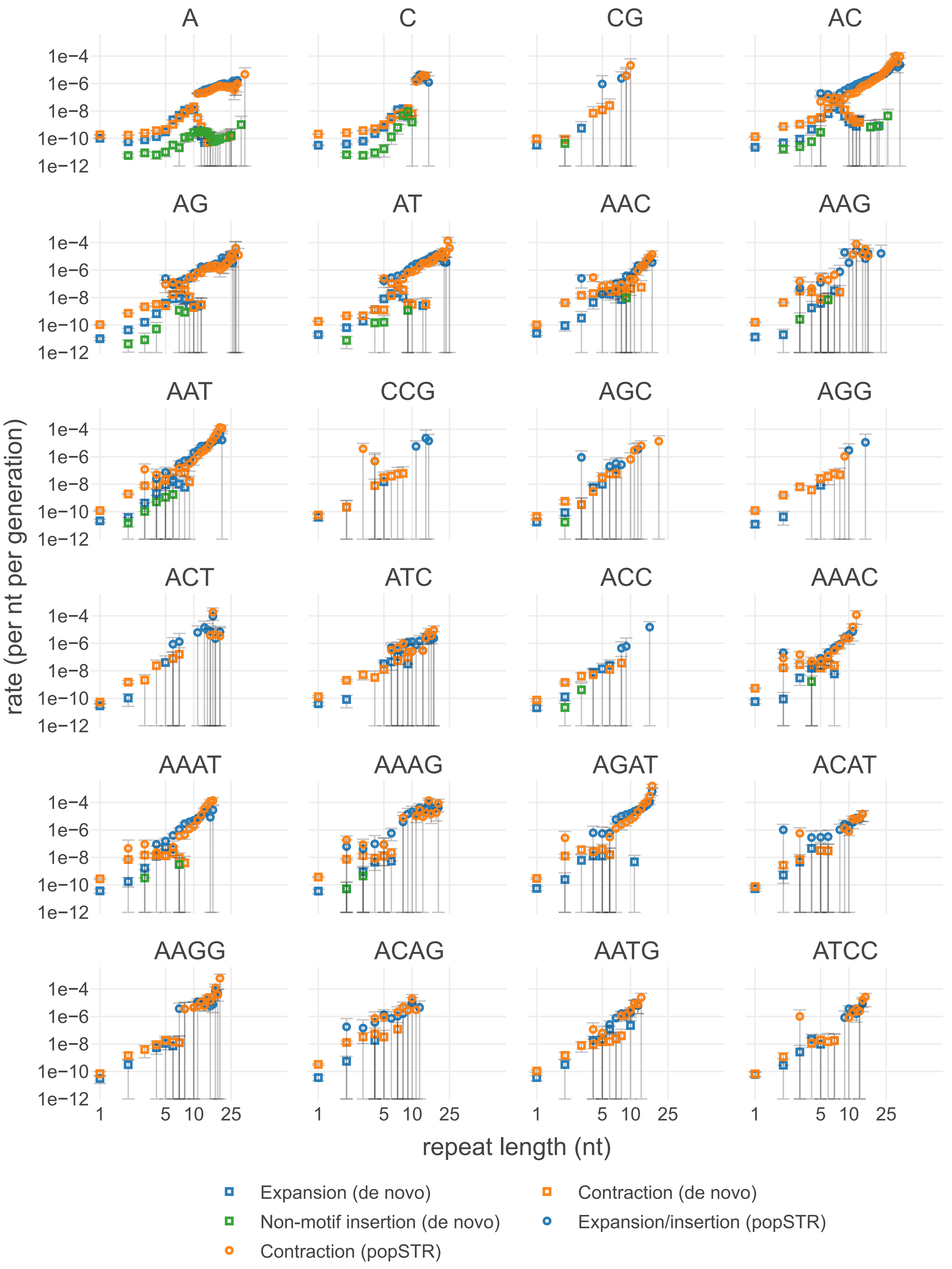

Fig. S3. *Instability rate estimates*. Separate rate estimates from de novo and popSTR datasets for expansion, contraction and non-motif insertions for various repeat motifs. Statistical error bars show 95% confidence intervals.

bioRxiv preprint doi: https://doi.org/10.1101/2025.01.09.631797; this version posted January 10, 2025. The copyright holder for this preprint (which was not certified by peer review) is the author/funder, who has granted bioRxiv a license to display the preprint in perpetuity. It is made available under aCC-BY 4.0 International license.

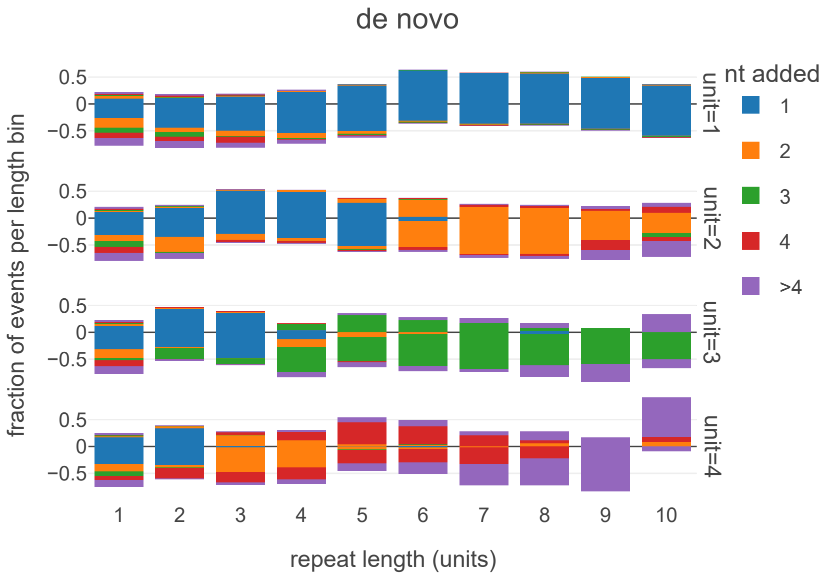

Fig. S4. *Nucleotides gained or lost per indel event.* Indels measured from two different data sources: de novo indels from trio sequencing (**top**) and popSTR estimates (**bottom**). Row labels indicate the motif unit length. Color indicates how many nucleotides were gained or lost per indel event. X-axis displays the length of the repeat in number of units. Y-axis displays the fraction of events per length bin. Y-axis sign indicates insertion (positive) or deletion (negative); overall shift up or down indicates bias. Plots show that most events involve the gain or loss of a single complete unit, independent of total repeat length; both partial-unit and multiple-unit changes are less frequent. A length threshold is visible, above which repeat instability exceeds the background indel process. Note that accurate calling of indels in the de novo dataset is reduced for STRs above 10 nt; likewise, the popSTR database is not sufficiently populated below 10 nt and above 30 nt.

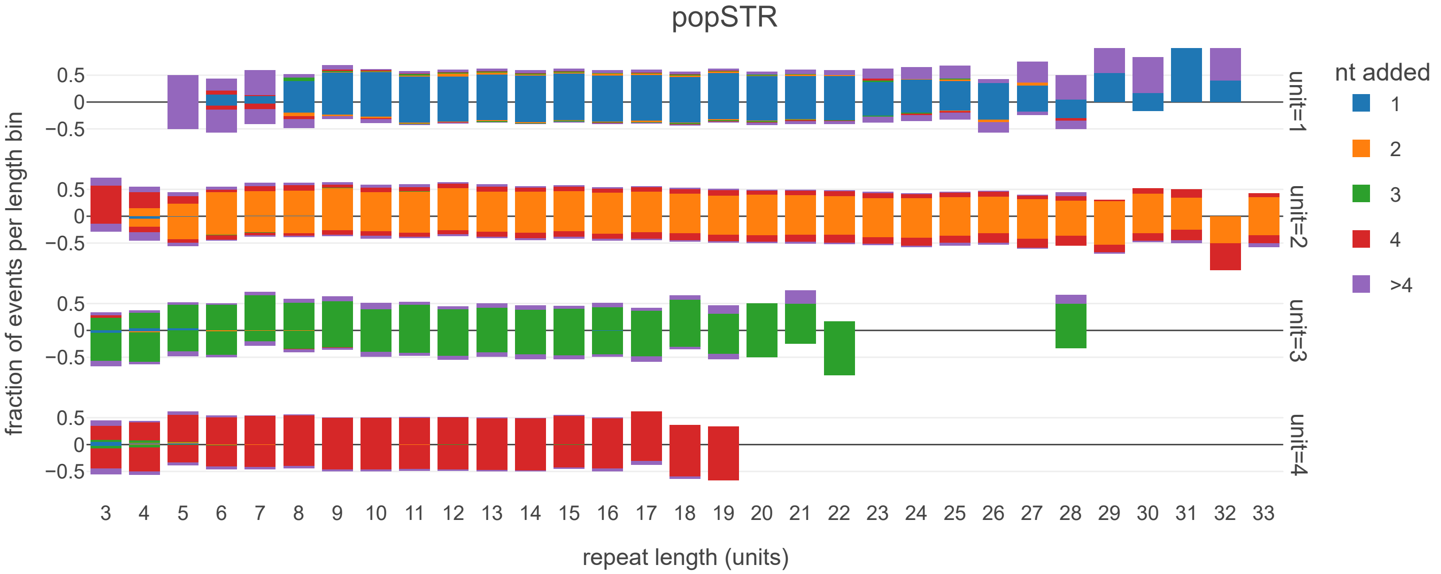

bioRxiv preprint doi: https://doi.org/10.1101/2025.01.09.631797; this version posted January 10, 2025. The copyright holder for this preprint (which was not certified by peer review) is the author/funder, who has granted bioRxiv a license to display the preprint in perpetuity. It is made available under aCC-BY 4.0 International license.

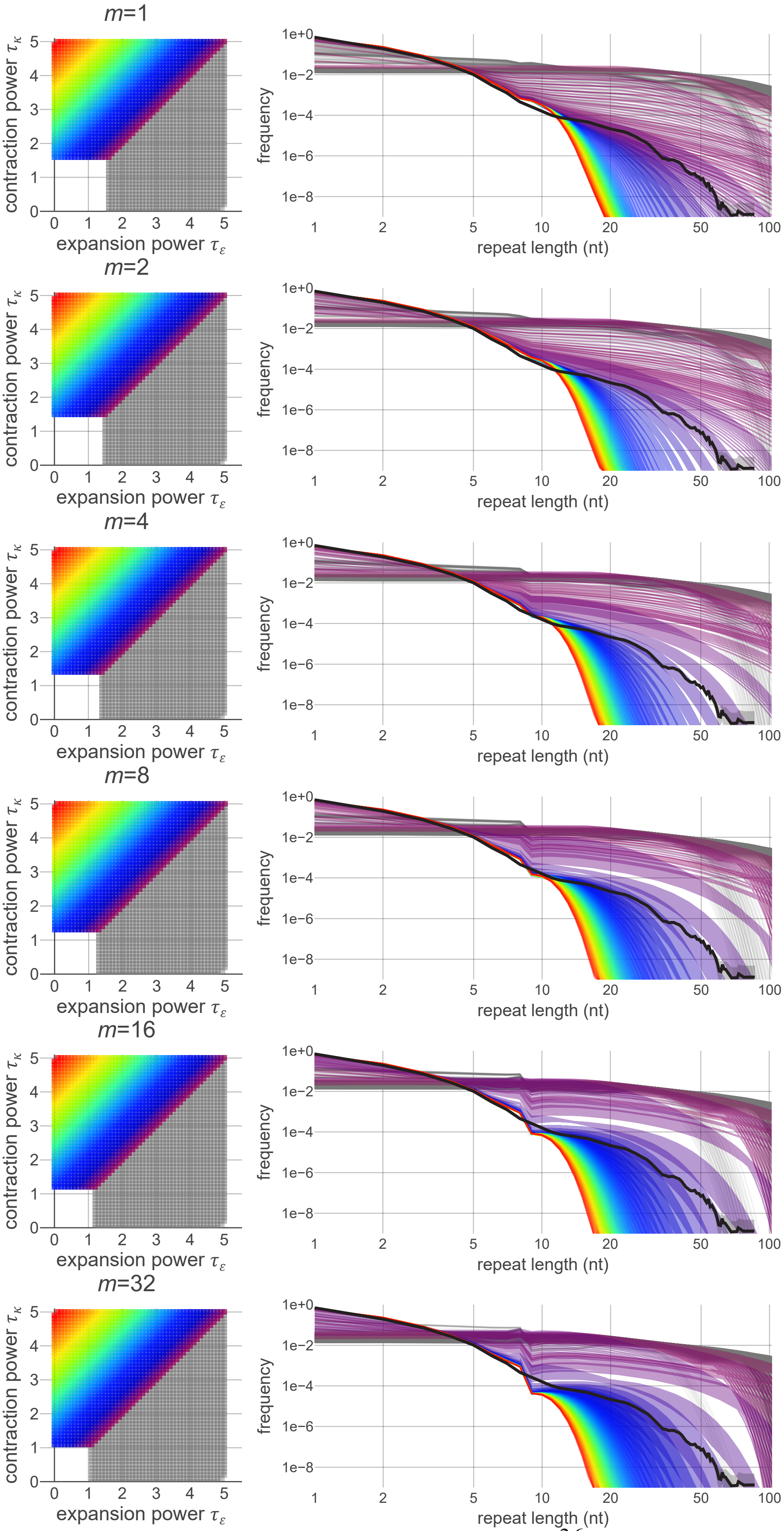

Fig. S5. *Final time point distributions from computational model across parameter space.* Lines of constant $\Delta\tau=\tau_\kappa-\tau_\varepsilon$ are shown in the same color. Red corresponds to large values of $\Delta\tau$, purple corresponds to low values of $\Delta\tau$, and gray represents negative values of $\Delta\tau$. **(Left)** Grid of $(\tau_\varepsilon,\tau_\kappa)$ parameter values for various multipliers *m*. For clarity, lower left region is not shown due to low rates insufficient to equilibrate in the alotted time. **(Right)** Plots of A_n repeat length distributions at the final time point, one for each point in the grid. Black line depicts the empirical distribution. Larger $\Delta\tau$ results in more rapid truncation of the distribution at lower lengths; smaller values of $\Delta\tau$ result in a more extended tail of long repeats.

bioRxiv preprint doi: https://doi.org/10.1101/2025.01.09.631797; this version posted January 10, 2025. The copyright holder for this preprint (which was not certified by peer review) is the author/funder, who has granted bioRxiv a license to display the preprint in perpetuity. It is made available under aCC-BY 4.0 International license.

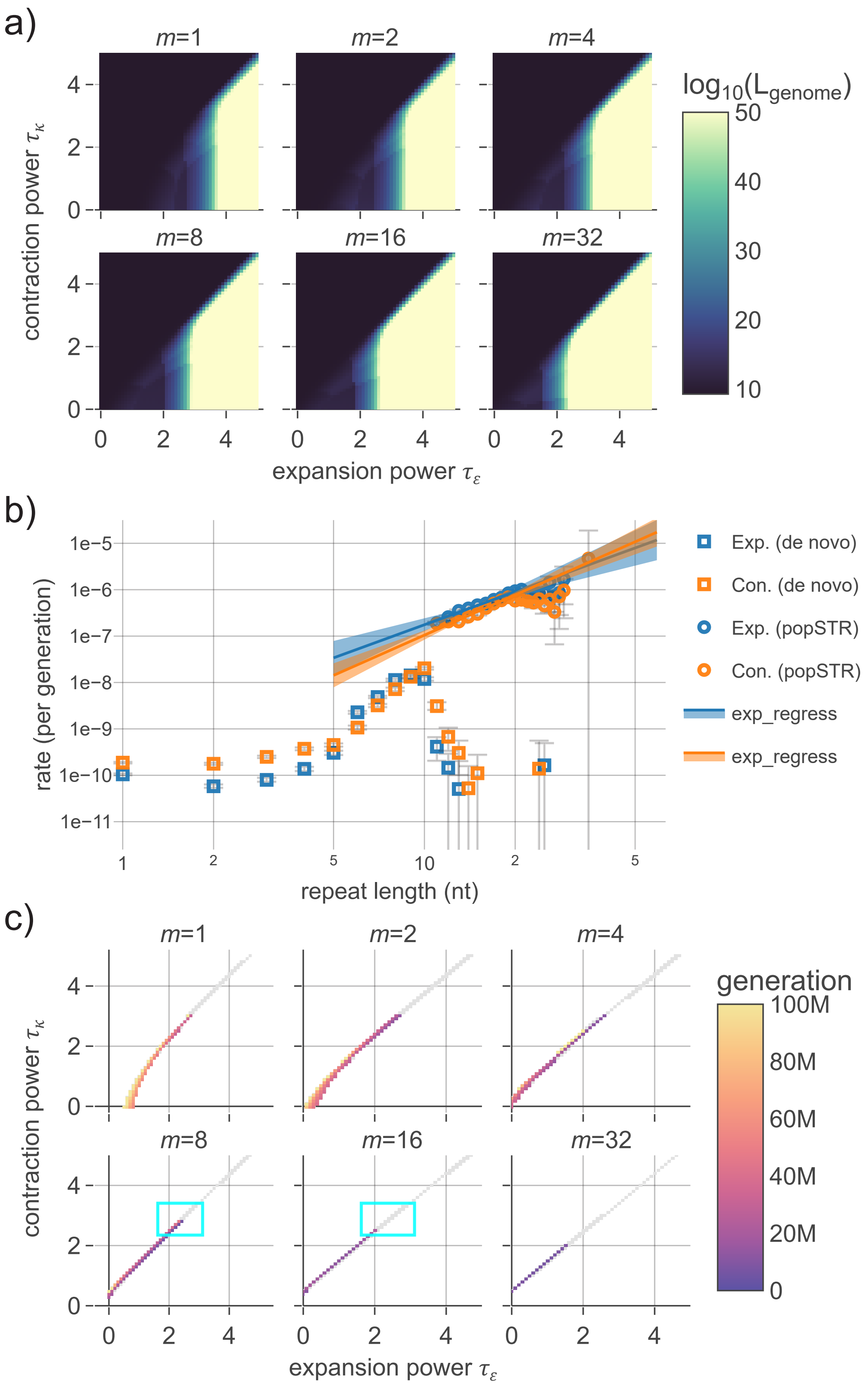

Fig. S6. *Constraints on realistic parameter values.* **a)** Plot of total number of repeat bases in genome at final time point across parameter space. Color specifies $\log_{10}$ of genome-wide count of A bases. Color range is truncated at 10^{50}. Computational model results in explosive genome growth for expansion-biased parameter combinations. **b)** Rate estimates from de novo and popSTR datasets for expansion, contraction and non-motif insertions for A_n repeats. Lines represent linear regression in log-log space of the popSTR expansion and contraction rate estimates for *L*=12-19. Statistical error bars show 99.9% confidence intervals, which suggest empirical bounds on parameters *m*, τ_ε and τ_κ. **c)** Plot of timepoint when each parameter combination becomes consistent with the best fit parameters. Only parameter values consistent at the final time point are shown. Here, a constant time rescaling was used, rather than progressive time rescaling. Blue boxes show 95% confidence intervals resulting from (b). The small subset of parameter combinations within these boxes is simultaneously consistent with de novo and popSTR rates estimates of repeat instability, the empirical distribution of repeat lengths in humans, and the convergence to steady state within realistic timescales. Grey points are consistent with the best-fit parameter combination but required progressive time-rescaling due to computational limitations, precluding generation time measurement.

bioRxiv preprint doi: https://doi.org/10.1101/2025.01.09.631797; this version posted January 10, 2025. The copyright holder for this preprint (which was not certified by peer review) is the author/funder, who has granted bioRxiv a license to display the preprint in perpetuity. It is made available under aCC-BY 4.0 International license.

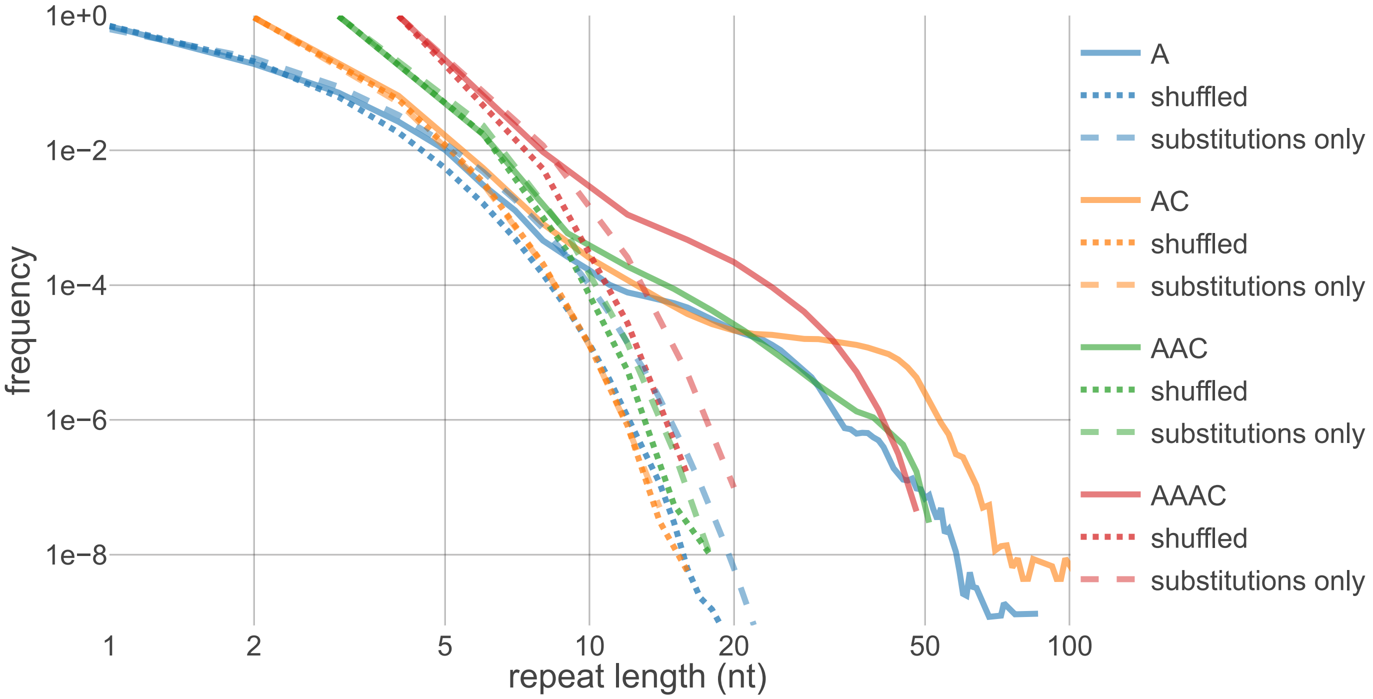

Fig. S7. *Assessing the onset of repeat instability for longer motifs.* Solid lines display normalized repeat length distributions in CHM13-T2T for several example motifs, highlighting the difference between mono-, di-, tri- and tetranucleotide motifs. Dotted lines represent counts in a randomly-shuffled human genome sequence. Dashed lines represent a computational model of the substitution process alone (omitting all indels). This results in a geometric distribution, which describes the low-length (i.e., <10 nt) portion of each empirical distribution. Empirical deviation from this distribution results from the relevance of repeat instability at longer lengths, roughly above 10 nt, independent of motif length.

bioRxiv preprint doi: https://doi.org/10.1101/2025.01.09.631797; this version posted January 10, 2025. The copyright holder for this preprint (which was not certified by peer review) is the author/funder, who has granted bioRxiv a license to display the preprint in perpetuity. It is made available under aCC-BY 4.0 International license.

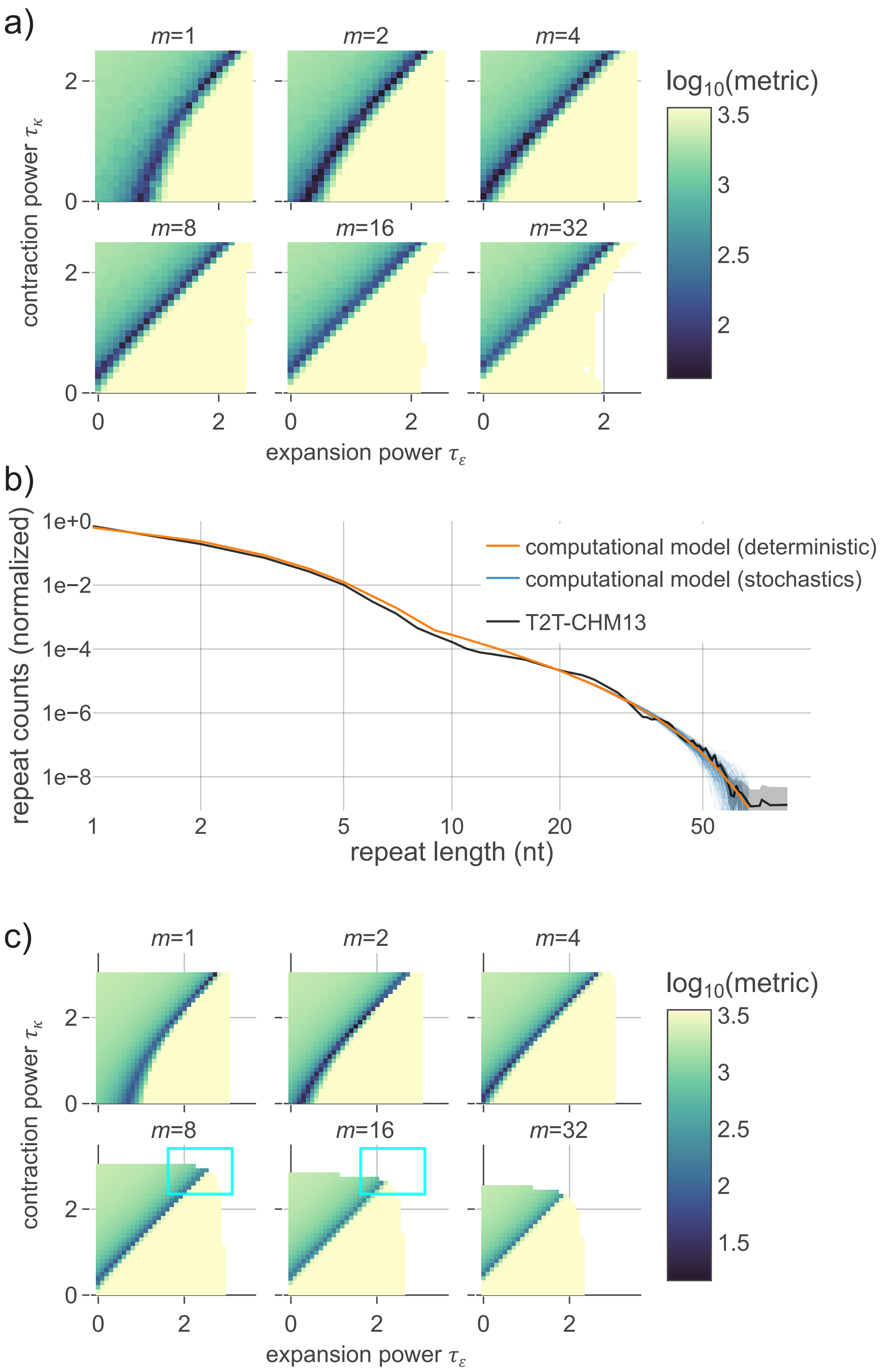

Fig. S8. *Tests of simplifying assumptions used in the computational model.* **a)** Metric plots for a computational model that incorporates stochastic fluctations. In each generation, the number of mutational transitions between length classes was Poisson sampled. Blank spaces indicate parameter combinations resulting in numerical errors. Results are nearly identical to the deterministic model results in Fig. 2b, indicating stochastic effects are largely unnecessary to identify parameters consistent with the empirical distribution. **b)** Comparison of normalized empirical distribution and computationally modeled distribution, with and without stochastics, for the best-fit parameters (m=2, τ_ε=1.5, τ_κ=1.8). Blue lines represent 200 individual runs with stochastic fluctuations, as described in (a). The distribution is not substantially altered. **c)** Metric values comparing empirical and computationally modeled distributions run for 10^9 generations without progressive time rescaling. Blank spaces indicate parameter combinations that were not run due to computational limitations. Results are nearly identical to the progressive time rescaling shown in Fig. 2b, indicating the validity of the progressive rescaling procedure.

bioRxiv preprint doi: https://doi.org/10.1101/2025.01.09.631797; this version posted January 10, 2025. The copyright holder for this preprint (which was not certified by peer review) is the author/funder, who has granted bioRxiv a license to display the preprint in perpetuity. It is made available under aCC-BY 4.0 International license.

Supplementary Note SN1

Contents

1 Analytic modeling of the repeat length distribution in steady state 42
1.1 Summary of parameterized mutation rates at long lengths . . . 44

2 Finite difference equation for repeat length changes 45
2.1 Changes in repeat length due to expansion, contraction, and insertion . . . 45
2.2 Changes in repeat length due to substitutions . . . 46
2.3 Finite difference equation . . . 47

3 Steady-state dynamics in the short repeat length regime 47
3.1 Geometric solution to the substitution-only difference equation . . . 48
3.2 Interactions between short and long repeats are largely restricted to boundary effects . . . 49

4 Steady-state dynamics for asymptotically long repeat lengths 50
4.1 Local contributions to the change in P_L . . . 50
4.1.1 Competition between first-order local effects . . . 52
4.1.2 Second-order corrections to the local behavior and diffusive dynamics . . . 53
4.2 Repeat fission as a nonlocal contribution to changes in length . . . 54
4.2.1 Substitution-based fission . . . 54
4.2.2 Insertion-based fission . . . 55
4.3 Repeat fusion under random sampling of the length distribution . . . 55
4.4 Steady-state condition for long repeat dynamics . . . 56
4.5 Decomposition of parameter space into dynamical regimes . . . 58
4.5.1 Asymptotic length dependence of local transition rates and $\Delta\tau$ . . . 58
4.5.2 Relative strengths of substitution- and insertion-driven fission and L_{fis} . . . 59
4.5.3 Distinguishable dynamical regimes . . . 60
4.6 Unstable dynamics in the asymptotically expansion-biased regime $\Delta\tau \leq 0$ . . . 61
4.7 Stable dynamics in the asymptotically contraction-biased regime $\Delta\tau > 0$ . . . 62
4.7.1 Strong asymptotic contraction bias . . . 62
4.7.2 Strictly local approximation for strong asymptotic contraction bias $\Delta\tau \gg 1$ . . . 63
4.8 Intermediate asymptotic contraction bias . . . 63
4.9 Weak asymptotic contraction bias . . . 64
4.10 Obtaining numerical solutions to the steady state dynamics for $\Delta\tau < 0$ . . . 65

5 Comparison between numerical solutions and computationally modeled distributions 66
5.0.1 Comparisons of approximations to the dynamics and steady-state distributions . . . 66

6 Empirical constraints on parametric model 68
6.1 Constraints on $\Delta\tau$ and L^* . . . 68
6.2 Constraints on L_{fis} . . . 69

bioRxiv preprint doi: https://doi.org/10.1101/2025.01.09.631797; this version posted January 10, 2025. The copyright holder for this preprint (which was not certified by peer review) is the author/funder, who has granted bioRxiv a license to display the preprint in perpetuity. It is made available under aCC-BY 4.0 International license.

Supplementary Note Figures

Figure SN1 *Comparison between computational model results and numerical solutions to steady state equations for* $m = 8$ *for parameter values with of constant* $\tau_\epsilon + \tau_\kappa = 3$. 70
Figure SN2 *Computational model results for the net flux per mutation type.* 71
Figure SN3 *Computational model results for directional flux per mutation type* 72
Figure SN4 *Computational model results for collective non-normalized fluxes showing the relevance of local transitions, fission, and fusion.* 73
Figure SN5 *Comparison between computational model results and numerical solutions to steady state equations for* $m = 2$. 74
Figure SN6 *Comparison between computational model results and numerical solutions to steady state equations for* $m = 16$. 75
Figure SN7 *Comparison between computational model results and numerical solutions to steady state equations for* $m = 32$. 76
Figure SN8 *Accuracy of analytic approximation for distribution falloff in the* $\Delta\tau \gg 1$ *regime.* . . . 77

bioRxiv preprint doi: https://doi.org/10.1101/2025.01.09.631797; this version posted January 10, 2025. The copyright holder for this preprint (which was not certified by peer review) is the author/funder, who has granted bioRxiv a license to display the preprint in perpetuity. It is made available under aCC-BY 4.0 International license.

1 Analytic modeling of the repeat length distribution in steady state

To better understand our observations, we sought to describe the equilibrium that emerges once the repeat length distribution (herein referred to as P_L in discrete form and $\rho(L)$ under a continuum approximation) has reached steady state at asymptotically late times (i.e., $P(L;t) \to P_{ss}(L)$ as $t \to \infty$, where the subscript ss denotes steady state). Importantly, this does not occur for all parameter combinations, as a subset remain inherently unstable. For the parameter values that stabilize, the shape of the distribution is described by a dynamic balance that emerges between mutational processes, with distinct mutational effects dominating the dynamics in different length regimes. For simplicity, the analysis provided here is restricted to the case of mononucleotide repeats consisting of various numbers of A bases. Alternative bases that terminate each sequence, labeled B, represent any T, C, or G base (i.e., any non-motif base for A repeats) at either end of an A repeat and the explicit distribution of lengths of B strings are ignored for the present purposes.

As described in the manuscript, the mutational processes that are included in our model are the following, explicitly enumerated here with corresponding variables used to represent and parameterize the rates of each mutation type for clarity.

1. *Lengthening substitutions* μ: Point mutations $B \to A$ that increase the length of a repeat while preserving the total length of the genome. Such mutations only increase repeat length when they occur on bases adjacent to an existing repeat. Note that two additional transitions result from such mutations: fusion of nearby repeats (merging of two shorter repeats to form a longer repeat) and generation of new 'repeats' of length L=1 (included in our description for completeness, despite the lack of repetition). The distinction between these processes can be categorized by the number of B bases adjacent to the mutated site, one, zero, and two, for lengthening, fusion, and generation, respectively. The per-target rate mu is assumed to be the same constant for any repeat length L such that the per-repeat rate of these substitutions is necessarily linear in length, scaling as $\mu \times L$ for all repeat lengths.

2. *Shortening substitutions* ν: Point mutations $A \to B$ that decrease repeat length while preserving genome length. Such mutations also result in repeat fission (interruption of a repeat that forms two smaller repeats) and repeat destruction (mutation of L=1 repeats, removing them from the distribution). Again, these transitions can be categorized by adjacency of the mutated base to one, zero, and two B bases for repeat shortening, fission, and destruction, respectively. The per-repeat rate of shortening substitutions is again linear in repeat length: $\nu \times L$.

3. *Contractions* κ_L: Deletions of a single A base that decrease repeat length and genome length by one unit (i.e., one nucleotide for mononucleotide repeats). Extended deletions of two or more bases are assumed to be subdominant for simplicity (consistent with estimates provided in **Supplementary Figure S3**). Repeat contractions occur at a length-dependent rate constrained by empirical estimates and further parameterized by two variables; all rates for lengths $L \leq 8$ are fixed by trio-based rate estimates (see **Methods** and **Figure 2a**), the rate at $L = 9$ is determined by a constant multiple m relative to the value at $L = 8$ (i.e., $\kappa_{L=9} = m \times \kappa_{L=8}$), and rates for $L > 9$ follow a power law dependence of the form $\kappa_{L>9} = C_\kappa L^{\tau_\kappa}$. Here, the length-independent constant C_κ sets the initial value of the power law that parameterizes the contraction rate for all $L \geq 9$ (for intuition, if the power law $C_\kappa L^{\tau_\kappa}$ was artificially extended below $L = 9$, C_κ would be the rate associated with $L = 1$: $C_\kappa L^{\tau_\epsilon}|_{L\to 1} = C_\kappa$); the subscript c refers to the crossover in behavior between substitution-dominated mutation rates at lengths $L < 9$ and the onset of repeat instability-dominated rates for $L > 9$. Under our parameterization, solving for $\kappa_9 = C_\kappa L^{\tau_\kappa}|_{L=9} = m\ \kappa_8$ yields the definition $C_\kappa = \kappa_{L=8} \times m/9^{\tau_\kappa}$, which is dependent on both τ_κ and m, but constant for a fixed parameter combination $\{m, \tau_\epsilon, \tau_\kappa\}$. The per-repeat target size for contractions in a repeat of length L is simply L, as the deletion of any base in an A repeat has an equivalent effect. Thus, the per-repeat rates are given by multiplying κ_L by an additional factor of L (i.e., the per-repeat contraction rate becomes $\kappa_L L = C_\kappa L^{1+\tau_\kappa}$ for long repeats $L \geq 9$). For all length-dependent rates (e.g., κ_L), we use the subscript L to denote the length dependence for both a discrete list of rates (e.g, κ_L

bioRxiv preprint doi: https://doi.org/10.1101/2025.01.09.631797; this version posted January 10, 2025. The copyright holder for this preprint (which was not certified by peer review) is the author/funder, who has granted bioRxiv a license to display the preprint in perpetuity. It is made available under aCC-BY 4.0 International license.

defined in the discrete range of positive definite integers $L \in \mathbb{Z}^+$) and in the continuum where these rates are interpreted as continuous functions (e.g., $\kappa_L \to \kappa(L)$ becomes a continuous function describing the contraction rate in the continuous space $L \in \mathbb{R}^+$, but we preserve the notation $\kappa_L \equiv \kappa(L)$ in this case for consistency).

4 *Expansions* ϵ_L: Insertions of a single A base that increase repeat length and genome length by one unit. Again, extended insertions are assumed to occur at subdominant rates (see **Supplementary Figure S3**). Expansions are defined to be dependent on length in the same way as contractions, defining low lengths empirically and including the same value for the parameter m, but with a distinct power law for rates at large lengths: $\epsilon_{L>9} = C_\epsilon L^{\tau_\epsilon}$. We have again expressed this dependence using the length-independent constant $C_\epsilon = \epsilon_8 \times m/9^{\tau_\epsilon}$ for notational convenience. We envision insertion as the replacement of a single A base with a pair of bases AA such that the per-target lengths are again multiplied by the target size L (e.g., asymptotically, $\epsilon_L L = C_\epsilon L^{1+\tau_\epsilon}$).

5 *Non-motif insertions* ι_L: Insertions of a single B base that interrupts a repeat, resulting in repeat fission. For conciseness, non-motif insertions will simply be referred to as *insertions* herein (with the implicit distinction between insertions leading to expansions and non-motif insertions). Extended insertions of more than a single base are again ignored (see **Supplementary Figure S3**). The per-target rate of insertions is again length dependent in the same way, with $\iota_{L\leq 8}$ derived from empirical data, with the parameterization-dependent $\iota_9 = m\iota_8$ defined using the same multiple m, and rates for lengths $L > 9$ defined by the power law $\iota_L = C_\iota L^{\tau_\iota}$. In this case, the per-repeat rate is dependent on a target size of $L - 1$ (B must be inserted between two A bases in the pre-mutated repeat and there are $L - 1$ pairs of adjacent motifs providing targets for $AA \to ABA$). Herein, we will assume that the rate ι_L was estimated by collecting the average per-repeat rate of insertions and dividing by the length of the repeat L (i.e., as an effective per-target rate), as in **Methods**; however, if the per-target rate ι_L is estimated directly (i.e., by separately counting each $AA \to ABA$ event), one may resort to the approximation $L - 1 \approx L$ in the asymptotic large L regime, which is the only relevant length range for insertion-based processes due to their highly subdominant rates (relative to, e.g., the expansion or contraction rates in the same regime). We impose one additional assumption here to limit the number of free parameters in our model: with empirical motivation (see manuscript text), we impose the constraint $\tau_\iota = \tau_\epsilon$ under the assumption that they are both consequences of the same biological mechanisms such that the rates scale in parallel as L^{τ_ϵ}, albeit with non-motif insertions occurring at significantly lower rates. Thus, the per-repeat rate of insertions in our model is given by $\iota_L L = C_\iota L^{1+\tau_\iota} = C_\iota L^{1+\tau_\epsilon}$ (i.e., multiplying ι_L by L, rather than $L - 1$, to recover the estimated per-repeat rate). As before, this is expressed in terms of the length-independent constant $C_\iota = \iota_{L=8} \times m/9^{\tau_\epsilon}$, where we have substituted in $\tau_\iota = \tau_\epsilon$.

All additional mutational processes and their impact on the repeat length distribution are assumed to be subdominant or ignored for analytic simplicity. For example, deletions of B bases are ignored throughout our analysis despite potential relevance to fusion rates, as the direct estimates of the deletion rate for length one B strings showed an orders of magnitude suppression relative to the substitution rate μ (a directly competing process resulting in repeat fusion; see **Methods**). We make no attempt to model the correlated dynamics of the B string length distribution such that insertions of B bases that lengthen B strings are also ignored, along with the insertion or deletion of extended sequences (i.e., $L \to L \pm k$, where $k > 1$; see above). With the exception of extended insertions and deletions, these effects were included in the results of our computational model at appropriate length-independent rates (e.g., the rate of $B \to BB$ was assumed to be the same as the rate of $BB \to BBB$, ignoring potential repeat instability due to the indistinguishability between $B \in \{C, T, G\}$), but do not change the qualitative behavior of the repeat length distribution. Further, we assume the constant m is positive definite and the exponents τ_κ and τ_ϵ are positive semidefinite, which together imply that $C_\kappa, C_\epsilon, C_\iota > 0$.

Having defined each mutational process, we note that the location of the same type of mutation along the repeat sequence can result in dramatically different effects, as alluded to above. The catalogue of changes to repeat length can be separated into local transitions (i.e., increases or decreases in length by

bioRxiv preprint doi: https://doi.org/10.1101/2025.01.09.631797; this version posted January 10, 2025. The copyright holder for this preprint (which was not certified by peer review) is the author/funder, who has granted bioRxiv a license to display the preprint in perpetuity. It is made available under aCC-BY 4.0 International license.

one motif unit: $L \rightarrow L \pm 1$) and nonlocal transitions (i.e., $L \pm k$ where $k > 1$). While expansions are strictly local processes describing transitions in repeat length ($L \rightarrow L+1$) with probabilities associated with $P_L \rightarrow P_{L+1}$, μ substitutions are only local when they occur at sites adjacent to only one repeat boundary (e.g., $BBAB \rightarrow BAAB$, rather than $ABAB \rightarrow AAAB$). Importantly, μ substitutions adjacent to two distinct repeats result in an inherently nonlocal process in which two repeats (e.g., length L_1 and L_2) are fused into a single longer repeat of length L_1+L_2+1. This transition is both nonlocal in length space and non-conservative with respect to the mass of the probability distribution P(L). Transitions generating new $L = 1$ 'repeats' via substitution similarly do not conserve mass, effectively corresponding to a source at the $L = 1$ boundary. The latter is counteracted by a sink at the same boundary due to ν mutations (with a distinct rate) that remove $L = 1$ repeats. Analogously, repeat fusion is counteracted by repeat fission due to ν substitutions that occur in the non-boundary bases (i.e., in the 'body') of the repeat and are again nonlocal in length space and non-conservative in distribution mass; notably, this process has a target linear in the length of the original repeat (strictly, $L-2$, ignoring the boundaries), in contrast to the target for fusions, which is dependent on the number of B strings of length one). Local decreases in length can occur due to contraction (at a per-repeat rate *at least* linear in the length) or via ν substitutions (at a rate 2ν corresponding to the finite target size of two bases for repeats $L \geq 2$). Additional fission events can occur due to insertions, but, as noted above, additional fusion events due to deletions of B bases will be ignored throughout.

The nonlocal, non-conservative, and non-linear (e.g., power law dependencies and fusion rates dependent on two distinct length classes) nature of this ensemble of dynamics makes the difficulty of simultaneously modeling these effects immediately clear. Instead, motivated by the fundamental questions surrounding the maintenance and prevalence of long (and potentially disease-causing) repeats in the genome, we proceed with an analysis of the asymptotic dynamics. This approach provides a more straightforward understanding of the dominant processes appropriate in long repeat regime (i.e., $L \gg 1$), and how this contrasts from the forces shaping the short repeat end of the distribution (roughly defined here as $L < 10$). Due to its simplicity and an immediately recognizable shape, we first focused on the low L end of the distribution.

1.1 Summary of parameterized mutation rates at long lengths

For clarity, we summarize the mutation rate parameterization for each mutation type. Repeat lengths below $L = 9$ units are taken directly from empirical point estimates at each length (see **Figure 2a**) calculated for expansion, contraction, and insertions. Substitution rates μ and ν are taken from motif-dependent empirical estimates and assumed to be length independent. For repeat length $L \geq 9$, the per-target rates for expansion (ϵ_L), contraction (κ_L), and insertion (ι_L) are parameterized as follows.

$$\begin{aligned}
\epsilon_L &= C_\epsilon L^{\tau_\epsilon} \equiv \epsilon_8 m \left(\frac{L}{9}\right)^{\tau_\epsilon} \\
\kappa_L &= C_\kappa L^{\tau_\kappa} \equiv \kappa_8 m \left(\frac{L}{9}\right)^{\tau_\kappa} \\
\iota_L &= C_\iota L^{\tau_\epsilon} \equiv \iota_8 m \left(\frac{L}{9}\right)^{\tau_\epsilon}
\end{aligned} \tag{SN1}$$

The parameter-dependent constants C_ϵ, C_κ, and C_ι are computed from ϵ_8, κ_8, and ι_8, respectively, the empirical estimates at $L = 8$. The dependence on the number nine is a result of the largest length with reliable empirical estimates; for the purposes of the following analysis it is only important that this number is of order 10. Per-repeat rates are computed by multiplying each per-target rate by repeat length L (e.g., $\epsilon_L L$ for expansions, etc.). Noting that we have made the simplifying assumption that insertions and expansions obey the same power law (i.e., $\tau_\iota = \tau_\epsilon$), this provides a three-parameter description of the unobserved length dependencies of the mutation rates in terms of $\{m, \tau_\epsilon, \tau_\kappa\}$. This parameterization is used in our computational model to propagate the mutational process over time and substituted into more general analytic expressions below for direct comparison.

bioRxiv preprint doi: https://doi.org/10.1101/2025.01.09.631797; this version posted January 10, 2025. The copyright holder for this preprint (which was not certified by peer review) is the author/funder, who has granted bioRxiv a license to display the preprint in perpetuity. It is made available under aCC-BY 4.0 International license.

2 Finite difference equation for repeat length changes

We first modeled the dynamics of repeat length changes by writing a discretized finite difference equation that describes the combined effects of all five mutational processes on the distribution $\mathrm{P}_L(t)$ as it evolves over time.

$$\mathrm{P}_L(t+1) \approx \mathrm{P}_L(t) + \Delta_\epsilon \mathrm{P}_L(t) + \Delta_\kappa \mathrm{P}_L(t) + \Delta_\iota \mathrm{P}_L(t) + \Delta_\mu \mathrm{P}_L(t) + \Delta_\nu \mathrm{P}_L(t) \tag{SN2}$$

Here, we have assumed that all mutation rates are sufficiently small that the mutational processes can be linearized (i.e., the rate of multiple mutations on the same repeat in a single generation is negligibly small). This equation describes the discrete distribution at time $t+1$ as it evolves from the distribution at the previous time step due to changes induced by expansion, contraction, insertion, and substitutions (from both μ and ν mutations), respectively. This can be rearranged to express the change to the distribution for each length bin L in a single generation.

$$\Delta_t \mathrm{P}_L \approx (\Delta_\epsilon + \Delta_\kappa + \Delta_\iota + \Delta_\mu + \Delta_\nu)\mathrm{P}_L \tag{SN3}$$

Each term in this expression is defined by the single-generation effect of a given mutational type on the number of length L repeats P_L.

2.1 Changes in repeat length due to expansion, contraction, and insertion

Expansions introduce single-unit changes in length ($L \to L+1$), independent of their location within the repeat (target size L, as defined above). The change in the number of repeats in the Lth class can be written as a combination of an influx due to insertion from below ($L-1 \to L$) and an outflux due to expansion to the class above ($L \to L+1$), as follows.

$$\Delta_\epsilon \mathrm{P}_L = \epsilon_{L-1}(L-1)\mathrm{P}_{L-1} - \epsilon_L L \mathrm{P}_L \tag{SN4}$$

As defined above, ϵ_L represents the length-dependent expansion rate, which dramatically and monotonically increases above lengths of order ten. Contractions have the opposite effects: an influx from above ($L+1 \to L$) and an outflux to below ($L \to L-1$), with a length-dependent mutation rate κ_L and target size L.

$$\Delta_\kappa \mathrm{P}_L = \kappa_{L+1}(L+1)\mathrm{P}_{L+1} - \kappa_L L \mathrm{P}_L \tag{SN5}$$

Importantly, we have omitted the effect of contraction-based repeat fusion, under the assumption that this process is subdominant to substitution-based fusion at all repeat lengths (see description of substitution-based fusion, below). (Non-motif) insertions are described by more complicated transitions, due to the position-dependent effects on length. Additionally, insertions are non-conservative, replacing one repeat with two repeats of shorter length ($L \to k, L-k$, where $k \geq 1$). The outflux due to insertions occurs at a length-dependent rate ι_L with target size L, which is a sum over the L possible locations for the insertion that result in distinguishable transitions (up to the symmetry $k \leftrightarrow L-k$; e.g., transitions $5 \to 4, 2$ are equivalent to $5 \to 2, 4$, despite distinguishable mutational targets). Note that, for the purposes of writing the finite difference equation, one need not keep track of the eventual state(s) resulting from an outflux. A focal class L can gain counts due to insertions in any repeat longer than L: insertions that occur L units away from either repeat boundary result in an increase in P_L, such that each length class $l > L$ has 2 potential targets for transitions $l \to L, l-L$ (in the case where $l = 2L$, there is a single target, but two length L repeats are added to P_L). Together, the effects of insertion on a focal L class can be written as follows.

$$\Delta_\iota \mathrm{P}_L = -\iota_L L \mathrm{P}_L + 2 \sum_{l=L+1}^{\infty} \iota_l P_l \tag{SN6}$$

Here, we assume that P_L decays sufficiently rapidly as L increases such that $\sum_l \iota_l P_l$ remains finite and P_L is normalizable. This sum characterizes repeat fission due to insertions alone, which are inherently nonlocal and

bioRxiv preprint doi: https://doi.org/10.1101/2025.01.09.631797; this version posted January 10, 2025. The copyright holder for this preprint (which was not certified by peer review) is the author/funder, who has granted bioRxiv a license to display the preprint in perpetuity. It is made available under aCC-BY 4.0 International license.

non-conservative transitions. However, note that contributions from transitions $L+1 \to L$ are local (though non-conservative) effects included in the same contribution. In this sense, this sum can be considered a combination of local transitions $L+1 \to L$ (contributing $2\iota_{L+1}P_{L+1}$) and nonlocal transitions $l \geq L+2 \to L$ (contributing $2\sum_{l=L+2}^{\infty} \iota_l P_l$); this decomposition is relevant only when insertion rates are appreciable at long repeat length, which we approximate in the continuum limit (detailed below). Similarly, the target size L in the outflux in Equation SN6 is a sum of targets for local and nonlocal transitions (with targets 2 and $L-2$, respectively).

2.2 Changes in repeat length due to substitutions

Substitutions behave distinctly from the above transitions, though some aspects of the transitions they induce are analogous. For example, the outflux due to shortening substitutions is analogous to insertions with a target size L, but length-independent rate ν; this combines substitutions at the boundary that result in local transitions $L \to L-1$ (with target 2) and substitutions in the repeat body (target $L-2$) that result in repeat fission. The influx due to ν substitutions is also analogous to insertions, represented by a sum over contributions from fissions of repeats of length $l \geq L+2 \to L$ and local transitions $L+1 \to L$.

$$\Delta_\nu \mathrm{P}_L = -\nu L \mathrm{P}_L + 2\nu P_{L+1} + 2\nu \sum_{l=L+2}^{\infty} P_l = -\nu L \mathrm{P}_L + 2\nu \sum_{l=L+1}^{\infty} P_l \tag{SN7}$$

Again, we assume $\sum P_l$ rapidly converges to a finite value such that P_L is normalizable, though this condition is weaker than for insertions due to the (naively) monotonic increase in the per-target rate ι_L.

Length-increasing substitutions with length-independent rate μ per target result in an outflux restricted to sites adjacent to the repeat boundary (i.e., μ mutations $B \to A$ at either end of $BA...AB$). This includes local transitions when the mutated B is initially adjacent to less than two A bases (e.g., $BBA \to BAA$ for transitions $L \to L+1$ or $BBB \to BAB$ for transitions $L = 0 \to 1$) and nonlocal transitions when the mutated base results in repeat fusion ($ABA \to AAA$). Here, the total target size is 2 (i.e., not separated into local vs. nonlocal transitions) and the net outflux is independent of the resulting repeat length(s).

The influx due to μ substitutions has distinct contributions from local transitions ($L-1 \to L$) and nonlocal fusion of shorter repeats ($L_1, L_2 \to L$, where $L_1 + L_2 = L-1$). For the purposes of describing the discrete dynamics, we can introduce a time-dependent constant $p_F(t)$ that represents the steady-state probability that mutation of a B base terminating a repeat results in repeat fusion, rather than a local transition; $p_F(t)$ (henceforth, the time-dependence of p_F will be left implicit for brevity) is dependent on the genomic distribution of B string lengths at time t (i.e., the fraction of B strings of length one) or, alternatively, the probability that the three-unit context of the mutated B results in a substitution $ABA \to AAA$ (i.e., the probability that the mutated B is adjacent to two A repeats). The rate of substitution-based repeat fusions is therefore proportional to μp_F and the rate of local length-increasing substitutions is proportional to $\mu(1-p_F)$. The quantity p_F can be measured empirically at a given time (at any time in steady state) from the B string length distribution, computed from other genome-wide quantities like the length of the genome and total number of repeats, or computed theoretically in simple cases (see discussion below), but is ultimately unimportant to our analysis and results. The combined effects of μ mutations on repeats of length L can be characterized as follows.

$$\Delta_\mu \mathrm{P}_L = -2\mu \mathrm{P}_L + 2\mu(1-p_F)\mathrm{P}_{L-1} + \mu p_F \sum_{l=1}^{L-2} P_l P_{L-1-l} \tag{SN8}$$

The sum in the rightmost term is the discrete form of a convolution of the distribution P_L with itself, which has an intuitive interpretation. For integers distributed according to the discrete probability distribution P_L (assuming P_L is properly normalized), the probability of randomly sampling two integers $L_1, L_2 (< L-1)$ that sum to a fixed value $L-1$ is given by the convolution $\sum_{l=1}^{L-2} P_l P_{L-1-l}$; a substitution adds one unit to form a repeat of length L. Here, we have assumed that distinct classes of P_L remain uncorrelated in steady state for simplicity. This may be an oversimplification of the true steady state because fusion events may preferentially reverse repeat fission (i.e., fusion can re-form the original length of an interrupted repeat generated by fission;

bioRxiv preprint doi: https://doi.org/10.1101/2025.01.09.631797; this version posted January 10, 2025. The copyright holder for this preprint (which was not certified by peer review) is the author/funder, who has granted bioRxiv a license to display the preprint in perpetuity. It is made available under aCC-BY 4.0 International license.

the length-dependent per-target rate of insertion-based interruptions can result in correlation between length classes, further complicating the dynamics).

We note that there can be a similar contribution that originates from deletions of a B adjacent to two repeats (i.e., deletion-based repeat fusion). However, based on the estimated mutation rates (see Methods), background deletions are orders of magnitude less frequent than μ substitutions such that deletion-induced fusion transitions occur at negligible rates.

2.3 Finite difference equation

Summing the above contributions to $\Delta \mathrm{P}_L$ yields the full finite difference equation describing the change in each length class P_L subject to two-way substitutions, expansions, contractions, and insertions.

$$\Delta_t \mathrm{P}_L = -\left(\nu L + 2\mu\right)\mathrm{P}_L + 2\nu \sum_{l=L+1}^{\infty} P_l + 2\mu(1-p_F)\mathrm{P}_{L-1} + \mu p_F \sum_{l=1}^{L-2} P_l P_{L-1-l}$$
$$-\left(\epsilon_L + \kappa_L + \iota_L\right) L \mathrm{P}_L + \epsilon_{L-1}(L-1)\mathrm{P}_{L-1} + \kappa_{L+1}(L+1)\mathrm{P}_{L+1} + 2\sum_{l=L+1}^{\infty} \iota_l P_l \qquad \text{(SN9)}$$

For clarity, the first line of terms summarizes changes due to substitution and the second summarizes insertion- and deletion-based changes due to repeat instability. Inclusion of deletion-based fusion and/or correlations between length classes, both of which are treated as negligible, would add an additional convolution and/or a dependence on the covariance between length classes, respectively.

Guided by striking differences in the length-dependencies of substitutions and repeat instability rates (**Figure 2a**), further analyze the steady state dynamics by separating into distinct length regimes in which a subset of mutational processes dictate the vast majority of changes in length. Under this separation of length scales, the dynamics of short repeats (roughly, $L \leq 8$ for mononucleotide-A repeats) and long repeats (roughly $L > 10$) are largely dominated by distinct mutational forces: the distribution of short repeats is maintained in a dominant balance between the opposing effects of μ and ν substitutions (including both fission and fusion), while length changes to long repeats are dominated by a (parameter-dependent) balance between expansions, contractions, and fission (potentially including fission resulting from ν substitutions). The assumption of dominant balance allows for a dramatic simplification of the full set of contributions to Equation SN9; due to their low rates, all neglected terms provide minor corrections to the resulting approximation to the steady state distribution, which we demonstrate post-hoc.

Equation SN9 represents the deterministic change of the repeat length distribution in a reference sequence (i.e., a single individual) due to mutations alone. Here, we have assumed that natural selection is absent such that the accumulation of mutations in the reference results from mutations aggregating along the lineage ancestral to this individual. This process occurs over sufficiently long times that a steady state is eventually reached. Once in steady state, the time-averaged distribution of repeat lengths (i.e., averaging over stochastic behavior) can be equated to the steady state distribution obtained from Equation SN9.

3 Steady-state dynamics in the short repeat length regime

In contrast to previous studies, the empirical distribution we constructed includes very short sequences and, notably, single base sequences for comparison. The relative rates of expansions, contractions, and insertions to both types of substitutions makes it clear that repeat instability is are largely irrelevant to the maintenance of such short sequences (see **Figure 2a**). From a biological standpoint, this implies that repeat instability is technically irrelevant (i.e., occurs at highly suppressed rates) until repeats exceed roughly $L = 8$ (this differs slightly between motifs of distinct lengths and is more accurately described as roughly 8-10 nucleotides, rather than repeat units, for motifs of length $l_m \leq 4$ nucleotides; see **Supplementary Figure S2**,); this corresponds to the length range where expansion and contraction rates are comparable to substitution rates such that repeat instability becomes relevant to the dynamics.

bioRxiv preprint doi: https://doi.org/10.1101/2025.01.09.631797; this version posted January 10, 2025. The copyright holder for this preprint (which was not certified by peer review) is the author/funder, who has granted bioRxiv a license to display the preprint in perpetuity. It is made available under aCC-BY 4.0 International license.

The shape the distribution of short repeats is well-approximated as a balance between substitutions alone. The distribution is generated by the random process of sequence evolution under two-way substitution with distinct rates μ and ν. Given indefinite time, this process equilibrates to a steady state genome for which the probability of randomly sampling an A base is given by the fraction $p_A = \mu/(\mu+\nu)$ and the complementary probability of sampling a B base is given by $p_B = 1 - p_A = \nu/(\mu+\nu)$. Here, the relevant substitution rates μ and ν are the single-unit context rates (i.e., averaged over longer contexts) $\mu_{B\to A}$ and $\mu_{B\to A}$, respectively, as this distribution is not characterized by distinctions between local transitions, fission, and fusion. Under this model, a repeat is simply a contiguous sequence of A bases L bases long adjacent to a B base on either side. Conditioning on an initial B base, a length L string of A bases occurs at a frequency approximately given by the probability of randomly sampling an A base L successive times, followed by a terminating B base. The frequency of a length L repeat is therefore given by geometrically distributed distribution proportional to ${p_A}^L p_B$.

$$\mathrm{P}_L = \mathcal{N}\left(\frac{\mu}{\mu+\nu}\right)^L\left(\frac{\nu}{\mu+\nu}\right) \propto \left(\frac{\mu}{\mu+\nu}\right)^L \tag{SN10}$$

Here, $\mathcal{N}$ is a normalization constant defined as $\mathcal{N} = 1/\sum_{L=1}^{\infty}\mathrm{P}_L$, where $\sum_L \mathrm{P}_L$ is the total mass of the distribution. As the steady state distribution is no longer geometrically distributed when repeat instability becomes relevant roughly above $L = 8$, the value of $\mathcal{N}$ cannot be determined by the short repeat dynamics alone and must be evaluated only after identifying the steady state distribution over all length classes. Given that the constant $\mathcal{N}$ is unknown, the constant probability associated with sampling repeat-terminating B bases can be absorbed into the definition of $\mathcal{N}$.

3.1 Geometric solution to the substitution-only difference equation

To demonstrate the utility of the finite difference equation in a simpler setting, one can show that the geometric distribution, when normalized, provides the solution to the short length approximation to Equation SN9 in steady state. Under substitutions alone, the short length regime is well-approximated by the following difference equation.

$$\Delta\mathrm{P}_L \approx -\nu L\mathrm{P}_L - 2\mu\mathrm{P}_L + 2\nu\sum_{k=L+1}^{\infty} P_k + 2\mu(1-p_F)P_{L-1} + \mu p_F \sum_{k=1}^{L-2} P_k P_{L-1-k} = 0 \tag{SN11}$$

The finite difference equation becomes a steady state condition by imposing time independence (i.e., $\Delta\mathrm{P}_L = 0$). For consistency with Equations SN7 and SN8, the local influx from ν substitutions is included in the sum representing fission and the parameter $p_F = (1-q_F)$ will be defined below to represent the appropriate rate of fusion in the present context. The normalized geometric distribution is given by the following expression in terms of p_A and $p_B = 1 - p_A$, defined above.

$$\mathrm{P}_L = p_B {p_A}^{L-1} = \frac{\nu\mu^{L-1}}{(\mu+\nu)^L} \tag{SN12}$$

Though fusion and fission are inherently non-conservative transitions that change the number of repeats in the distribution, we make the approximation that individual fission and fusion events do not significantly alter the normalization constant in steady state. The term representing fission is now the incomplete sum over a geometric series, which can be explicitly evaluated as follows.

$$2\nu\sum_{k=L+1}^{\infty} P_k = 2\nu p_B \sum_{k=L+1}^{\infty} {p_A}^{L-1} = 2\nu p_B \frac{{p_A}^L}{p_B} = 2\mu\mathrm{P}_L \tag{SN13}$$

Thus, the influx due to both repeat fission and local transitions, both ν substitution-based effects, exactly cancel the outlux due to μ mutations.

To evaluate the convolution term, note that the probability distribution for the length of B strings under two-way substitution $\tilde{P}_\lambda$ is geometric by the same arguments as for the A distribution, but with reversed

bioRxiv preprint doi: https://doi.org/10.1101/2025.01.09.631797; this version posted January 10, 2025. The copyright holder for this preprint (which was not certified by peer review) is the author/funder, who has granted bioRxiv a license to display the preprint in perpetuity. It is made available under aCC-BY 4.0 International license.

probabilities $p_A \leftrightarrow p_B$. Normalizing string lengths $\lambda = [1, \infty)$ (i.e., conditional on a length $\lambda \geq 0$ B string), the normalized probability distribution is as follows.

$$\tilde{P}_\lambda = p_A {p_B}^{\lambda-1} \tag{SN14}$$

The probability of the B string that terminates any given A string being length $\lambda = 1$ is simply $\tilde{P}_1 = p_A$. This is also the steady state probability of fusion p_F, for which a μ substitution in a length one B string results in the transition $L_1, L_2, \rightarrow L_1 + L_2 + 1$. The complement is the probability of locally increasing the length by a single unit (as opposed to fusion) due to an adjacent μ substitution, $q_F = 1 - \tilde{P}_1 = 1 - p_A = p_B$. After cancelling the ν substitution influx with the μ substitution outflux, we can rewrite the remaining terms in Equation SN11 by substituting in $p_F = p_A$, $q_F = p_B$ and evaluating the partial sum in the fusion term.

$$\begin{aligned} -\nu L\mathrm{P}_L + 2\mu q_F P_{L-1} + \mu p_F \sum_{k=1}^{L-2} P_k P_{L-1-k} &= -\nu L\mathrm{P}_L + 2\mu p_B P_{L-1} + \mu p_A {p_B}^2 \sum_{k=1}^{L-2} {p_A}^{k-1} {p_A}^{L-k-2} \\ &= -\nu L\mathrm{P}_L + 2\mu \frac{p_B}{p_A} \mathrm{P}_L + \mu p_A {p_B}^2 \sum_{k=1}^{L-2} {p_A}^{L-2} \\ &= -\nu L\mathrm{P}_L + 2\nu \mathrm{P}_L + \nu(L-2) p_B p_A {p_A}^{L-2} \\ &= -\nu(L-2)\mathrm{P}_L + \mu(L-2)\mathrm{P}_L = 0 \end{aligned} \tag{SN15}$$

We find that a geometric distribution of both A and B repeats satisfies the steady state equation under two-way substitution, which justifies our aforementioned approximation for the substitution-dominated short length regime of the distribution.

3.2 Interactions between short and long repeats are largely restricted to boundary effects

For the present purposes, we are only concerned with the geometric falloff in Equation SN10 that defines the shape of the distribution in this length regime, which is given by the proportionality on the right hand side of the above expression. Importantly, this length dependence remains largely independent of the dynamics of the long length regime, which only affects the normalization constant. This separation of the dynamics for short and long length repeats is a reasonable approximation because the rate of transitions between these regimes is low and primarily limited to the intermediate lengths at $L \sim 8-10$ (i.e., the boundary between the short and long repeat regimes). As discussed in the context of the long length dynamics described below, we allow for an influx from the long length regime because it is inherently negligible due to the relative mass of short length repeats vastly outweighing the mass in the long repeat tail of the distribution. In this sense, the short repeat regime can be treated as a probability sink for the long repeat distribution for length transitions that exit the long length regime. Similarly, the mass in the long length regime is sourced by short repeats that approach the length regime boundary and are quickly subject to expansion-biased instability; this can be considered a local source at the boundary between the regimes without altering the dynamics elsewhere. If the steady state is in detailed balance (i.e., the flux in and out of each length class vanishes independently), fluxes at the length regime boundary must cancel such that both ends of the distribution maintain their relative weights.

While it is immediately clear that the distribution of A repeats deviates from a simple geometric decay due to the combined action of expansion, contraction, and insertion in the long length regime, the B string length distribution is unaltered by expansion and contraction, which solely manipulate A repeat length. While likely unrealistic, we proceeded under the assumption that B strings do not constitute repeats and are therefore not directly subject to repeat instability. Therefore, any deviation from a simple geometric distribution can only be due to the effects of insertion, which generates new length one B strings during each transition. Although the additional source of B strings will contribute to the total number of B strings in the eventual steady state balance between the A and B distributions, localization of this influx to the lowest length class implies that the normalized distribution of B lengths remains approximately geometric.

bioRxiv preprint doi: https://doi.org/10.1101/2025.01.09.631797; this version posted January 10, 2025. The copyright holder for this preprint (which was not certified by peer review) is the author/funder, who has granted bioRxiv a license to display the preprint in perpetuity. It is made available under aCC-BY 4.0 International license.

4 Steady-state dynamics for asymptotically long repeat lengths

The dynamics the steady state distribution at large lengths is qualitatively distinct in shape from the low length regime, with a much heavier tail than the geometric distribution that decays more slowly with length. Although one must speculate about the behavior in a length regime without direct estimates from de novo data, the assumption that repeat instability is associated with a (naively monotonic) length-dependent increase in the per-target expansion and contraction rates implies that at some length these mutational processes dominate over length-independent per-target substitution rates; this relative increase in rates can already be seen for lengths of order 10 (see **Figure 2a**). Consequently, these rates necessarily continue to increase in excess of the substitution rate, eventually dominating the mutational dynamics. However, the point at which substitution-based fission, which scales linearly with repeat length, can be ignored is entirely parameter dependent. As a result, we can infer that the forces relevant to the longest length repeats present in the distribution are some subset of expansion, contraction, substitution-based fission, and insertion-based fission (or the combination of all four). To analyze the dynamics at long repeat length and characterize the relative importance of each mutational process, we took the large length continuum limit of the full finite difference equation under the approximation that, for long repeats, $L \gg 1$ such that the distance between length bins can be treated as infinitesimal. While the continuum limit may be appropriate above lengths of order 10, this description remains an approximation to the discrete dynamics detailed in Equation SN9. In the continuum, the distinction between local and nonlocal terms becomes important, as local differences are approximated by continuous derivatives, while the nonlocal sums in Equation SN9 that capture transitions from across the rest of the distribution are approximated as integral dependencies.

4.1 Local contributions to the change in P_L

We first focus on local changes to the length distribution due to each of the forces. Starting with expansion in Equation SN4, the discrete change to a focal length class P_L is the difference between an influx due to $L-1 \to L$ length-increasing transitions and an outflux due to $L \to L+1$ length-increasing transitions. All effects of expansion are entirely local, which allows for a local approximation to $\Delta_\epsilon \mathrm{P}_L$ using a Taylor expansion in the continuum. To elucidate the continuum limit for large repeat length, we treat expansions in detail, below, but note that the continuum approximation for all local contributions can be obtained analogously. We first define the distance between discrete length classes $\Delta L = 1$, which suggests that we must change units before formally taking the continuum limit and assessing the accuracy of the resulting approximation.

$$\begin{aligned} \Delta_\epsilon \mathrm{P}_L &= (L-1)\epsilon_{L-1}\mathrm{P}_{L-1} - L\epsilon_L \mathrm{P}_L \\ &= F_\epsilon(L-\Delta L) - F_\epsilon(L) \\ &= F_\epsilon(x-\Delta x) - F_\epsilon(x) \end{aligned} \quad \text{(SN16)}$$

Here, ϵ_L is the length-dependence of the expansion rate (e.g., $\epsilon_L = C_\epsilon L^{\tau_\epsilon}$ under our parameterization for $L \geq 9$). The function $f_\epsilon(L) = \epsilon_L L P_L$ was defined solely for notational convenience. In the third line, repeat length L was rescaled under a change of variables defined by $x = L/c$ (with $\Delta x = \Delta L/c = 1/c$); c is an arbitrary dimensionful constant with the same units as L (i.e., measured in a number of motif units) such that x is dimensionless. If x is kept fixed while L grows large, Δx gets increasingly smaller. Thus, choosing an appropriate c allows the backward finite difference $\Delta F_\epsilon(x) = F_\epsilon(x-\Delta x) - F_\epsilon(x)$ to be approximated by a Taylor expansion in small Δx truncated at a some finite order (for all practical purposes, this is equivalent to a Taylor expansion around "small" ΔL after setting $\Delta L = 1$ when $L \gg 1$, but side steps the concern that $\Delta L = 1$ motif unit is definitionally not infinitesimal). Taking the continuum limit $L \to \infty$ and $\Delta x \to 0$, F_ϵ becomes the continuous function f_ϵ and the discrete derivative approaches the continuous derivative $\Delta F_\epsilon \to \partial_x f_\epsilon$. For finite L (and thus finite c), the first-order continuum approximation is accurate

bioRxiv preprint doi: https://doi.org/10.1101/2025.01.09.631797; this version posted January 10, 2025. The copyright holder for this preprint (which was not certified by peer review) is the author/funder, who has granted bioRxiv a license to display the preprint in perpetuity. It is made available under aCC-BY 4.0 International license.

to order Δx^2.

$$\Delta_\epsilon \mathrm{P}_L = \frac{\partial x}{\partial L}\frac{\partial f_\epsilon(x)}{\partial x} + \mathcal{O}(\Delta x^2) \approx -\partial_L\left[\epsilon_L L \rho(L)\right] \tag{SN17}$$

This is expressed in terms of a general, length-dependent expansion rate ϵ_L (at sufficiently long lengths) to allow for generic parameterizations, including Equation SN1. To arrive at this approximation, the variable change was inverted (i.e., imposing $L = cx$, where $\partial L/\partial x = 1/c$; the chain rule is explicit in the middle expression for clarity) after taking the continuum approximation. Here, the notation ∂_x^n represents the nth derivative with respect to x and will be used henceforth for brevity. In the length continuum, we assume that the discrete distribution P_L can be well-approximated by the continuous, differentiable function $\rho(L)$ (defined in the rescaled length units $\rho(x) \equiv \lim_{\Delta x \to 0} P(x)$) such that the derivative $\partial_L \rho(L)$ represents the flux through length L. The continuum approximation is only applicable for $L \gg 1$ and all expressions dependent on $\rho(L)$ should be considered implicitly conditioned on $L \gtrsim 10$ such that the long-length scaling behavior of the mutation rates can be used directly (i.e., empirically-derived rates for $L < 9$ can be ignored such that length scaling for each rate is dictated by the parameterization, e.g., Equation SN1). Importantly, at finite L, corrections to the lowest-order approximation to the finite difference $\Delta F(L)$ for a discrete function $F(L)$ cannot be treated as negligible when $\partial_L f(L)$ vanishes (see discussion of second-order corrections, below). Equation SN17 describes a strictly local transition that drives a length-dependent flux through the focal length L, which represents the collective impact of expansion rates on the distribution; if desired, the chain rule can be applied to show a separate flux and length-dependent loss of mass due to expansion (i.e., $\Delta_\epsilon \mathrm{P}_L \approx -\rho(L)\partial_L(\epsilon_L L) - \epsilon_L L \partial_L \rho(L)$). Noting that the derivative is negative throughout the roughly monotonic empirical distribution, the flux due to expansion may describe an overall increase or decrease in length, depending on the relative magnitude of the non-derivative contribution.

The effects of contraction $\Delta_\kappa \mathrm{P}_L$ can be summarized analogously in terms of contributions from and to the adjacent longer and shorter length bins, respectively, which becomes a local length-dependent flux in the opposite direction in the continuum limit. This sign difference emerges because the continuum approximation is to a forward finite difference for contractions and to a backward finite difference for expansions (i.e., the difference between continuum approximations for $L+1 \to L$ and $L-1 \to L$ transitions).

$$\Delta_\kappa \mathrm{P}_L = (L+1)\kappa_{L+1}\mathrm{P}_{L+1} - L\kappa_L \mathrm{P}_L \approx \partial_L\left(\kappa_L L \mathrm{P}_L\right) \tag{SN18}$$

Like expansions, contractions amount only to local changes to the distribution, but the length dependence of the per-repeat rate remains important to our understanding of the dynamics.

In contrast, substitutions induce both local and nonlocal transitions. We first isolate the local transitions from in Equation SN7 and SN8, treating the nonlocal contributions as fissions and fusions (see below). In discrete form, the local influx and outflux towards increasing length due to μ substitutions and towards decreasing length due to ν substitutions can be represented as follows.

$$(\Delta_\mu + \Delta_\nu)_{local}\,\mathrm{P}_L = 2\mu(1-p_F)\left(\mathrm{P}_{L-1} - \mathrm{P}_L\right) + 2\nu\left(\mathrm{P}_{L+1} - \mathrm{P}_L\right) \tag{SN19}$$

Unlike expansion and contraction, the target size for local changes in length due to substitution is a length-independent factor of 2 associated with mutations at either boundary (e.g., $AAA \to AAB$ and $\to BAA$ for ν substitutions; μ substitutions reverse these transitions), which have a distinct effect from mutations at non-boundary loci. The target size for μ substitutions is reduced further because a fraction p_F transitions on the boundary result in fusion, rather than local increases in length (i.e., leaving a target of $2(1-p_F) = 2q_F$ per repeat). The first-order continuum approximation to Equation SN19 is the following.

$$(\Delta_\mu + \Delta_\nu)_{local}\,\mathrm{P}_L \approx -\partial_L\left(2\left(\mu(1-p_F) - \nu\right)\rho(L)\right) \tag{SN20}$$

Insertions include a similar local term, but this comes with an additional complication. If an insertion occurs adjacent to either boundary (with target size 2: $AA...AA \to ABA...AA$ and $\to AA...ABA$), the resulting repeat fission desribes the replacement of one length L repeat with one length $L-1$ repeat and

bioRxiv preprint doi: https://doi.org/10.1101/2025.01.09.631797; this version posted January 10, 2025. The copyright holder for this preprint (which was not certified by peer review) is the author/funder, who has granted bioRxiv a license to display the preprint in perpetuity. It is made available under aCC-BY 4.0 International license.

one length 1 'repeat' ($L \to L-1, 1$). This is the only example of repeat fission that also contains a local transition, but will not be explicitly accounted for in our treatment of insertion-based fission below. The local component of this contribution can be written as follows.

$$(\Delta_\iota)_{local} \mathrm{P}_L = 2\iota_{L+1}\mathrm{P}_{L+1} - 2\iota_L \mathrm{P}_L \approx \partial_L \left(2\iota_L \rho(L)\right) \tag{SN21}$$

Due to the limited target of two possible insertions resulting in this local transition, the appropriate length dependence inside the derivative is twice the per-target rate ι_L, rather than a dependence on the per-repeat insertion rate. The correlated nonlocal contribution to the $L = 1$ bin can be ignored because the relatively low rate of insertions results in an exceedingly small influx into $L = 1$ relative to the geometrically distributed mass maintained by substitutions.

4.1.1 Competition between first-order local effects

The length-independent (and modest) target size, matching power law exponent, and globally suppressed rate of insertions relative to expansions together ensure that local insertions remain subdominant to expansions (i.e., $(\Delta_\iota)_{local} \mathrm{P}_L \ll \Delta_\epsilon \mathrm{P}_L$ for any L); as a result, local insertions may be neglected entirely. By the same argument, and given that expansion and contraction rates far exceed the substitution rates at long lengths, local changes in length due to substitutions are likely negligible in the $L \gg 1$ regime of interest. Additionally, we will at this point treat time as continuous (i.e., assuming infinitesimal generation time after appropriately rescaling the units of all rates) such that the per-generation change in occupancy of P_L can be approximated by the time-derivative $\partial_t \rho(L)$ (note that we revert back to the notation $\Delta \mathrm{P}_L$ when referencing the discrete equations). Under these approximations, the first-order approximation to the local dynamics can be summarized by the following.

$$\begin{aligned}(\partial_t \rho(L))_{local} &\approx -\partial_L \left[\left((\epsilon_L - \kappa_L)L - 2\iota_L + 2(\mu(1-p_F) - \nu)\right)\rho(L)\right] \\ &\approx -\partial_L \left[\left((\epsilon_L - \kappa_L)L\right)\rho(L)\right]\end{aligned} \tag{SN22}$$

The quantity $\epsilon_L - \kappa_L$ represents the bias between expansion and contraction at a given length, indicating that the lowest-order approximation to the local dynamics describes a flux through each length class due to the length-dependent expansion-contraction bias.

The sign difference between the expansion and contraction terms suggests that, if their magnitudes are nearly identical at a given length (i.e., $(\Delta_\epsilon + \Delta_\kappa)P_l \approx 0$ such that length changes are symmetric at length l), otherwise subdominant contributions from insertion and/or substitution could become relevant. Under the somewhat simplistic parameterization in Equation SN1, this can occur only at a specific (but parameter-dependent) length L^*.

$$L^* \equiv \left(\frac{C_\epsilon}{C_\kappa}\right)^{\frac{1}{\Delta\tau}} = 9\left(\frac{\epsilon_8}{\kappa_8}\right)^{\frac{1}{\Delta\tau}} \tag{SN23}$$

We have expressed this in terms of the collapsed parameter $\Delta\tau$, the difference between the contraction and expansion exponents.

$$\Delta\tau \equiv \tau_\kappa - \tau_\epsilon \tag{SN24}$$

L^* is independent of the multiplier m and dependent only on $\Delta\tau$, rather than τ_ϵ and τ_κ individually. This length is notable because subdominant local contributions (i.e., from insertions and substitutions) could theoretically become relevant to the maintenance of an eventual steady state at specific lengths. However, second-order corrections (due to expansion and contraction) to the finite difference equation dramatically exceed the local effects of insertions and substitutions due to their scaling with target size.

bioRxiv preprint doi: https://doi.org/10.1101/2025.01.09.631797; this version posted January 10, 2025. The copyright holder for this preprint (which was not certified by peer review) is the author/funder, who has granted bioRxiv a license to display the preprint in perpetuity. It is made available under aCC-BY 4.0 International license.

4.1.2 Second-order corrections to the local behavior and diffusive dynamics

When the first-order approximation to the local dynamics vanishes (due to nearly symmetric expansion an contraction rates across some range of lengths), subsequent corrections to the continuum approximation at finite L can dominate the leading-order local behavior. The space of allowed parameter values allows L^* to sit within the populated range of long lengths such that a description of the dynamics across the full set of $\{m, \tau_\epsilon, \tau_\kappa\}$ parameter values requires further approximation of local length changes. For parameters consistent with the empirical distribution, L^* typically sits within the well-occupied range of the long-length tail due to modest values of $\Delta\tau$; for comparatively small $\Delta\tau$, the length scalings for expansion and contraction are similar such that the neighborhood of L^* (i.e., the range of lengths with small expansion-contraction bias) extends to a wider range of lengths. In this sense, both the location and neighborhood of L^* are dictated by $\Delta\tau$ and together indicate expansion-contraction bias alone is insufficient to describe local length changes observed in humans.

With this in mind, we obtained the next-order approximation (i.e., the first strictly non-vanishing contribution) to Equation SN9 by Taylor expanding the expressions for expansion, contraction, insertion, and substitution to second order in the continuum limit. For expansion, we approximate Equation SN17 to order Δx^2 in the dimensionless variable x (i.e., the appropriately rescaled L) to produce the following approximation.

$$\begin{aligned}\Delta_\epsilon \mathrm{P}_L &= \left[f_\epsilon(x) - \left[\partial_x f_\epsilon(x)\right]\Delta x + \frac{1}{2}\left[\partial_x^2 f_\epsilon(x)\right]\Delta x^2 + \mathcal{O}(\Delta x^3)\right] - f_\epsilon(x) \\ &\approx -\partial_L\left[\epsilon_L \rho(L)\right] + \frac{1}{2}\partial_L^2\left[\epsilon_L \rho(L)\right] \end{aligned} \quad \text{(SN25)}$$

All other mutational effects can be expanded to the same order analogously,. Importantly, the second-order contribution is strictly positive in each case such that their sum is strictly non-vanishing (in contrast to the sign difference that allows the first-order term to vanish at L^*). This results in the following approximation to the continuous-time local dynamics in the large length regime.

$$\begin{aligned}\left(\partial_t \rho(L)\right)_{local} \approx &-\partial_L\left[\left((\epsilon_L - \kappa_L)L - 2\iota_L + 2\left(\mu(1-p_F) - \nu\right)\right)\rho(L)\right] \\ &+ \frac{1}{2}\partial_L^2\left[\left((\epsilon_L + \kappa_L)L + 2\iota_L + 2(\mu(1-p_F) + \nu)\right)\rho(L)\right] \end{aligned} \quad \text{(SN26)}$$

The above (second-order) approximation in the continuum limit ($L \gg 1$) expresses the sum of local length changes for arbitrary length-dependent rates of expansion, contraction, and insertion, either estimated directly, or parameterized; higher order approximations would only become necessary in the neighborhood of a length at which the first and second derivative terms both vanish (for example, in the unrealistic scenario where inflection points in the length dependencies of $L\epsilon_L\rho(L)$, $L\kappa_L\rho(L)$, $\iota_L\rho(L)$, and $\rho(L)$ occur at the same length). Introducing the definitions of ϵ_L, κ_L, and ι_L under the power law parameterization allows for comparison between these length dependencies (along with the relative magnitudes of their local contributions).

$$\begin{aligned}\left(\partial_t \rho(L)\right)_{local} \approx &-\partial_L\left[\left(C_\epsilon L^{1+\tau_\epsilon} - C_\kappa L^{1+\tau_\kappa} - 2C_\iota L^{\tau_\epsilon} + 2\left(\mu(1-p_F) - \nu\right)\right)\rho(L)\right] \\ &+ \frac{1}{2}\partial_L^2\left[\left(C_\epsilon L^{1+\tau_\epsilon} + C_\kappa L^{1+\tau_\kappa} + 2C_\iota L^{\tau_\epsilon} + 2(\mu(1-p_F) + \nu)\right)\rho(L)\right] \end{aligned} \quad \text{(SN27)}$$

The second derivative terms in this approximation can be collectively viewed as describing diffusion-like changes to repeat length. Unlike the bias-driven fluxes represented by the first derivative term, the second-order term generates symmetric, bidirectional transitions (i.e., equiprobable local increases and decreases in length).

Interpreting the local dynamics, repeat instability drives a monotonic increase in the rate of expansion that results in a flux towards higher lengths represented by the first order term in Equation SN25; similarly, the second order term represents an increasing rate of diffusion with increasing length. Unlike the competing directional effects at first order, the strictly positive second-order contributions are net additive (i.e., both

bioRxiv preprint doi: https://doi.org/10.1101/2025.01.09.631797; this version posted January 10, 2025. The copyright holder for this preprint (which was not certified by peer review) is the author/funder, who has granted bioRxiv a license to display the preprint in perpetuity. It is made available under aCC-BY 4.0 International license.

expansion and contraction diffuse bidirectionally). Expansion-driven diffusion compounds with contraction-driven diffusion such that the length of longer repeats is increasingly unstable, including in the neighborhood of L^*. While the mutational forces do not counteract in this context, their relative rates can be compared to determine the dominant mutational processes underlying length diffusion. As with the first derivative, the minimal target sizes for local substitutions are dramatically outcompeted by expansion and contraction; length-independent substitution rates generate a uniform rate of diffusion with minimal magnitude. Despite the length-dependent per-target rate of insertion, the minimal target size and a lower initial rate (i.e.,. $C_\iota \ll C_\epsilon$ such that $\iota_L \ll \epsilon_L$, independent of parameter values) results in entirely negligible contributions to length diffusion relative to expansion. Given the negligible effects of both substitution and insertion, independent of parameter values, the local dynamics are well approximated by the following expression only dependent on expansion and contraction.

$$(\partial_t \rho(L))_{local} \approx -\partial_L \left[(\epsilon_L - \kappa_L) L\rho(L) \right] + \frac{1}{2}\partial_L^2 \left[(\epsilon_L + \kappa_L) L\rho(L) \right] \tag{SN28}$$

This general expression can again be evaluated under any parameterization of interest, including that of Equation SN1, provided our assumptions about the subdominant length-dependent rates of insertions remain appropriate. Local changes in length are thus a competition between bias-driven directional flux and diffusive changes in length. Clearly the latter dominates in the neighborhood of L^*, but the relative importance of these effects is also parameter dependent, comparing the net expansion-contraction bias (i.e., the asymmetric component of repeat instability) to the average magnitude of expansion and contraction (the symmetric component).

4.2 Repeat fission as a nonlocal contribution to changes in length

In contrast to the local effects described above, repeat fission and fusion are inherently nonlocal processes. Fission is a consequence of interruptions due to insertions or ν substitutions that generate both a flux out of the focal L class (by producing two shorter repeats) and a corresponding flux into the same class from fissions of longer repeats; the total number of perfect repeats is altered by replacing one longer repeat with two shorter repeats. Conceptual distinctions between substitution and insertion in this context are minor in the continuum: insertions allow transitions to and from adjacent length classes, while substitutions require transitions to and from at least two length classes away. Additionally, insertion within a repeat necessarily results in fission and does not conserve total genomic length (though, this does not *a priori* alter the mass of the A repeat distribution), unlike substitutions. The most pertinent difference owes to the length-dependent per-target rate of insertions, which can dramatically increase the number of such events as repeat length increases. In both cases, the nonlocal nature of fission-related transitions makes modeling the dynamics inherently more complex, as the influx from all bins of higher length amounts to a sum of distinct contributions to the change in P_L at each length (see Equations SN6 and SN7).

4.2.1 Substitution-based fission

Fission occurs only as a consequence of ν substitutions, with no dependence on the substitution rate μ, which only increase repeat length. After removing the target for local transitions via substitution at either repeat boundary (see Equation SN19), the remaining target for nonlocal transitions is $L-2$, corresponding to the body of the repeat. While the local transitions generate the derivatives shown in Equation SN26, the $L \gg 1$ continuum limit of the remaining terms in Equation SN7 results in an integral over the distribution of repeats longer than the focal class P_L.

$$\begin{aligned}(\Delta_\nu \mathrm{P}_L)_{fiss} &= -\nu(L-2)\mathrm{P}_L + \sum_{l=L+2}^{\infty} 2\nu P_l \\ &\approx -\nu(L-2\Delta L)\rho(L) + 2\nu \int_{L+2\Delta L}^{\infty} \rho(\lambda)d\lambda \\ &\approx -\nu L\rho(L) + 2\nu \int_{L}^{\infty} \rho(\lambda)d\lambda \end{aligned} \tag{SN29}$$

bioRxiv preprint doi: https://doi.org/10.1101/2025.01.09.631797; this version posted January 10, 2025. The copyright holder for this preprint (which was not certified by peer review) is the author/funder, who has granted bioRxiv a license to display the preprint in perpetuity. It is made available under aCC-BY 4.0 International license.

Here, the lower limit of the sum differs from Equation SN7 after removing local transitions $L+1 \to L$. The continuum approximation applies only in the asymptotically long length regime where $L \gg \Delta L = 1$, justifying the approximations $L + 2\Delta L \approx L$ (in the integration limit) and $L - 2 \approx L$ (in the target size dependence of the outflux term) in the final expression, above. The linear length scaling of the outflux characterizes the fact that repeat length changes due to any substitution in the repeat body. In contrast, there are exactly two targets for fission transitions from any given longer length class (i.e., specific substitutions L units away from either boundary of a length λ repeat result in a shorter repeat of length L; in the special case where $\lambda = 2L + 1$, only a substitution in the middle base is relevant, but yields two length L repeats). All longer repeats therefore contribute identically to the influx, independent of length. The net effect of fission depends on the focal length and many repeats of longer length sit in the tail of the distribution (i.e., how rapidly it decays and truncates).

4.2.2 Insertion-based fission

Fission due to insertions can be described analogously by taking the continuum limit of the nonlocal component of Equation SN6 after removing the local transitions included in Equations SN21 and SN26. Note that, in this case, the length dependence of the per-target rate ι_L appears under the sum.

$$\begin{aligned}(\Delta_\iota \mathrm{P}_L)_{fiss} &= -(L-2)\iota_L \mathrm{P}_L + \sum_{l=L+2}^{\infty} 2\iota_l P_l \\ &\approx -(L-2\Delta L)\iota_L \rho(L) + \int_{L+2\Delta L}^{\infty} 2\iota_\lambda \rho(\lambda) d\lambda \\ &\approx -L\iota_L \rho(L) + \int_{L}^{\infty} 2\iota_\lambda \rho(\lambda) d\lambda\end{aligned} \tag{SN30}$$

As with substitutions, the target size $L-2$ and limit of summation $l = L+2$ explicitly refer to nonlocal transition away from the repeat boundary, both of which we again subsequently approximate as L in the continuum under the asymptotic assumption $L \gg \Delta L = 1$. The resulting contributions resemble Equation SN29, but the length-dependent function ι_L alters the length scaling of both terms (e.g., using the parameterization in Equation SN1, the outflux scales as $L^{1+\tau_\epsilon}$). This results in a weighted integral over the tail of the distribution, which increases the rate of fission into the focal class, despite a finite target size of two per repeat. This is important to our asymptotic analysis, as the net direction of fission, i.e., the relative weight of the two terms in Equation SN30, now depends explicitly on the exponent τ_ϵ.

4.3 Repeat fusion under random sampling of the length distribution

Repeat fusion, the process by which either μ substitutions or, at a lower rate, deletions of length one B strings (henceforth *B-dels* for brevity) result in the merging of two shorter repeats into a longer repeat, substantially complicate our model of repeat length dynamics. For simplicity, we only describe μ substitution-based fusion (as shown in Equation SN8), but note that differences between substitution- and B-del-generated fusion are entirely analogous to those between substitution- and insertion-generated fission. The only notable exception is that we found no evidence that the rate of deletions between repeats (namely, deletions of single-nucleotide or single-unit interruptions) harbor any dependence on the length of the repeat. Additionally, empirical estimates of *de novo* rates from trio data indicate that the per-target rate of B-dels (roughly 2×10^{-10} per generation) is suppressed relative to the per-target rate for μ substitutions (roughly 4×10^{-9} per generation; see **Methods**) by more than an order of magnitude, suggesting B-dels provide an entirely negligible correction to substitution-based fusion rates. Henceforth, all discussion of fusion is focused on substitution-generated events.

Fusion can only occur due to mutations at B sites immediately adjacent to two A sites and thus occur at a rate proportional to the fraction B strings of single-unit length p_F (see Equation SN8 and subsequent discussion below Equation SN14). In the absence of insertions, this probability is given by $p_F = \mu/(\mu + \nu)$

bioRxiv preprint doi: https://doi.org/10.1101/2025.01.09.631797; this version posted January 10, 2025. The copyright holder for this preprint (which was not certified by peer review) is the author/funder, who has granted bioRxiv a license to display the preprint in perpetuity. It is made available under aCC-BY 4.0 International license.

(expansions and contractions of A repeats do not alter this rate). Insertion could in principle alter this rate, but, under the assumption that non-motif insertions are restricted to single-unit lengths, the inclusion of insertions results in a (slightly) greater influx into the $L = 1$ class of B strings. This amounts only to an additional source at the low length boundary; while this explicitly changes the total number of B strings, the normalized probability distribution remains geometric (i.e., Equation SN14) with a slightly modified rate constant. We confirmed via our computational model that this geometric distribution is nearly identical to that under two-way substitution alone, due to the overwhelming mass in the length one class and a negligible influx due to the low rate of insertions from longer repeats. However, we note that the computational model is an abstraction that assumes B-strings are not susceptible to repeat instability. In practice, the value of p_F could be better estimated from the empirical distribution of B strings, but this was unnecessary in the current setting, as any such estimate does not impact our analysis or results.

We proceed by taking the continuum limit of Equation SN8 after removing local contributions (i.e., those proportional to $(1 - p_F)$).

$$\begin{aligned}(\Delta_\mu)_{fuse}\mathrm{P}_L &= -2\mu p_F \mathrm{P}_L + \mu p_F \sum_{k=1}^{L-2} P_k P_{L-1-k} \\ &\approx -2\mu p_F\ \rho(L) + \mu p_F \int_{\Delta L}^{L-2\Delta L} \rho(\lambda)\rho(L-\Delta L-\lambda)d\lambda \\ &\approx -2\mu p_F \rho(L) + \mu p_F \int_0^L \rho(\lambda)\rho(L-\lambda)d\lambda \end{aligned} \tag{SN31}$$

As written, the sum contains an implicit factor of two associated with swapping the subscripts $k \leftrightarrow L-1-k$ (i.e., double counting when summing up to $k = L-2$), which propagates to the integral (i.e., $\lambda \leftrightarrow L-\lambda$ when integrating to L). This contribution to changes in repeat length is explicitly nonlocal due to the quadratic, integral dependence on the distribution, which describes randomly sampling shorter repeats of the appropriate length. In the second line, we have again taken the large L asymptotic limit $L \gg \Delta L = 1$ to suppress subdominant terms. In this form, it becomes clear that the integral is simply a convolution of the distribution $\rho(L)$ with itself over a finite length window; this can be readily interpreted as the distribution of the sum of two random lengths (notably, $< L$) both drawn from the same probability distribution $\rho(L)$ (albeit not necessarily normalized appropriately due to non-conservative transitions). Noting that the p_F is strictly less than one and that empirical estimates show $\mu < \nu$, the rate of outflux due to fusion is strictly less than the outflux of fission due to substitution alone; this contribution becomes negligible in the long length regime due to the finite target size for fusions (i.e., $2\mu p_F \ll \nu L$). However, the integral terms in each cannot be analogously compared due to their non-overlapping limits and distinct functional form. Crucially, through exploration of the parameter space via our computational model, we observed that the relative contribution of fusion to the dynamics in the long length regime is generically subdominant to the rate of other transitions for all parameter combinations. This suggests that fusion may be an infrequent event leading to subdominant corrections to the dominant dynamics.

4.4 Steady-state condition for long repeat dynamics

Collecting the continuum approximations to each term of Equation SN9, the continuous-time dynamics of long repeats in the asymptotic large L regime can be described by the following partial differential equation (PDE).

$$\begin{aligned}\partial_t\rho(L) \approx &\left\{\frac{1}{2}\partial_L^2\left[\left((\epsilon_L+\kappa_L)L+2\iota_L+2(\mu(1-p_F)+\nu)\right)\rho(L)\right] - \partial_L\left[\left((\epsilon_L-\kappa_L)L-2\iota_L+2(\mu(1-p_F)-\nu)\right)\rho(L)\right]\right\}_{local} \\ &+\left\{-(\nu+\iota_L)L\rho(L)+\int_L^\infty 2(\nu+\iota_\lambda)\rho(\lambda)d\lambda\right\}_{fiss} + \left\{-2\mu p_F\rho(L)+\mu p_F\int_0^L \rho(\lambda)\rho(L-\lambda)d\lambda\right\}_{fuse}\end{aligned} \tag{SN32}$$

bioRxiv preprint doi: https://doi.org/10.1101/2025.01.09.631797; this version posted January 10, 2025. The copyright holder for this preprint (which was not certified by peer review) is the author/funder, who has granted bioRxiv a license to display the preprint in perpetuity. It is made available under aCC-BY 4.0 International license.

The above expression is again left in terms of general length-dependent rates of expansion, contraction, and insertion and appropriately labeled to differentiate local, fission-, and fusion-based contributions. For completeness, we have reintroduced local contributions from insertion and substitution (these subdominant effects will be dropped again shortly). Setting the time derivative to zero, we find an integral ordinary differential equation (ODE) for the steady state distribution.

$$\partial_t \rho_{ss}(L) = 0 \tag{SN33}$$

Henceforth, the subscript ss (indicating steady state) will be dropped and assumed throughout. We now approximate this condition to focus on the dominant terms driving the distribution at large lengths. Again, substitutions and insertions are subdominant in the local terms due to a length-independent target size. Additionally, $\nu L \gg 2\mu p_F$ such that the fusion outflux remains subdominant at long lengths. These approximations yield a slightly simpler steady state condition in the asymptotic $L \gg 1$ regime.

$$\partial_t \rho(L) \approx \frac{1}{2}\partial_L^2 \left[(\epsilon_L + \kappa_L) L\rho(L)\right] - \partial_L \left[(\epsilon_L - \kappa_L) L\rho(L)\right] - (\nu + \iota_L)\, L\rho(L) + \int_L^\infty 2\,(\nu + \iota_\lambda)\,\rho(\lambda)d\lambda + \mu p_F \int_0^L \rho(\lambda)\rho(L-\lambda)d\lambda \approx 0 \tag{SN34}$$

Expressing this in terms of our power law parameterization, we find the following.

$$\frac{1}{2}\partial_L^2 \left[\left(C_\epsilon L^{\tau_\epsilon} + C_\kappa L^{\tau_\kappa}\right) L\rho(L)\right] - \partial_L \left[\left(C_\epsilon L^{\tau_\epsilon} - C_\kappa L^{\tau_\kappa}\right) L\rho(L)\right] - (\nu + C_\iota L^{\tau_\epsilon}) L\rho(L) + \int_L^\infty 2\left(\nu + C_\iota \lambda^{\tau_\epsilon}\right)\rho(\lambda)d\lambda + \mu p_F \int_0^L \rho(\lambda)\rho(L-\lambda)d\lambda \approx 0 \tag{SN35}$$

Importantly, no generic closed-form solution to this ODE can be found. First, the complications introduced by fusion are significant, as integration must be performed over the short repeat length regime, limiting our ability to decouple the long length asymptotic dynamics. Second, even when omitting fusion entirely, the remaining terms describe a second-order integral ODE, which can be recast as a third order ODE to remove explicit nonlocal transitions; unfortunately, few third order ODEs are exactly soluble.

The functional form of the fusion term fundamentally limited our ability to further disentangle the dynamics. Motivated by our computational results, we proceeded under the *ansatz* that, for long lengths $L \gg 1$, the fusion term is everywhere negligible relative to fission and local transitions, the latter providing the largest contributions to the asymptotic dynamics due to length scaling. The validity of this assumption, which we confirmed by exploring the allowed parameter space with our computational model, is likely a consequence of both the low rate associated with $\mu p_F \ll \nu L$ and the rapid geometric decay in the short length regime followed by the further (though sub-geometric) monotonic decay at longer lengths. In contrast, Equation SN15 demonstrates the importance of fusion to the short length dynamics, which exactly balance length decreases due to ν substitutions. Exploring a wide range of parameters, we found non-negligible contributions from fusion only in the substitution-dominated short length regime, consistent with our analytic results, and in cases where the distribution is far from steady state. A more principled argument for the relative suppression of fusion at long lengths likely exists, but is unnecessary for the present purposes. For further analysis, we proceed under the following approximation of Equation SN35 for $L \gg 1$, which retains nonlocal transitions only in the form of repeat fission.

$$\frac{1}{2}\partial_L^2 \left[(\epsilon_L + \kappa_L) L\rho(L)\right] - \partial_L \left[(\epsilon_L - \kappa_L) L\rho(L)\right] - (\nu + \iota_L)\, L\rho(L) + \int_L^\infty 2\,(\nu + \iota_\lambda)\,\rho(\lambda)d\lambda \approx 0 \tag{SN36}$$

Expressed in terms of our parameterization, steady state is maintained under the following approximation.

$$\frac{1}{2}\partial_L^2 \left[\left(C_\epsilon L^{\tau_\epsilon} + C_\kappa L^{\tau_\kappa}\right) L\rho(L)\right] - \partial_L \left[\left(C_\epsilon L^{\tau_\epsilon} - C_\kappa L^{\tau_\kappa}\right) L\rho(L)\right] - \left(\nu + C_\iota L^{\tau_\epsilon}\right) L\rho(L) + \int_L^\infty 2\left(\nu + C_\iota \lambda^{\tau_\epsilon}\right)\rho(\lambda)d\lambda \approx 0 \tag{SN37}$$

bioRxiv preprint doi: https://doi.org/10.1101/2025.01.09.631797; this version posted January 10, 2025. The copyright holder for this preprint (which was not certified by peer review) is the author/funder, who has granted bioRxiv a license to display the preprint in perpetuity. It is made available under aCC-BY 4.0 International license.

The above expression describes four distinct effects that collectively lead to steady state, parameters permitting: bidirectional diffusion due to net repeat instability from the combined effects of expansion and contraction, expansion-contraction bias generating a directional flux, net (substitution- and/or insertion-based) outflux due to repeat fission, and net nonlocal (substitution- and/or insertion-based) influx due to fission of any longer repeats. The outflux due to fission is, strictly speaking, a local term in the equation (despite representing non-local transitions, appropriately accounted for by the nonlocal influx) that corresponds to the rate of interruptions of repeats in the focal length class.

4.5 Decomposition of parameter space into dynamical regimes

To better understand the dynamics, we characterized the behavior in qualitatively distinct parameter regimes as primarily controlled by a sum of two, three, or four of the terms in the more complete steady state approximation shown in Equation SN37. These regimes can be identified by studying the length scaling associated with each term, which indicates the primary difference between local and fission-based contributions to changes in length. In contrast to the generality of Equation SN34 (and, assuming subdominant fusion, Equation SN36), the following decomposition is dependent on the details of both our empirically estimated mutation rates and the parameterization in Equation SN1; differing estimates or parameterizations may result in differences in the quantities of importance to the dynamics, but are unlikely to fundamentally alter the subsequent qualitative conclusions about the steady state distribution.

4.5.1 Asymptotic length dependence of local transition rates and $\Delta\tau$

First focusing on local length changes, the quantity $\Delta\tau$ (see Equation SN24) provides an indicator variable for the sign of the bias term at asymptotically large lengths. This can be seen by manipulating the length dependence inside the first derivative term representing the directional (i.e., signed) per repeat rates.

$$(\kappa_L L - \epsilon_L L) = (C_\kappa L^{\tau_\kappa} - C_\epsilon L^{\tau_\epsilon})L = (C_\kappa L^{\Delta\tau} - C_\epsilon)L^{1+\tau_\epsilon} \tag{SN38}$$

The sign of this term determines the asymptotic dominance of either expansion or contraction (i.e., the bias at long lengths) and is dependent only on $\Delta\tau$ (i.e., the constants C_ϵ and C_κ do not scale with length). For clarity, the $\Delta\tau \equiv \tau_\kappa - \tau_\epsilon$ was defined to correspond to the sign of $\kappa_L - \epsilon_L$ such that positive $\Delta\tau$ indicates asymptotic contraction bias.

$$\lim_{L\to\infty} \text{sign}[\kappa_L - \epsilon_L] = \text{sign}[\Delta\tau] \tag{SN39}$$

As described in Equation SN23, $\Delta\tau$ (provided $\Delta\tau \neq 0$) specifies a length L^* at which the sign of $\kappa_L - \epsilon_L$ can reverse if the directional flux changes above $L = 9$ (relative to the initially expansion-biased rates at low lengths, $\epsilon_{L=9} > \kappa_{L=9}$). The (overly simplistic) power law parameterization limits the behavior to, at most, one such sign change in the long length regime: asymptotic contraction-bias ($\Delta\tau \geq 0$) displays one sign reversal, while asymptotic expansion bias ($\Delta\tau \leq 0$) depicts a positive definite (i.e.,, non-vanishing) directional flux throughout the long length regime (due to the empirically estimated expansion bias at $L = 8$). The asymptotic magnitude of the directional flux is determined by the scaling of the faster of the two rates.

$$\lim_{L\to\infty} (\kappa_L - \epsilon_L)\, L \propto \text{sign}[\Delta\tau] m L^{1+\max[\tau_\epsilon,\tau_\kappa]} \tag{SN40}$$

Here, we have included the linear dependence on m common to the constants C_ϵ and C_κ, but focus on the length dependence. This asymptotic dependence defines another useful variable that characterizes the asymptotic magnitude of the local flux $\tau_{\max}$, the maximum between τ_ϵ and τ_κ, which describes the more rapidly growing rate.

$$\tau_{\max} \equiv \max[\tau_\epsilon, \tau_\kappa] \tag{SN41}$$

bioRxiv preprint doi: https://doi.org/10.1101/2025.01.09.631797; this version posted January 10, 2025. The copyright holder for this preprint (which was not certified by peer review) is the author/funder, who has granted bioRxiv a license to display the preprint in perpetuity. It is made available under aCC-BY 4.0 International license.

As can be seen in Equation SN28, $\tau_{\max}$ also dictates the asymptotic dependence of the (strictly positive) diffusion coefficient in the second derivative term.

$$\lim_{L\to\infty}(\epsilon_L + \kappa_L)L \propto mL^{1+\max[\tau_\epsilon,\tau_\kappa]} \tag{SN42}$$

When $\|\Delta\tau\| \gg 1$, either due to large expansion or contraction rates, the asymptotic rates of diffusion and bias increase in magnitude in the same way (provided $L > L^*$ for $\Delta\tau > 0$).

While the quantities $\Delta\tau$ and $\tau_{\max}$ together characterize the asymptotic behavior of both local contributions to the dynamics, their values are related. The sign of $\Delta\tau$ dictates the value of $\tau_{\max}$: for $\Delta\tau < 0$, $\tau_{\max} = \tau_\epsilon$, for $\Delta\tau > 0$, $\tau_{\max} = \tau_\kappa$, and for $\Delta\tau = 0$, $\tau_{\max} = \tau_\epsilon = \tau_\kappa$. Additionally, because the values of τ_ϵ and τ_κ are positive semidefinite, allowed values of $\Delta\tau$ and $\tau_{\max}$ are correlated in the biologically-plausible positive quadrant (i.e., in the space $(\Delta\tau, \tau_{\max}) > (0,0)$). For example, the line defining $\Delta\tau = 1$ requires that $\tau_\kappa \geq 1$, which imposes the same constraint on $\tau_{\max} \geq 1$ (for positive $\Delta\tau$, $\tau_{\max} = \tau_\kappa$). The asymptotic magnitudes of the length-dependent rates of diffusion and directional flux are inherently bounded by the intersections with the axis at $\tau_\epsilon = 0$ or $\tau_\kappa = 0$ for $\Delta\tau > 0$ or $\Delta\tau < 0$, respectively, in the positive quadrant of interest.

$$\tau_{\max} \geq |\Delta\tau| \tag{SN43}$$

In this way, dynamical regimes associated with large $\tau_{\max}$ can be identified and bounded by $\Delta\tau$ values, provided the behavior at the intersection (either $\tau_\epsilon = 0$ or $\tau_\kappa = 0$) corresponds to similar dynamics at larger values of $\tau_{\max}$ (as can be seen in our computational model). Accordingly, subsequent decomposition of the parameter space is phrased in terms of diagonals associated only with constant values of $\Delta\tau$ (i.e., $\tau_\kappa = \tau_\epsilon + const.$), which adequately characterizes asymptotic properties of all local changes in repeat length. As the linear dependence on m is common to all local terms, it is unimportant for distinguishing between these terms; however, the dependence of fission on the substitution rate ν indicates that this parameter may inform comparisons between the rates of local and nonlocal changes in length.

4.5.2 Relative strengths of substitution- and insertion-driven fission and L_{fis}

In contrast to the local dynamics, repeat fission is asymptotically dominated by insertion, which outcompetes the substitution rate at asymptotically long lengths. A characteristic length L_{fis} emerges, above which the fission rate is dominated by insertion-based interruptions. This length can be found by comparing the per-target rates of substitution ν and insertion $C_\iota L^{\tau_\epsilon}$.

$$L_{\text{fis}} \equiv \left(\frac{\nu}{C_\iota}\right)^{1/\tau_\epsilon} = 9\left(\frac{\nu}{m \times \iota_8}\right)^{1/\tau_\epsilon} \tag{SN44}$$

Here, we used the definition of C_ι to show the explicit dependence on m. Our empirical estimate of the ratio ν/ι_8 is of order 50 for mononucleotide A-repeats (see **Figure 2a**); thus, for values of $m \lesssim 50$ (spanning the computationally explored parameter space), the ratio being exponentiated is greater than one. Consequently, as τ_ϵ increases, L_{fis} decreases, eventually exiting the long length regime (i.e., $L_{\text{fis}} < 9$ for $\tau_\epsilon \gg 1$) such that all long length fission events are insertion-dominated. Substitutions dominate more classes in the long length regime when both τ_ϵ and m are small (i.e., for $\tau_\epsilon < 1$ and $m \ll 50$), while substitutions are subdominant at more long lengths when τ_ϵ and m are large. Our computational inferences allow for a wide range of L_{fis} values, from substitutions remaining negligible at all long lengths to substitutions generating effectively all fissions of long repeats. Although intermediate values $m = 4 - 16$ are most consistent with empirical data, our inference showed a wide range of statistically consistent τ_ϵ values that include both $L_{\text{fis}} \sim 10$ (e.g., when $m \sim 16$ and $\tau_\epsilon \gtrsim 2.3$, insertions dominate for $L > L_{\text{fis}} \lesssim 15$) and L_{fis} values beyond the populated length classes (e.g., when $m = 4$ and $\tau_\epsilon < 1$, $L_{\text{fis}} > 100$ and substitutions dominate). This variation in qualitative behavior suggests that our inference has little power to decompose fission into distinct mutational types using available empirical data.

The quantities τ_ϵ and m together characterize the asymptotic length dependence of repeat fission by determining the value of L_{fis}, the length at which a switch occurs from substitution- to insertion-dominated

bioRxiv preprint doi: https://doi.org/10.1101/2025.01.09.631797; this version posted January 10, 2025. The copyright holder for this preprint (which was not certified by peer review) is the author/funder, who has granted bioRxiv a license to display the preprint in perpetuity. It is made available under aCC-BY 4.0 International license.

fission (at lengths $L < L_{\text{fis}}$ and $L > L_{\text{fis}}$, respectively). In the parameter regime where fission is entirely insertion-dominated at long length (i.e., $L_{\text{fis}} \sim 10$ when m, τ_ϵ, or both parameters are sufficiently large), all relevant terms in Equation SN37 are linear in m such that the dynamics are independent of this parameter; in this case, the relative importance of local transitions and fission can be characterized by the values of τ_ϵ and $\Delta\tau$ (or, equivalently, τ_ϵ and τ_κ). In this extreme, in addition to the decoupling between short and long repeat dynamics, long repeats are predominantly subject to insertion- and deletion-based mutations (i.e., repeat instability-generated expansions, contractions, and insertions) and evolve *mechanistically* independently (i.e., change length predominantly due to distinct mutational processes) from short repeats; importantly, this claim implicitly requires that long repeat dynamics are independent of μ (i.e., fusion remains negligible). In the opposite extreme in which insertions remain irrelevant at all populated lengths, the dynamics are independent of τ_ϵ and can be represented in the space of $(\Delta\tau, m)$. Our inference from empirical observations suggests that we cannot simultaneously determine the values of $\{m, \tau_\epsilon, \Delta\tau\}$ (equivalently, $\{m, \tau_\epsilon, \tau_\kappa\}$) and must rely on all three parameters to represent the space of empirically consistent dynamical models.

4.5.3 Distinguishable dynamical regimes

First, the sign of $\Delta\tau$ decomposes the space into two primary regions and the boundary between them, only one of which evolves towards a recognizable steady state equilibrium on reasonable timescales. The repeat length distribution only stabilizes for the subset of the parameter space with $\Delta\tau \leq 0$, which we refer to as the (asymptotically) *contraction-biased* regime. The intuitive explanation for this is that positive directional flux ($\Delta\tau < 0$) leads to indefinite repeat expansion in excess of the net shortening effects of repeat fission (the boundary at $\Delta\tau = 0$ is discussed below). In contrast, asymptotic contraction bias truncates the distribution at finite lengths, leading to a stable distribution, as was observed empirically. The collection of stable parameter combinations can be decomposed into parameter ranges exhibiting distinct dynamics that depend on the extent to which contraction dominates over expansion (i.e., the value of $\Delta\tau$). Within each subregime of $\Delta\tau > 0$, the full set of changes in repeat length in Equation SN32 that lead to a stable distribution can be reduced to a subset of effects that approximate the dominant contributions that shape the distribution; parameter combinations with $\Delta\tau \gg 1$ are controlled largely by a local balance between diffusion and a largely contraction-biased directional flux, both of which dominate over fission; intermediate values $1 \gtrsim \Delta\tau > 0.6$ show a balance between local transitions and length-decreasing outflux due to fission (from substitutions, insertions, or both); weak contraction bias (very roughly $\Delta\tau \lesssim 0.6$) requires a full accounting of local effects and both the influx and outflux due to fission (i.e., the full set of effects included in Equation SN37). At all points in the stable regime, the net increase in repeat length due to expansion (with minor local contributions from μ substitutions) is counteracted by the combined effects of contraction, which dominates over expansion above some length L^*, and insertions and ν substitutions in the repeat body that lead to fission. Based on our computational model, no stable distribution was maintained due to appreciable fusion, consistent with our approximation in Equation SN37. The boundary of this regime occurs near $\Delta\tau \approx 0$; however, very low positive values of $\Delta\tau$ (e.g., roughly $0.3 > \Delta\tau > 0$ when $m = 8$) remain expansion dominated across a large range of populated lengths because the intersection of the rates at $L^* \to \infty$ as $\Delta\tau \to 0$ (see Equation SN23). This results in unrealistic models inconsistent with the empirical distribution, as the asymptotic contraction bias is only apparent for repeats much longer than those observed in appreciable numbers in the human genome.

The remaining set of parameter combinations fall into two categories, both of which are implausible with respect to the collection of empirical observations presented in this manuscript: those with very slowly evolving distributions that do not equilibrate on evolutionarily-relevant timescales and those with unstable dynamics that lead to a rapid aggregation of repeats and consequent explosive growth in the length of the genome. Exceedingly slowly evolving dynamics occur for small values of m as both exponents approach zero (i.e., at values $\tau_\epsilon, \tau_\kappa < 1$ such that τ_ϵ and $\|\Delta\tau\|$ both remain small). These parameter combinations depict repeat instability rates inconsistent with empirical rate estimates (see **Figure 2a**) and, given indefinite time to evolve (in excess of the divergence timescale for primates), are unlikely to result in a stable distribution consistent with those observed across the primate phylogeny. We refer to this region of parameter space as the *slowly evolving* regime, as the repeat instability rates depicted remain scantly above the estimated

bioRxiv preprint doi: https://doi.org/10.1101/2025.01.09.631797; this version posted January 10, 2025. The copyright holder for this preprint (which was not certified by peer review) is the author/funder, who has granted bioRxiv a license to display the preprint in perpetuity. It is made available under aCC-BY 4.0 International license.

substitution rates, even for the longest well-populated lengths in the human genome. Parameter combinations that are dynamically unstable (denoted as the *unstable* regime) universally correspond to expansion rate-dominated dynamics ($\Delta\tau < 0$, excluding slowly evolving parameters with low τ_ϵ). This regime comprises the majority of the parameter space explored in our computational model, which can be dynamically disallowed under the sole observation of steady state, as they represent non-equilibrium dynamical regimes subject to strong nonlinear effects that result in increasingly rapid changes in the repeat length distribution. Under our parameterization, parameters with $\Delta\tau = 0$ (away from the slowly evolving regime) are similarly unstable due to an expansion-bias inherited from the empirically observed bias at $L = 8$ (i.e., $\kappa_9 - \epsilon_9 = m(\kappa_8 - \epsilon_8) > 0$) and subsequent parallel growth of expansion and contraction.

Last, we note that the decomposition of the dynamics is dramatically simplified for large multipliers $m \gtrsim 8$ due to the location of L_{fis}, which sits below the long length regime for sufficiently large m (see Equation SN44). We focus our discussion of the analytics on this case, where the dynamics can be characterized largely by $\Delta\tau$ alone and substitutions are subdominant to insertions for long repeats. However, the same dynamics apply to smaller m, values with the additional complication that the balance between substitutions and insertions breaks the dynamical similarity along lines of constant $\Delta\tau$.

4.6 Unstable dynamics in the asymptotically expansion-biased regime $\Delta\tau \leq 0$

We briefly discuss the dynamics of the unstable regime, as it informs our considerations for more realistic parameter combinations. There are two distinguishable cases where steady state cannot be assumed, contradicting the observation of long term maintenance of the distribution across the primate phylogeny. First, the expansion rate, which is initially dominant at $L = 9$ (i.e., $\epsilon_9 > \kappa_9$), may have a length dependence that rapidly outcompetes that of contraction, resulting in increasingly larger expansion-bias with increasing length. This corresponds to $\Delta\tau < 0$ with large magnitudes $\|\Delta\tau\| \gg 1$. In the second case, which occurs for relatively small values of $\|\Delta\tau\|$ approaching $\Delta\tau = 0$ (i.e., when $\tau_\epsilon = \tau_\kappa$) the length dependence of expansion increases with length comparably to, or slightly in excess of, the length-dependence of the contraction rate. In this case, the directional flux is minimized such that the relative importance of repeat fission becomes inflated. In the former case with highly dominant expansion, the bias generates a large directional flux that rapidly increases repeat lengths. In this regime, the flux is well approximated by the rate $C_\epsilon L^{1+\tau_\epsilon}$ (i.e., $C_\kappa L^{1+\tau_\kappa}$ detracts negligibly at all lengths $L > 10$) and the rate of length increase rapidly accelerates with increasing length. This nonlinearity results in an indefinitely extending tail, some of which feeds back into lower length classes due to fission, as large τ_ϵ also generates large rates of insertion-based repeat fission $C_\iota L^{1+\tau_\epsilon}$; however, the coefficient C_ι is roughly two orders of magnitude smaller than C_ϵ such that fission alone is unable to counteract expansion alone at any length. Repeat fission additionally increases the mass of the distribution, as it is a nonconservative transition: one repeat is replaced with two shorter length repeats. These shorter repeats are then subject to the large directional push due to expansion, which further increases the weight in the distribution tail. This feedback loop generates a rapid, indefinitely growing genome, which reshapes the distribution; rapid fission of any given repeat in the long tail equiprobably adds mass to the distribution in all shorter length classes, an integrated effect that is increased with increasing mass above a given length. This eventually leads to an extreme influx into the shortest length classes, inherently coupling the dynamics in the short and long length regimes (i.e., violating our assumption of separability and distorting the substitution-based geometric distribution). In addition to the unstable dynamics, the indefinite extension of the distribution tail quickly results in highly relevant expansion probabilities approaching one, which simultaneously makes computational modeling impractical across this regime and will invariably result in characteristic changes to the shape of the distribution as the power law parameterization of the rates must give way to saturation at this probabilistic bound.

In the second case, for $\Delta\tau$ near zero (again assuming sufficiently large τ_ϵ to avoid the slowly evolving regime), the directional flux is again expansion dominated and non-vanishing. Here, the relative importance of fission is inflated, which limits the rate at which the tail extends. However, the difference between the expansion and contraction rates, even at $L = 8$, is substantially in excess of the insertion rate. This leads to

bioRxiv preprint doi: https://doi.org/10.1101/2025.01.09.631797; this version posted January 10, 2025. The copyright holder for this preprint (which was not certified by peer review) is the author/funder, who has granted bioRxiv a license to display the preprint in perpetuity. It is made available under aCC-BY 4.0 International license.

the inability of repeat fission to independently counteract the directional flux at all lengths.

$$(C_\epsilon - C_\kappa)L^{1+\tau_\epsilon} \gg C_\iota L^{1+\tau_\epsilon} \tag{SN45}$$

In this sense, contraction must be sufficiently large to mitigate expansion in order for the distribution to truncate at finite length. This only occurs when $\Delta\tau > 0$ such that the contraction rate approaches, and eventually exceeds, the expansion rate (though this may occur at an extremely large length for the smallest values of $\Delta\tau > 0$). This defines a bound for the contraction-biased regime that leads to steady state. A small non-negligible contribution from fission is relevant above this bound, but is insufficient to control the directional push from expansion for very low $\|\Delta\tau\|$. We note that this effort may be aided by substitution-based fission, but the associated length scaling (i.e., the fission rate νL) only becomes relevant for small multipliers m when $\tau_\epsilon \lesssim 1$ (i.e., when $\nu/C_\iota \sim \mathcal{O}(1)$ and $\nu/C_\iota L^{\tau_\epsilon} \sim \mathcal{O}(1)$ across relevant lengths); parameters in this range correspond to sufficiently small rates that evolution proceeds exceedingly slowly, as discussed above.

4.7 Stable dynamics in the asymptotically contraction-biased regime $\Delta\tau > 0$

In contrast to the unstable regime, parameter combinations with asymptotic contraction bias $\Delta\tau > 0$ result in a distribution that approaches a stable steady state. Perhaps more intuitively, this regime can be equivalently characterized by L^*, the length at which expansion and contraction rates cancel such that the directional flux vanishes. For all values $\Delta\tau > 0$, evaluation of Equation SN23 shows that $L^* \geq 9$ (and $L^* \geq 10$ for realistic values of $\Delta\tau$) such that this intersection sits within, or near the boundary of, the long length regime. As the combination of our empirical estimates at $L = 8$ and our parameterization (namely, $m > 0$) dictate that the expansion rate exceeds the contraction rate at length $L = 9$, an intersection between the rates must occur prior to the asymptotic dominance of contraction. As a result, repeat lengths below L^* are expansion-biased and those above L^* are contraction-biased, with a vanishing directional flux at L^*. For large values of $\Delta\tau$, the value of L^* approaches a constant close to 10. For very small values, the location can sit at very large lengths, approaching $L^* \to \infty$ as $\Delta\tau \to 0$. At many values of $\Delta\tau > 0$, this occurs at lengths where the distribution is well-populated, assuming a total target size comparable to the length of the human genome.

The relative rates of expansion and contraction may remain on the same order of magnitude for smaller $\Delta\tau$, as the approach to this intersection is slow from either side; as a result, the first derivative term remains relatively small over a range of lengths that span part or all of the distribution tail, defining an extended length range associated with low directional flux. In this case, the diffusion term, which arises as a subdominant correction to the discrete local behavior (see Section 4.1.2) plays an important role in the extended neighborhood of L^*. This transition between expansion- and contraction-biased length ranges controls and complicates the dynamics. For the smallest values of $\Delta\tau \ll 1$, particularly for large values of $\tau_{\max}$, the intersection at L^* occurs at extreme lengths above those populated in the empirical distribution. This results in the tail extending dramatically due to a wide range of expansion-biased lengths until contraction bias becomes appreciable. Additionally, this generates a dramatically larger genome (with an excess of very long repeats) inconsistent with the observed range of mammalian genome sizes. This is somewhat similar to the unstable dynamics described above, but is eventually counteracted by sufficiently large contraction rates that truncate the distribution and stabilize the (presumably unrealistic) shape of the distribution.

As discussed above, the dynamics of long repeats across the space of parameters with $\Delta\tau > 0$ can be approximated by Equation SN37 under the assumption that repeat fusion is sufficiently infrequent. However, the dynamics reduce further in parts of this regime where fission (influx and, to a lesser extent, outflux) can be treated as negligible. Such approximations eliminate the need to explicitly treat the nonlocal effects of fission influx, which reduces the second-order integro-differential equation to a second-order ODE.

4.7.1 Strong asymptotic contraction bias

We first attempted to describe the dynamics in the regime with sufficiently large $\Delta\tau$ such that the majority of long repeats have contraction-biased rates (i.e., $L^* \sim 10$ occurs immediately above the short repeat regime).

bioRxiv preprint doi: https://doi.org/10.1101/2025.01.09.631797; this version posted January 10, 2025. The copyright holder for this preprint (which was not certified by peer review) is the author/funder, who has granted bioRxiv a license to display the preprint in perpetuity. It is made available under aCC-BY 4.0 International license.

In this case, the diffusion term remains relevant because L^* lies in the long length regime but the directional flux becomes increasingly relevant with increasing length, an effect magnified by necessarily larger $\Delta\tau$ (and consequently larger $\tau_{\max}$). For most contraction-biased parameter combinations, the dynamics of Equation SN37 are well approximated by the following.

$$\frac{1}{2}\partial_L^2\left[(\epsilon_L+\kappa_L)L\rho(L)\right]-\partial_L\left[(\epsilon_L-\kappa_L)L\rho(L)\right]-(\nu+\iota_L)\,L\rho(L)\approx 0 \tag{SN46}$$

Under the power law parameterization, this becomes the following.

$$\frac{1}{2}\partial_L^2\left[\left(C_\epsilon L^{\tau_\epsilon}+C_\kappa L^{\tau_\kappa}\right)L\rho(L)\right]-\partial_L\left[\left(C_\epsilon L^{\tau_\epsilon}-C_\kappa L^{\tau_\kappa}\right)L\rho(L)\right]-\left(\nu+C_\iota L^{\tau_\epsilon}\right)L\rho(L)\approx 0 \tag{SN47}$$

Here, influx due to fission is treated as subdominant, as it is outcompeted by all remaining rates that scale asymptotically with length with an exponent greater than one (i.e., as $L^{1+\tau_\kappa}$ or $L^{1+\tau_\epsilon}$).

4.7.2 Strictly local approximation for strong asymptotic contraction bias $\Delta\tau \gg 1$

In the regime of largest $\Delta\tau$, corresponding to the largest asymptotic scaling $\kappa_L L \pm \epsilon_L L \approx C_\kappa L^{1+\tau_\kappa}$ for the rates of directional flux and diffusion, the outflux due to fission becomes negligible, as well (i.e., it is outcompeted because $\tau_\kappa \gg \tau_\epsilon$). This defines a dynamical sub-regime within the space of contraction-biased parameter values, with dynamics well approximated by the following balance.

$$\frac{1}{2}\partial_L^2\left[\left(C_\epsilon L^{\tau_\epsilon}+C_\kappa L^{\tau_\kappa}\right)L\rho(L)\right]-\partial_L\left[\left(C_\epsilon L^{\tau_\epsilon}-C_\kappa L^{\tau_\kappa}\right)L\rho(L)\right]\approx 0 \tag{SN48}$$

In this regime, sufficiently rapid decay in $\rho(L)$ results in a net negative directional flux (the correct sign associated with net contraction bias) that counteracts the strictly positive diffusion term. For example, when $m = 8$, this expression provides a good approximation to the dynamics primarily for $\Delta\tau > 2$ (equivalently, $\tau_{\max} = \tau_\kappa > 2$) and breaks down as fission becomes increasingly relevant for weaker contraction bias. In this regime, L^* occurs at or adjacent to $L = 10$ such that nearly all long lengths are contraction-biased. For example, the parameter combination $\{m, \tau_\epsilon, \tau_\kappa\} = \{8, 0, 2\}$ results in $L^* \approx 11$ and a contraction rate of roughly double the expansion rate at $L = 16$, while for $\{m, \tau_\epsilon, \tau_\kappa\} = \{8, 0, 4\}$, $L^* \approx 10$ and the contraction rate is roughly tenfold the expansion rate at $L = 16$. In the more extreme case of $\Delta\tau = 4$, we can better understand the truncation of the distribution by further approximating Equation SN48 under the very rough assumption that the asymptotic dependence is immediately relevant for lengths $L > L^*$.

$$\frac{1}{2}\partial_L^2\left[L^{1+\tau_\kappa}\rho(L)\right]\approx -\partial_L\left[L^{1+\tau_\kappa}\rho(L)\right] \tag{SN49}$$

Noting that the contraction rate constant C_κ cancels such that this equation is only dependent on the exponent τ_κ, an approximation for the asymptotic shape of the steady state distribution can be obtained in closed form (valid only for lengths $L \gg L^*$).

$$\lim_{\Delta\tau\gg 1}\rho(L)\approx c_1 L^{-(1+\tau_\kappa)}+c_2 L^{-(1+\tau_\kappa)}e^{-2L} \tag{SN50}$$

To evaluate the accuracy of this rough approximation, the arbitrary constants c_1 and c_2 can be found using values from our computational model at two lengths L_1 and L_2 (where $L_1, L_2 \gg L^*$) to constrain the distribution at $\rho(L_1)$ and $\rho(L_2)$. Comparing this expression to numerical solutions, we found reasonable agreement for the most extreme values of $\Delta\tau > 0$ (e.g., $\Delta\tau = 2-4$ for $m = 8$) at the longest lengths $L > L^*$ in the distribution and an expected departure as $L \to L^*$.

4.8 Intermediate asymptotic contraction bias

Intermediate values of $\Delta\tau > 0$ (e.g., roughly $\Delta\tau \sim 0.8-1.4$ for $m = 8$) require a description of the flux out due to fission, as shown in Equation SN47. At the larger end of this range (e.g., $\Delta\tau \sim 1$), the numerical solution to

bioRxiv preprint doi: https://doi.org/10.1101/2025.01.09.631797; this version posted January 10, 2025. The copyright holder for this preprint (which was not certified by peer review) is the author/funder, who has granted bioRxiv a license to display the preprint in perpetuity. It is made available under aCC-BY 4.0 International license.

Equation SN48, which omits all effects of fission, approximates the asymptotic shape of the distribution for lengths $L > L^*$. This indicates that the impact of outflux due to fission is primarily localized to intermediate lengths $L^* > L > 10$ and therefore most relevant when the rate of expansion exceeds contraction. At the same time, this suggests that the truncation of the distribution is driven by the contraction-biased directional flux, rather than fission alone or requiring the combined effects of fission and contraction.

4.9 Weak asymptotic contraction bias

For smaller values of $\Delta\tau$ that approach $\tau_\epsilon = \tau_\kappa$ (e.g., roughly $\Delta\tau \sim 0.3-0.6$ for $m = 8$), the dynamics revert to Equation SN37. The resulting distributions stabilize, in part, due to a nonlocal influx from fission of longer repeats. For these parameter combinations, L^* sits in the middle of the long length tail; for lengths $L > L^*$, the distribution is well-approximated by solutions to Equation SN47. This is consistent with a nonlocal net flow from lengths $L > L^*$ to lengths $L^* > L > 10$ and indicates that the dynamically relevant effects of fission influx are localized to the latter. The net effects of fission alone (i.e., fission influx minus outflux) result in a net loss of long repeats, with little gain from any longer length repeats that exist, and a compensatory net gain of intermediate length repeats within the distribution tail, in excess of the number lost to the short length regime. With decreasing values of $\Delta\tau \to 0$, L^* approaches very large values, extending the range of lengths receiving this influx. All effects represented in SN37 are thus required to adequately approximate the dynamics that lead to a steady state distribution when $\Delta\tau \ll 1$ (and a potentially broader range of fractional values $\Delta\tau < 1$, depending on m).

The nonlocal integral dependence in Equation SN37 that describes the influx due to fission complicates the steady state condition in this regime. To find solutions, we re-expressed the second-order integro-differential equation as a third order differential equation by applying an overall length derivative to each term.

$$\frac{1}{2}\partial_L^3\left[\left(C_\epsilon L^{\tau_\epsilon} + C_\kappa L^{\tau_\kappa}\right) L\rho(L)\right] - \partial_L^2\left[\left(C_\epsilon L^{\tau_\epsilon} - C_\kappa L^{\tau_\kappa}\right) L\rho(L)\right] - \partial_L\left(\nu + C_\iota L^{\tau_\epsilon}\right) L\rho(L) + \partial_L\left[\int_L^\infty 2\left(\nu + C_\iota \lambda^{\tau_\epsilon}\right)\rho(\lambda)d\lambda\right] \approx 0 \quad \text{(SN51)}$$

Taking this derivative allows us to apply the fundamental rule of calculus to replace the derivative the integral with the integrand evaluated at the bounds of integration. Under the assumption that the distribution decays sufficiently rapidly such that $L^{\tau_\epsilon}\rho(L) \to 0$ as $L \to \infty$, the integral term becomes the following.

$$\partial_L\left[\int_L^\infty 2\left(\nu + C_\iota \lambda^{\tau_\epsilon}\right)\rho(\lambda)d\lambda\right] = -2\left(\nu + C_\iota L^{\tau_\epsilon}\right)\rho(L) \quad \text{(SN52)}$$

This allows us to re-expresses the second-order integro-differential equation as the following third order ODE.

$$\frac{1}{2}\partial_L^3\left[\left(C_\epsilon L^{\tau_\epsilon} + C_\kappa L^{\tau_\kappa}\right) L\rho(L)\right] - \partial_L^2\left[C_\epsilon L^{\tau_\epsilon} - \left(C_\kappa L^{\tau_\kappa}\right) L\rho(L)\right] - \partial_L\left(\nu + C_\iota L^{\tau_\epsilon}\right) L\rho(L) - 2\left(\nu + C_\iota L^{\tau_\epsilon}\right)\rho(L) \approx 0 \quad \text{(SN53)}$$

After applying a length derivative to the steady state condition $\partial_L(\partial_t\rho(L)) = 0$, this now corresponds to a constraint on the flux $\partial_L\rho(L)$. This can be seen by swapping the order of the length and time derivatives (i.e., $\partial_t(\partial_L\rho(L)) = 0$), dictating that the fluxes through each length must sum to a time-independent constant $\phi_L i$. In the special case where $\phi_L = 0$, this corresponds to a steady state condition that maintains the shape of the distribution in equilibrium. We confirmed via our computational model that, once steady state was reached, the net flux through each individual bin independently vanished (see **Figures SN2-SN4**), indicating that the equilibrated state is maintained in a detailed balance. Insofar as our approximations remain valid, Equation SN53 provides a local expression for the steady-state flux through the repeat length distribution in the large length regime; this includes the nonlocal contributions of repeat fission to the flux, represented by boundary effects at length L (more accurately, at length $L + 2 \approx L$). While this equation cannot be solved analytically, numerical solutions can be readily obtained.

bioRxiv preprint doi: https://doi.org/10.1101/2025.01.09.631797; this version posted January 10, 2025. The copyright holder for this preprint (which was not certified by peer review) is the author/funder, who has granted bioRxiv a license to display the preprint in perpetuity. It is made available under aCC-BY 4.0 International license.

Assuming the effects of fusion remain subdominant in the long length regime, Equation SN53 captures the full set of dynamics associated with changes in repeat length. Solutions to this equation, obtained after applying the additional constraint that the fluxes vanish, are applicable across the full range of parameters that evolve towards steady state distributions $\Delta\tau > 0$. In contrast, Equations SN47 and SN48 are approximations to these dynamics appropriate in a subset of parameter space (very roughly, when $\Delta\tau > 1$ and when $\Delta\tau \gg 1$, respectively). However, in addition to aiding in our intuitive understanding of the dynamics, the absence of the third derivative in the latter equations makes numerical solutions more reliable, as they are less susceptible to instabilities in numerical techniques; this facilitates a slightly more reliable comparison between the numerical solutions and results of our computational model.

4.10 Obtaining numerical solutions to the steady state dynamics for $\Delta\tau < 0$

To compare our analytic understanding of the dynamics to the results of our computational model for generic parameters, we resorted to solving Equations SN47, SN48, and SN53 numerically. All numerical solutions were obtained using the NDSolve function in Mathematica 14.0 [1]. Solutions to second-order differential equations require the specification of two additional constraints that together fix the normalization constant and the linear coefficient specifying the relative weight of the two real solutions to the equation, if both exist. The third order equation for constant flux requires a third constraint that ensures that the flux vanishes. For the second-order equations, we chose to constrain the values of $\rho(L)$ at two lengths, L_1 and L_2, using the results of our computational model (i.e., $\rho(L_1) = \rho_{sim}(L_1)$ and $\rho(L_2) = \rho_{sim}(L_2)$, where $\rho_{sim}(L)$ is the value of the computationally propagated (i.e., 'simulated') distribution at length L once it has reached steady state). The choice of L_1 and L_2 is somewhat arbitrary, provided they are both in the long length regime where the continuum approximation is valid and that any additional constraints within the regime of validity for each approximation are respected (e.g., $\Delta\tau > 0$ and sufficiently far from $\Delta\tau = 0$, $\tau_\epsilon, \tau_\kappa \geq 0$, etc.). For the majority of comparisons, we chose two lengths that are well defined for any parameter combination: $L_1 = L^*$ (rounded to the nearest integer value) and $L_2 = L_{\max}$, where $L_{\max}$ is the length bin for which the occupancy of the non-normalized distribution first drops below a single count (i.e., $L_{\max} = \min\left[L \text{ for } \rho(L) < 1\right]$) and represents the truncation point of the distribution. $L_{\max}$ is uniquely defined because all computationally modeled distributions decay monotonically and stochastic effects were omitted to obtain the expected distribution under a mean field approximation. All comparisons were made using the non-normalized distributions to identify the truncation point of the distribution and obtain $L_{\max}$. To provide the third constraint needed for Equation SN53, we chose to use $L_3 = L_2 - 1$ for convenience; we note that inappropriate choice of L_3 outside of the regime of validity of the approximation can result in numerical instability in solutions to the third order ODE (e.g., when constraining the solution using a length class $\rho_{sim}(L_3)$ that has not yet equilibrated). We chose not to use the intuitive lower bound of the long length regime at $L = 10$ to better identify any potential effects associated with the breakdown of the continuum approximation. For any comparisons with $L^* < 10$ (which occur only for unstable parameter combinations with $\Delta\tau < 0$) we chose a lower bound at $L_1 = 10$ to avoid values in the short length regime; however, unstable dynamics were compared to numerical solutions primarily to confirm significant departure from any steady state characterized by Equation SN53 and to identify any common features that emerged. Finally, for all comparisons in the long length regime, the value of ν was replaced with $\nu_{fission}$, the appropriate rate estimated from the three-unit context $AAA \to ABA$ in which substitutions $A \to B$ result in repeat fission. Mutation rates μ and ν that appear in Equation SN10 were replaced with distinctly estimated rates relevant for the three-unit contexts associated with local transitions due to substitutions defined by μ_{local} (i.e., the summed rates of $ABB \to AAB$ and $BBA \to BAA$ substitutions) and ν_{local} (the summed rates of $AAB \to ABB$ and $BAA \to BBA$ substitutions), respectively.

bioRxiv preprint doi: https://doi.org/10.1101/2025.01.09.631797; this version posted January 10, 2025. The copyright holder for this preprint (which was not certified by peer review) is the author/funder, who has granted bioRxiv a license to display the preprint in perpetuity. It is made available under aCC-BY 4.0 International license.

5 Comparison between numerical solutions and computationally modeled distributions

As described above, numerical solutions to Equations SN47, SN48, and SN53 were compared across the space of parameter combinations that led to steady state distributions. Because our computational model obtained results over a large, but finite number of iterations, parameters corresponding to insufficiently high mutation rates failed to equilibrate in the allotted time (i.e, in a number of iterations corresponding to at least 10^9 generations of evolution, after accounting for a factor that progressively rescales time to increase computational speed). These slowly evolving computational results were localized to the lowest values of $\{\tau_\epsilon, \tau_\kappa\}$ for each multiplier m (i.e., points closest to the origin of the $\{\tau_\epsilon, \tau_\kappa\}$ plane) and spanned a larger range of parameter values for smaller m. For $m = 8$, this roughly corresponds to parameter values $\tau_\epsilon, \tau_\kappa \lesssim 1$; within this region, clines of roughly equivalent metric values (calculated by comparing computationally modeled distributions at the final time point to the empirical distribution) begin to deviate from lines of constant $\Delta\tau$. This corresponds to the point at which substitution becomes non-negligible and substitution-based fission occurs at a rate comparable to or greater than insertion-based fission. Given indefinite time to evolve, such parameter combinations would no doubt equilibrate, provided the combined action of contraction, substitution, and insertion is sufficient to truncate the distribution at finite length. As no equilibrium was reached, these points were excluded from our comparison to numerically produced steady-state distributions.

For all points outside of this slowly evolving region, equilibrated steady state distributions are quantitatively similar along lines of constant $\Delta\tau$. The following plots show comparisons at points along an anti-diagonal line perpendicular to $\Delta\tau = 0$ defined by $\tau_\epsilon + \tau_\kappa = 3$. Parameter combinations on this line are representative of the full set of computationally modeled $\Delta\tau$ outside of the slowly evolving region and could be obtained for most multipliers. Along the line of $\tau_\epsilon + \tau_\kappa = 3$, we selected examples that span the qualitatively distinct behaviors across the space of $\Delta\tau > 0$ for values $\Delta\tau = \{0, 0.2, 0.4, 0.6, 0.8, 1, 1.4, 2, 3\}$. The first two points were included for completeness, as they show examples of computationally modeled distributions that are not expected to be well described by numerical solutions. For $\Delta\tau = 0.2$ the location of $L^* \approx 92$, which is close to the maximum length included in our computational model at $L_{boundary} = 200$; a reflective boundary condition was imposed at $L = L_{boundary}$ to simultaneously prevent excessively mutation rates that preclude further computational iteration and to identify parameter combinations that result in excessively large genome size when truncation occurs at unrealistically large lengths $L_{max} > 200$. The point at $\Delta\tau = 0$ was included as an example of unstable dynamics; the computationally modeled distribution equilibrates to the boundary condition at $L_{boundary}$ (note the balanced fluxes in the largest lengths for computational results with $\Delta\tau = 0$ in Figures SN2I and SN3I), resulting in an artifactual shape (i.e., the non-monotonicity in computationally modeled curves shown in Figures SN1I, SN4I-SN6I). Interestingly, the asymptotic tail of the distribution is still somewhat well described by numerical solutions to Equation SN53, provided the constraint $L_2 = L_{boundary}$ is applied such that the boundary condition effectively acts as an artifactual source at $L_{boundary}$ (i.e., there is agreement between numerical and computationally modeled distributions for $\Delta\tau = 0$ in Figures SN1I, SN4I-SN6I, but only at long lengths that have equilibrated to the boundary). At longer timescales, the non-equilibrium behavior may overwhelm this artifactual effect.

5.0.1 Comparisons of approximations to the dynamics and steady-state distributions

Figure SN1 compares computationally modeled distributions for these example parameter combinations to the geometric distribution that describes the short length regime (Equation SN10 using values the average single-unit context rates $\mu = \mu_{B\to A}$ and $\nu = \mu_{A\to B}$) and to the three nested approximations of the long length tail of the distribution obtained by numerically solving Equation SN47, SN48, and SN53. These plots contrast numerical solutions produced under the full set of dynamics (excluding fusion), in the absence of influx due to fission, and in the absence of fission entirely. By observing the lengths at which each successive approximation breaks down, we localized the incoming and outgoing flux due to fission in length space.

For the same parameter combinations, Figure SN2 shows the total influx and outflux for each length associated with the individual effects of expansion, contraction, substitution-based fission, and insertion-

bioRxiv preprint doi: https://doi.org/10.1101/2025.01.09.631797; this version posted January 10, 2025. The copyright holder for this preprint (which was not certified by peer review) is the author/funder, who has granted bioRxiv a license to display the preprint in perpetuity. It is made available under aCC-BY 4.0 International license.

based fission (as well as fusion and and local transitions due to substitutions). The total flux was separately normalized for each bin (for visualization purposes, as the true magnitudes differ dramatically); bins with equal incoming and outgoing net flux have reached equilibrium (i.e., are maintained in a detailed balance). Slight deviation from this equilibrium occurred in every run, indicating a true steady state distribution was not yet reached (as expected in finite time). However, for all results of interest, the magnitude of deviation is very small. Some parameters with modest values of $\Delta\tau$ showed slight deviation from equilibrium at L=1 characteristic of disequilibrium between the A and B distributions (i.e., a source of new A counts at the $L = 1$ boundary) but showed detailed balance across all remaining classes. This is in contrast to unstable parameter combinations, which harbor large deviations from equilibrium in many or most length classes; at the same time, non-equilibrium simulations rapidly populated the largest length classes, subsequently equilibrating to the artificially imposed boundary condition at the large L boundary of the computationally modeled grid.

Figure SN3 provides an alternative characterization of the flux for each bin: the incoming and outgoing fluxes for each mutational effect were plotted separately, rather than computing their net (substitutions were separated into local and nonlocal contributions to demonstrate subdominance of the former relative to expansion and contraction). When separating into directional fluxes, the dominance of expansion and contraction over all other fluxes in both directions is made clear; the much higher rates of repeat instability dominate local fluxes throughout the long length regime. This also leads to large-scale diffusion, which captures the significant bidirectional flux that occurs for both expansions and contractions.

Figure SN4 shows a direct comparison between the computationally modeled sum of all fluxes (including fusion) at each length for comparison to three approximations of the full dynamics: local dynamics alone, local dynamics and fission outflux (treating influx as negligible), and local dynamics along with a full model of fission (all transitions other than fusion). The accuracy of each approximation can be seen at each length bin via the overlap with the net flux iunder the full model. In contrast to Figure SN1, which compares numerical solutions that approximate the steady-state distributions, these plots directly compare components of the finite difference equation (and, in a continuum approximation, the differential equations in steady state) for each model specified by Equations SN37, SN47, and SN48. In particular, the nonlocal interactions in Equation SN37 are accounted for directly, without requiring the intermediate step that leads to Equation SN53. This provides a complementary set of comparisons that lead to the same qualitative observations about the regime of validity and accuracy of each approximation across the parameter space, while retaining length-dependent information about the role of each effect across the long repeat regime. Additionally, this provides further justification for the assumption that repeat fusion remains negligible for long repeat dynamics, despite it's qualitative importance to short repeat dynamics.

Figures SN5, SN6, and SN7 show comparisons between numerical and computational results for the same values of τ_ϵ, τ_κ, and $\Delta\tau$ shown in Figure SN1, but with multipliers of $m = \{2, 16, 32\}$, respectively. For intuition about the effect of the dynamics on the genome size, all comparisons below are shown for non-normalized distributions. The total genome-wide target for A bases, $\bar{L}_A$, corresponds to the weighted mean of the non-normalized distribution: $\bar{L}_\mathrm{A} = \sum_{L=1}^{\infty} L\ P(L) \approx \int_0^\infty dL\ L\ \rho(L)$. The total genome size is the sum of $\bar{L}_A$ and the corresponding target for B bases $\bar{L}_B$. Comparing the same values of $\Delta\tau$ across multipliers m, we found qualitative consistency of the decomposition into three dynamical regimes but with boundaries that quantitatively depend on m. The approximations represented by numerical solutions to Equation SN47 (along with the rough analytic solution) and Equation SN48 break down at a larger values of $\Delta\tau$ for smaller m due to the added contribution of substitutions to the fission rates. For example, in Figure SN5 for $m = 2$, the purely local approximation in Equation SN47 breaks down at or above $\Delta\tau = 2$, rather than at roughly $\Delta\tau \sim 1.5$ for $m = 8$ shown in Figure SN1. Here, the relative rate of substitution is closer to the insertion rate and the total rate of fission is closer to the expansion and contraction rates. The relative increase in the strength of fission implies that fission plays a more substantial role in the maintenance of the steady state distribution. In contrast to the more aggressive approximations, numerical solutions to Equation SN53 (i.e., the continuum model with all effects except fusion) remain accurate across the $\tau_\epsilon + \tau_\kappa = 3$ line, even at low values of m.

Figure SN8 shows comparisons between the computationally modeled distribution, numerical solutions,

bioRxiv preprint doi: https://doi.org/10.1101/2025.01.09.631797; this version posted January 10, 2025. The copyright holder for this preprint (which was not certified by peer review) is the author/funder, who has granted bioRxiv a license to display the preprint in perpetuity. It is made available under aCC-BY 4.0 International license.

and the closed form approximation in Equation SN50 for parameter combination within and outside of the regime of validity of the latter (i.e., only appropriate when $\Delta\tau \gg 1$). Parameter combinations are shown for $\Delta\tau = \{1, 3, 5\}$ and $m = \{2, 8, 32\}$. This rough approximation captures the asymptotic falloff of the distribution when $\Delta\tau$ is sufficiently large (roughly $\Delta\tau \gtrsim 3$), failing to characterize the shape at lengths closest to the lower boundary of the long length regime around $L = 10$. Equation SN50 shows that the dependence on the constants C_ϵ and C_κ in Equation SN47 approximately cancels, yielding an m-independent expression (i.e., the long length tail of the distribution becomes insensitive to the multiplier). Equation SN10 is also independent of m below $L = 9$ because substitution dominates the dynamics. The shape of the distribution is therefore largely independent of m when $\Delta\tau \gg 1$, except at the transition between the short and long length asymptotic behavior, which is localized to a tight range of lengths; this insensitivity to m can be seen for larger $\Delta\tau$ values in Figure SN8. As m qualitatively captures the relative strength of the substitution and repeat instability rates, the dynamics of long length repeats decouples nearly perfectly from the short length regime when $\Delta\tau \gg 1$, effectively only interacting via a local flux across the length regime boundary at $L = 10$.

6 Empirical constraints on parametric model

Using our computational model, we compared distributions at the final time point (i.e., the steady state distribution for stable parameters) to the empirical distribution of A mononucleotide repeats using the metric defined in Equation 1 in **Methods**. Using the bootstrap procedure defined in **Methods**, we found the subset of parameters statistically consistent with the parameter combination that produced the minimum metric value (i.e., the *best fit* parameters). The best fit parameter values at the metric minimum are $\{m, \tau_\epsilon, \tau_\kappa\} = \{2, 1.5, 1.8\}$. Under our parameterization, a flat direction emerged in metric space roughly along lines of constant $\Delta\tau$ (best fit value $\Delta\tau = 0.3$), consistent with our understanding of the parameter space, detailed above. **Figure 2b** shows the metric values across slices of constant m; we believe that this fully explores the range of parameters consistent with all mathematical and biological constraints (e.g., monotonic rate increases for expansion, contraction, and non-motif insertion rates due to repeat instability, maintenance of the linear mutation regime such that transition probabilities at all lengths do not approach one, etc.). Here, we summarize these results interpreted in the context of the analytic model for long repeat dynamics presented in this supplementary note.

6.1 Constraints on $\Delta\tau$ and L^*

The subset of parameter values consistent with the metric minimum are poorly localized in τ_ϵ and τ_κ, but highly localized along a cline in $\Delta\tau$ (henceforth, *minimum cline*). For large multipliers (roughly $m \geq 8$), the minimum cline follows the off-diagonal line of constant $\Delta\tau = 0.4$ but includes a subset of parameters with $\Delta\tau = 0.3$ and 0.5. For smaller multipliers, the slowly evolving regime (i.e., roughly $\tau_\epsilon, \tau_\kappa \lesssim 1$) includes stable values at $\Delta\tau = 0.2$ (and a varying number of slowly evolving parameters with $\Delta\tau < 0.2$ that have not equilibrated in the allotted time or will slowly diverge); however, the steady state distribution in this regime is highly dependent on substitution-based fission, with a corresponding equilibration timescale greater than the inverse of the substitution rate (approximately $10^9 - 10^{10}$ generations), which is likely inconsistent with the rate of evolution of the distribution. Additionally, the cline deviating from constant $\Delta\tau$ includes values that do not equilibrate within the computationally modeled timescale (in excess of 10^9 generations). This suggests that the realistic and sufficiently equilibrated subset of inferred values to spans $\Delta\tau = 0.3 - 0.5$. Using Equation SN23, we find a range of values $L^* \approx 23 - 42$ (inclusion of low m values with $\Delta\tau = 0.2$ gives an upper value of $L^* = 92$). L^* localizes the transition point between expansion-biased (below) and contraction-biased length ranges and represents a dynamical shift from a net length-increasing to net length-decreasing flux due to the combined effects of expansion and contraction. We find that this occurs at intermediate lengths within the long length regime, below the truncation point of the distribution, which we estimate at roughly $L_{\max} = 65$. Notably, within the discrete grid of parameter values explored, values of $\Delta\tau < 0.3$ correspond to $L^* > 90 > L_{\max}$, significantly beyond the empirically observed truncation point of the distribution (i.e., the

bioRxiv preprint doi: https://doi.org/10.1101/2025.01.09.631797; this version posted January 10, 2025. The copyright holder for this preprint (which was not certified by peer review) is the author/funder, who has granted bioRxiv a license to display the preprint in perpetuity. It is made available under aCC-BY 4.0 International license.

value of $L_{\max}$ for the genome-wide distribution of A repeats). Indeed, distributions within the latter regime extend to near or beyond the boundary of the computational grid at $L_{boundary} = 200$; in contrast, we observe few populated length classes above roughly $L_{\max} \sim 65$ in the human genome.

6.2 Constraints on L_{fis}

As our inference showed statistically consistent metric values along a cline of $\Delta\tau$, we were unable to isolate the relative contribution of substitutions and insertions to repeat fission using the inference dataset alone. In other words, L_{fis} (see Equation SN44) could not be estimated from the metric cline, as consistent values of τ_ϵ span the whole parameter space (i.e., corresponding estimates of L_{fis} range from order ten to infinity). However, we analyzed a distinct dataset using popSTR [2] by linearly regressing the expansion and contraction rate power laws to estimate a range of consistent values of $\{m, \tau_\epsilon, \tau_\kappa\}$ as a validation step (see **Methods**; **Figure S6b**). This produced a small subset of parameters along the minimum cline (parameters within boxes in **Figure 2b**) and limited allowed values of the multiplier to roughly $m \sim 8 - 16$ and $\tau_\epsilon \sim 1.6 - 3.1$ (overlapping parameters follows the cline of $\Delta\tau = 0.3$ such that $\tau_\kappa \sim 1.9 - 3.4$). The overlapping region of our inference and popSTR-estimated parameters allowed for estimation of a range of consistent values of $L_{\text{fis}} \approx 12 - 26$.

These estimated values are reasonably well localized, suggesting that fission of repeats shorter than L_{fis} (in a range of roughly $10 - 25$) are primarily a result of substitution-based interruptions. At long lengths $L > L_{\text{fis}}$, fissions largely result from insertions. The inclusion of values closer to $L_{\text{fis}} \sim 10$ suggest the possibility that nearly the entire long length regime evolves independently of substitutions; the upper estimate of $L_{\text{fis}} \sim 25$ still describes substitution-independent asymptotic dynamics for the majority of populated long length classes in the genome. Above L_{fis}, the dynamics are entirely independent of substitution, controlled only by mutational effects categorized as repeat instability. This suggests that, in addition to the separability of long repeat dynamics from the substitution-driven short repeat regime, most long repeats are also *mechanistically independent* from short repeats; the primary mutational mechanisms that alter repeat length may be categorically different from replication and repair pathways that generate substitutions in shorter sequences. In this sense, two distinct boundaries can be placed on the lengths of repetitive sequences, corresponding to up to three distinct regimes: repeats below roughly 10 nucleotides are primarily subject to random substitutions; repeats below L_{fis} (where $25 \gtrsim L_{\text{fis}} \gtrsim 10$), which may experience expansion and contraction, are subject to substitution-based interruptions; repeats above L_{fis} primarily exhibit repeat instability-based length changes, evolving both dynamically and mechanistically independently from shorter-length repeats subject to substitution-based effects.

Supplementary References

1. Wolfram Research, Inc. *Mathematica, Version 14.0,* Champaign, IL, (2024).
2. Kristmundsdottir, S. *et al.* Sequence variants affecting the genome-wide rate of germline microsatellite mutations. en. *Nat Commun* **14,** 3855 (2023).

bioRxiv preprint doi: https://doi.org/10.1101/2025.01.09.631797; this version posted January 10, 2025. The copyright holder for this preprint (which was not certified by peer review) is the author/funder, who has granted bioRxiv a license to display the preprint in perpetuity. It is made available under aCC-BY 4.0 International license.

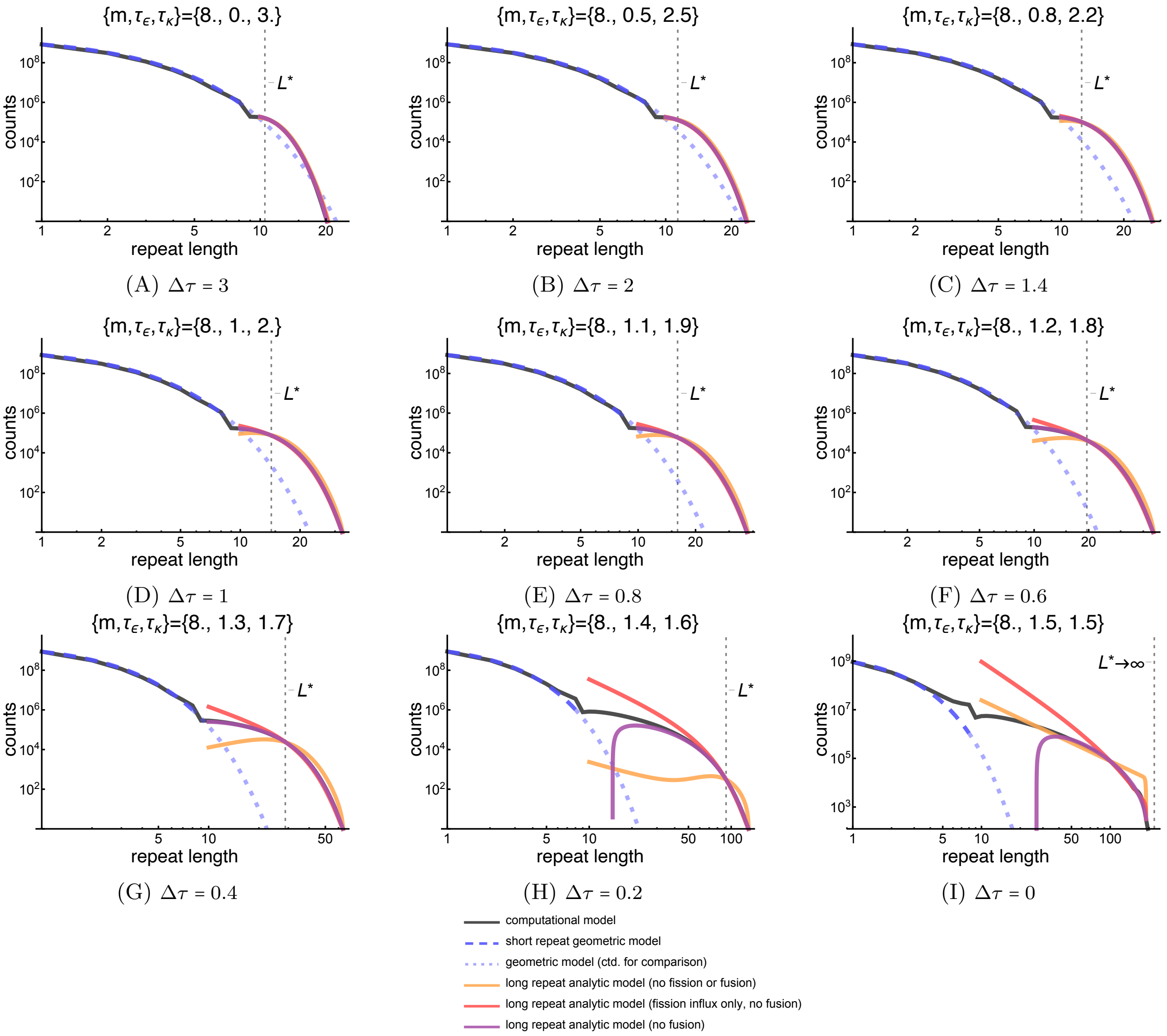

(A) $\Delta\tau = 3$ (B) $\Delta\tau = 2$ (C) $\Delta\tau = 1.4$

(D) $\Delta\tau = 1$ (E) $\Delta\tau = 0.8$ (F) $\Delta\tau = 0.6$

(G) $\Delta\tau = 0.4$ (H) $\Delta\tau = 0.2$ (I) $\Delta\tau = 0$

Figure SN1: *Comparison between computational model results and numerical solutions to steady state equations for $m = 8$ for parameter values with of constant $\tau_\epsilon + \tau_\kappa = 3$.* Each inset shows plots of the computationally modeled distribution at the final time point (black), geometric approximation for shorter repeats of length $L < 10$ (blue, continued as blue dashed line for comparison to distribution tail shape), numerical solutions to Equation SN48 with no fission (orange), numerical solutions to Equation SN47 with fission out but no fission in (red), and numerical solutions to Equation SN53 with fission (purple). (A-C) Comparisons for parameter combinations with $\Delta\tau \gtrsim 1.5$ (referred to as $\Delta\tau \gg 1$) show good agreement for all numerical solutions; fission is negligible. (D) Boundary between large $\Delta\tau$ and intermediate values where fission out first becomes relevant. (E) Fission out is relevant for $\Delta\tau \sim 1$, but only for repeats $L < L^*$ (i.e., deviation of orange line at $L \sim 10 - 13$). (F, G) Effects of fission in are relevant for $L^* > L \gtrsim 10$, while longer lengths are well approximated by considering only fission out and local dynamics; approximation with local dynamics alone remains reasonable for determining the location of L_{max}. For $m = 8$, $\Delta\tau = 0.4$ is the most consistent with the empirical distribution. (H) L^* lies close to the computational grid boundary at $L = 200$. This distribution would stabilize by truncating at a length above $L = 200$ if the grid was extended far beyond realistic lengths. (I) Unstable dynamical regime subject to nonlinear growth. The distribution shows clear interaction with the reflecting boundary.

bioRxiv preprint doi: https://doi.org/10.1101/2025.01.09.631797; this version posted January 10, 2025. The copyright holder for this preprint (which was not certified by peer review) is the author/funder, who has granted bioRxiv a license to display the preprint in perpetuity. It is made available under aCC-BY 4.0 International license.

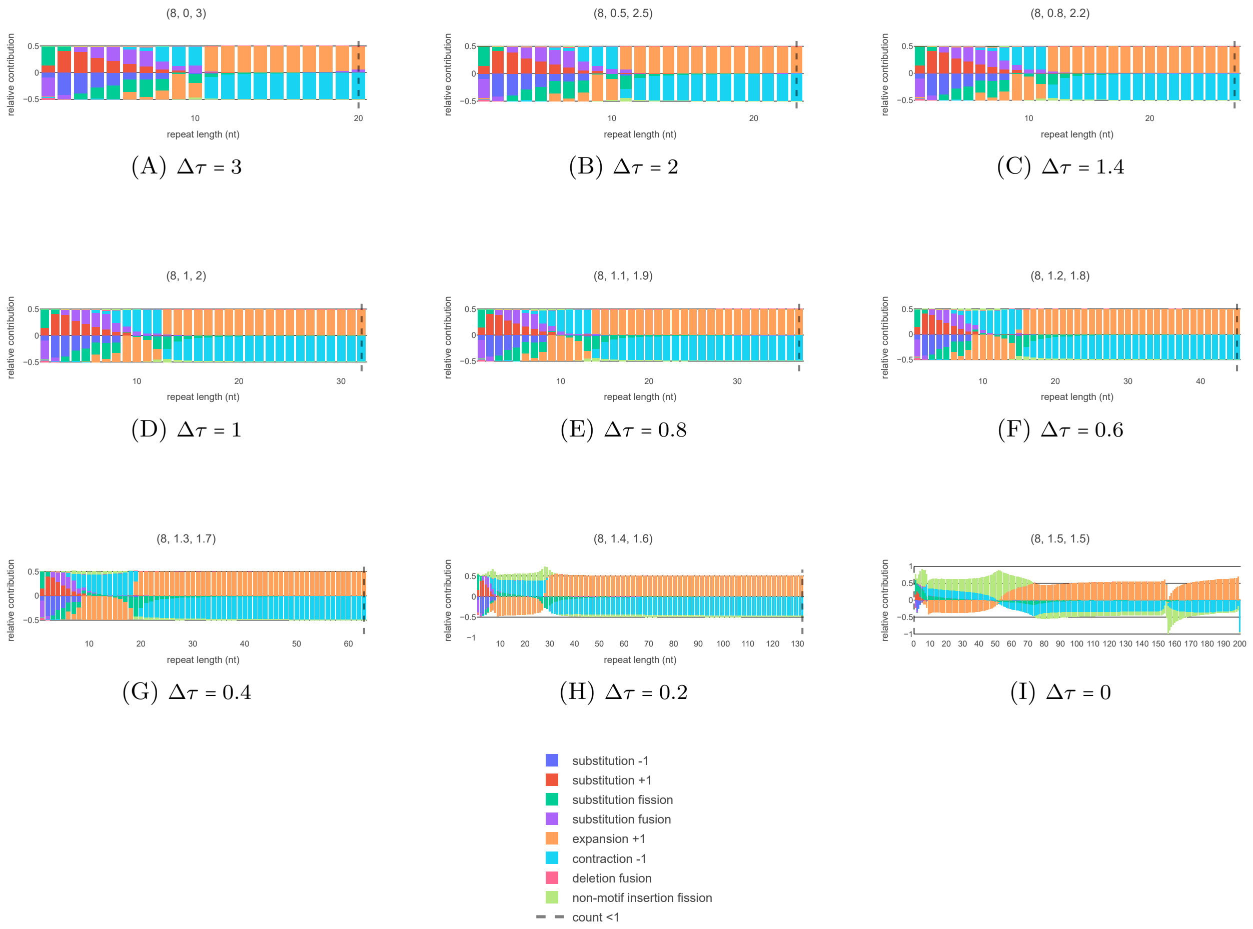

Figure SN2: *Computational model results for the net flux per mutation type.* Subplots (A)-(I) correspond to the same parameters shown in Figures SN1. After the final time point of each run, flux in and out of each bin was calculated and attributed to the following transitions: local length increase due to μ substitutions (darker blue); local length decrease due to ν substitutions (darker red); nonlocal fission generated by ν substitutions (darker green); nonlocal fusion due to μ substitutions (purple); local length increase due to expansion (orange); local length decrease due to contraction (lighter blue); nonlocal fusion resulting from deletion of a B base (lighter red); nonlocal fission due to non-motif insertions (lighter green). Dashed black line shows longest populated length L_{max} where $\rho(L > L_{max}) < 1$. Net flux per category was computed as flux in minus flux out (i.e., net change in the number of repeats per length class per mutational transition). After computing the net flux for each effect, the sum of magnitudes of all effects was separately normalized at each length (i.e., height of stacked bars sums to one). If a given transition results in a net influx (outflux), associated bar appears above (below) the axis. Bins showing identical heights above and below zero are maintained in detailed balance.

bioRxiv preprint doi: https://doi.org/10.1101/2025.01.09.631797; this version posted January 10, 2025. The copyright holder for this preprint (which was not certified by peer review) is the author/funder, who has granted bioRxiv a license to display the preprint in perpetuity. It is made available under aCC-BY 4.0 International license.

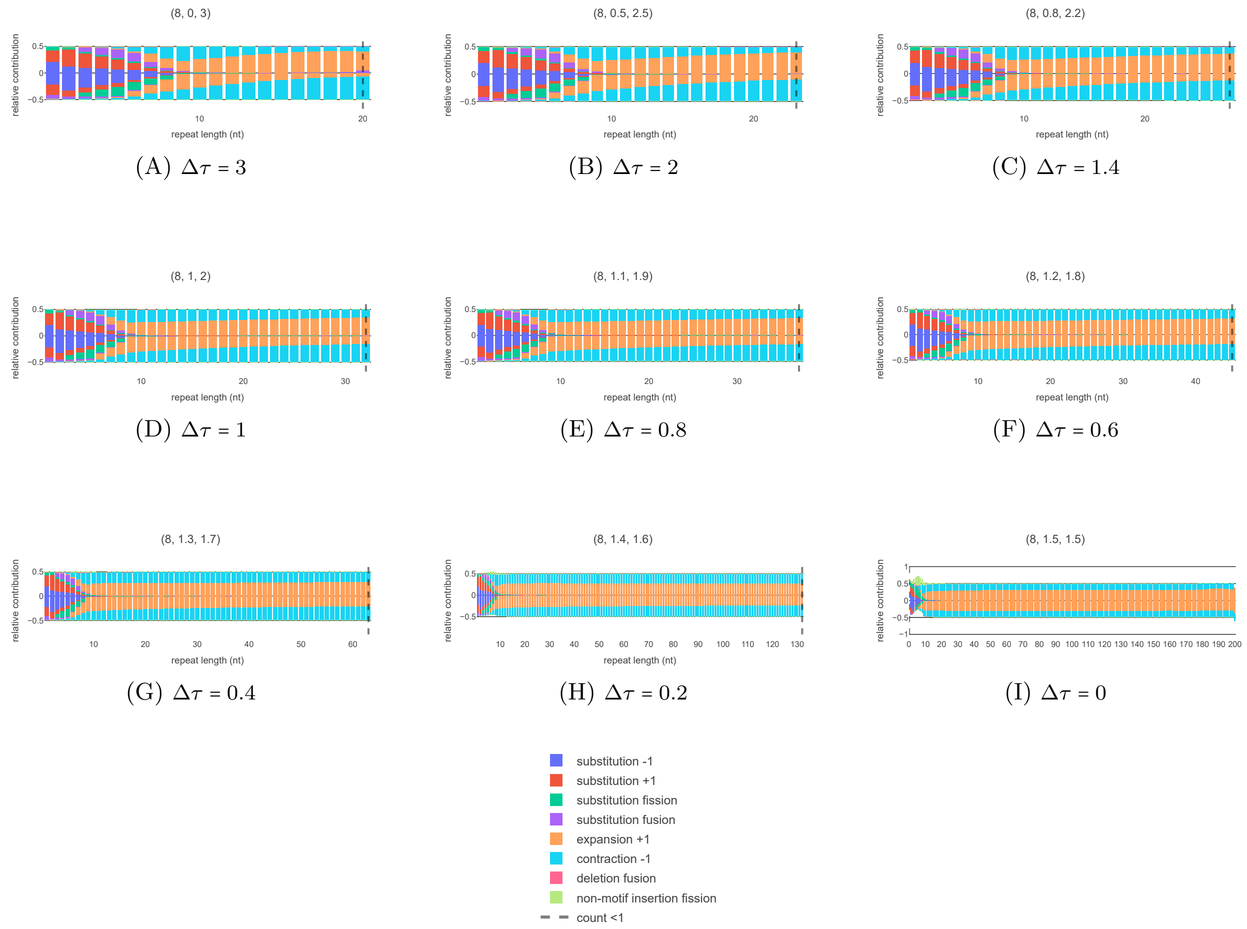

Figure SN3: *Computational model results for directional flux per mutation type* Subplots (A)-(I) correspond to the same parameters shown in Figures SN1. After the final time point of each run, flux in and out of each bin was calculated and attributed the same mutational processes as in Figure SN2 (shown in corresponding colors). For each category, flux in and flux out are plotted separately at each length (shown above and below zero, respectively). The sum of the magnitudes of all effects (influxes plus outfluxes) was separately normalized to one at each length. Bins showing identical heights above and below zero (i.e., influx equal to outflux) are maintained in detailed balance. In contrast to Figure SN2, each bar height represents the fraction of total number of transitions (in either direction) due to each signed mutational transition (e.g., fraction of number of transitions from expansion influx events, expansion outflux events, contraction influx events, etc.).

bioRxiv preprint doi: https://doi.org/10.1101/2025.01.09.631797; this version posted January 10, 2025. The copyright holder for this preprint (which was not certified by peer review) is the author/funder, who has granted bioRxiv a license to display the preprint in perpetuity. It is made available under aCC-BY 4.0 International license.

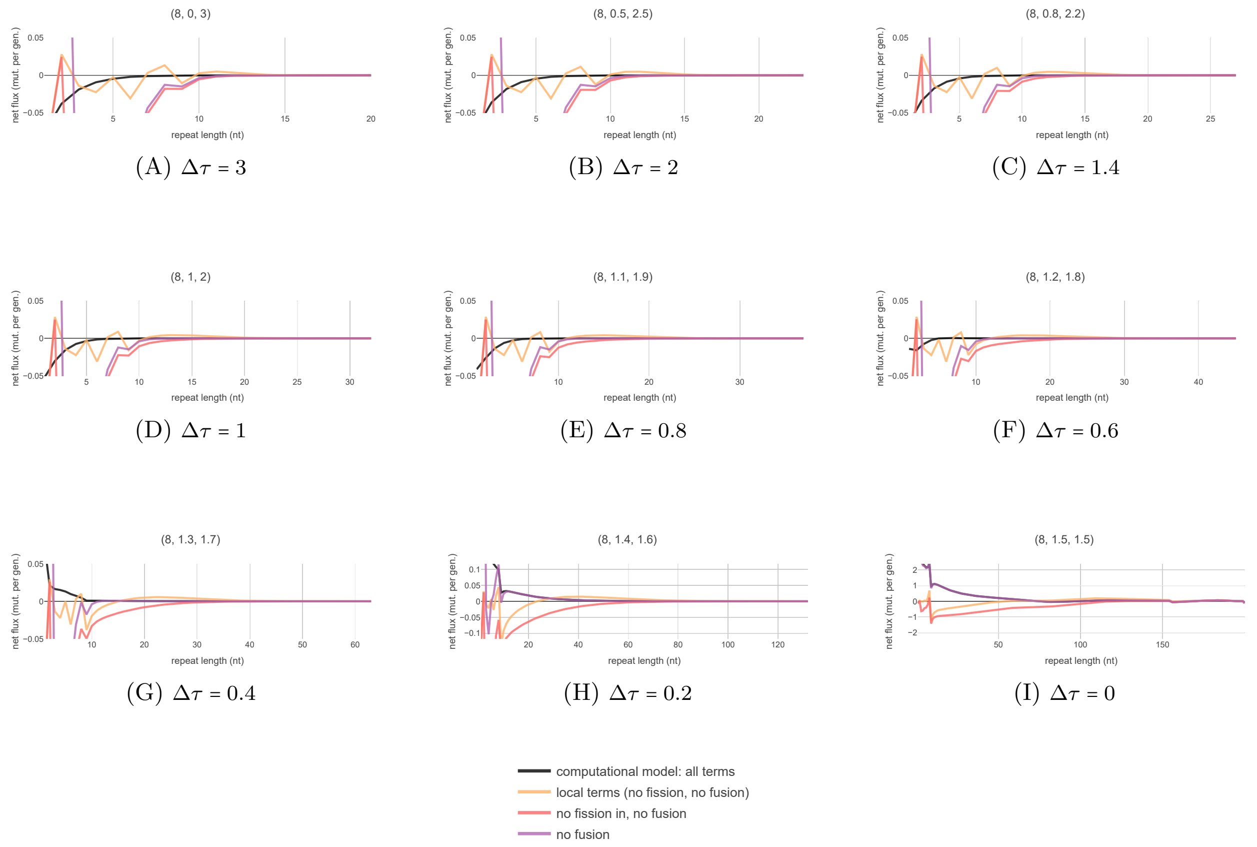

(A) $\Delta\tau = 3$ (B) $\Delta\tau = 2$ (C) $\Delta\tau = 1.4$

(D) $\Delta\tau = 1$ (E) $\Delta\tau = 0.8$ (F) $\Delta\tau = 0.6$

(G) $\Delta\tau = 0.4$ (H) $\Delta\tau = 0.2$ (I) $\Delta\tau = 0$

Figure SN4: *Computational model results for collective non-normalized fluxes showing the relevance of local transitions, fission, and fusion.* Subplots (A)-(I) correspond to the same parameters shown in Figures SN1. At the final iterated time point, fluxes were calculated for each length class and summed appropriately to test the accuracy of different analytic models of the long length regime specified in Equations SN37, SN47, and SN48. Each model specifies an approximate steady state equation assembled as a subset of the full collection of terms shown in Equation SN32. Equation SN32, which includes repeat fusion, was summarized by adding the fluxes due to all mutational effects separately at each length (shown in black); detailed balance occurs when all fluxes sum to zero at a given length. Each approximation is deemed appropriate at lengths where they overlap the black curve (restricted to $L > 10$). In contrast to Figure SN1, which tests the accuracy of solutions to the approximated steady state equations, this comparison tests the differential equation more directly by specifying the magnitude of individual terms in the expression (within a given parameter and length regime); in particular, this comparison captures nonlocal effects in Equation SN37 directly, without reference to Equation SN37. The model missing only fusion (Equation SN37; purple) deviates from the full model (Equation SN32; black) only for $L \lesssim 10$ indicating fusion is negligible in the long repeat regime. All three approximations overlap for large $\Delta\tau$, indicating the dominant behavior is local (described by Equation SN48; yellow); the model with fission treated strictly as an outflux (Equation SN47; red) remains a good approximation to the full effects of fission (purple) above roughly $\Delta\tau \sim 0.6$.

bioRxiv preprint doi: https://doi.org/10.1101/2025.01.09.631797; this version posted January 10, 2025. The copyright holder for this preprint (which was not certified by peer review) is the author/funder, who has granted bioRxiv a license to display the preprint in perpetuity. It is made available under aCC-BY 4.0 International license.

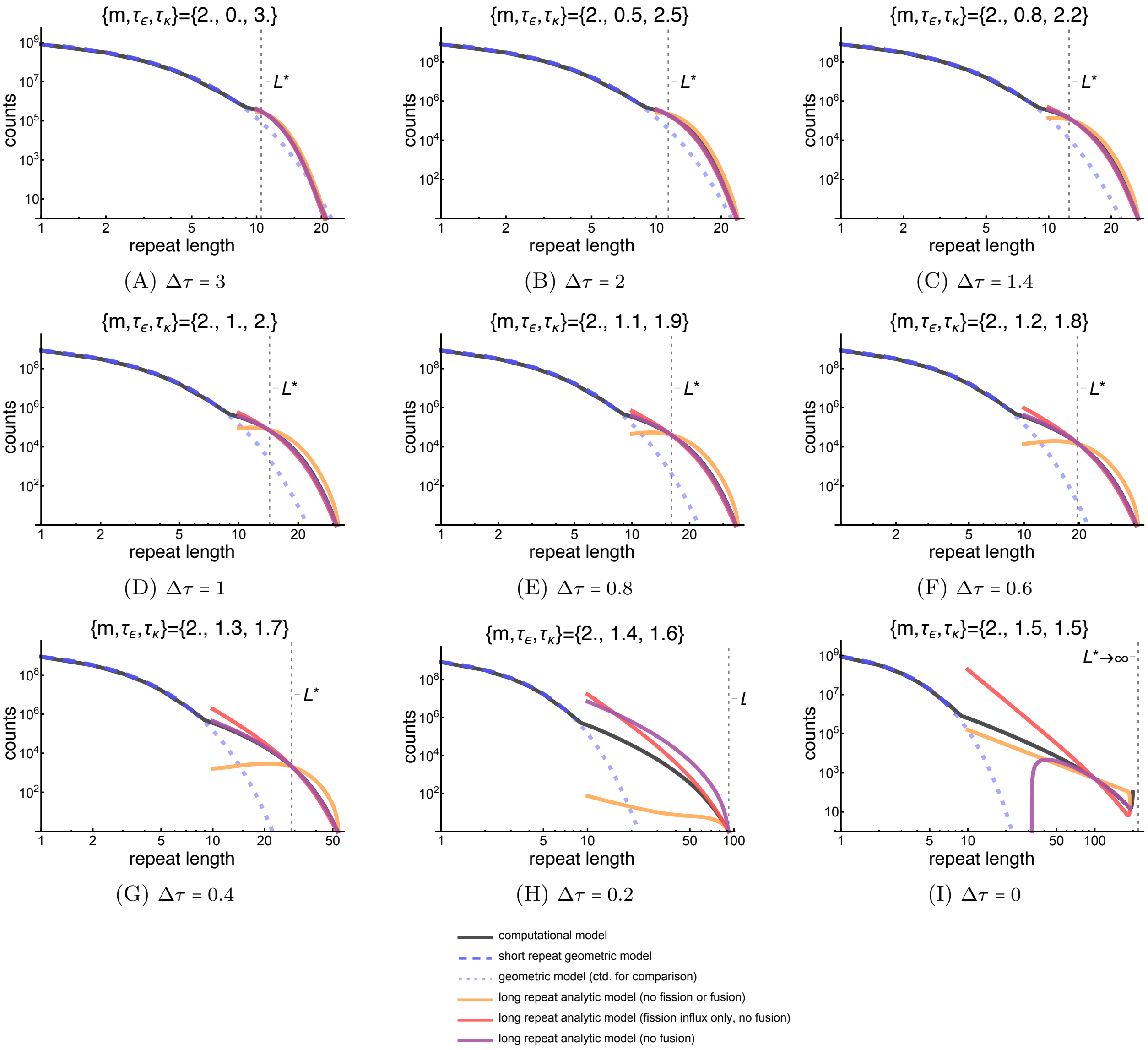

(A) $\Delta\tau = 3$ (B) $\Delta\tau = 2$ (C) $\Delta\tau = 1.4$

(D) $\Delta\tau = 1$ (E) $\Delta\tau = 0.8$ (F) $\Delta\tau = 0.6$

(G) $\Delta\tau = 0.4$ (H) $\Delta\tau = 0.2$ (I) $\Delta\tau = 0$

Figure SN5: *Comparison between computational model results and numerical solutions to steady state equations for* $m = 2$. Computationally modeled distributions are plotted for the same $\{\tau_\epsilon, \tau_\kappa\}$ (and therefore $\Delta\tau$) parameter combinations plotted in Figure SN1 (shown in the same location), but for $m = 2$. Each inset shows plots of the computationally modeled distribution at the final time point (black), geometric analytic approximation for shorter repeats of length $L < 10$ (blue, continued as blue dashed line for comparison to distribution tail shape), numerical solutions to Equation SN48 with no repeat fission (orange), numerical solutions to Equation SN47 with fission out but without fission in (red), and numerical solutions to Equation SN53 with fission out and fission in (purple).

bioRxiv preprint doi: https://doi.org/10.1101/2025.01.09.631797; this version posted January 10, 2025. The copyright holder for this preprint (which was not certified by peer review) is the author/funder, who has granted bioRxiv a license to display the preprint in perpetuity. It is made available under aCC-BY 4.0 International license.

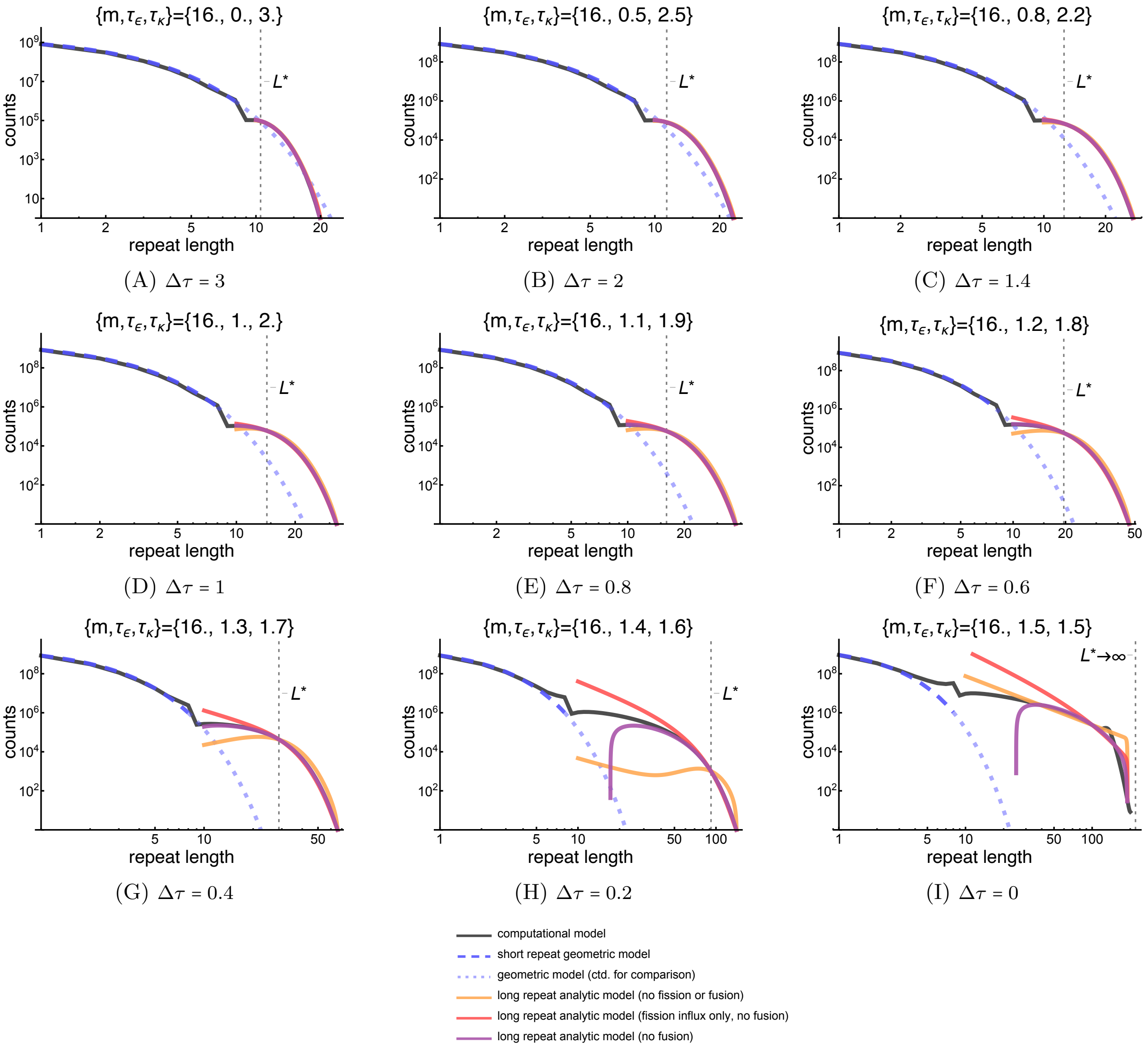

Figure SN6: *Comparison between computational model results and numerical solutions to steady state equations for* $m = 16$. Computationally modeled distributions are plotted for the same $\{\tau_\epsilon, \tau_\kappa\}$ (and therefore $\Delta\tau$) parameter combinations plotted in Figure SN1 (shown in the same location), but for $m = 16$. Each inset shows plots of the computationally modeled distribution at the final time point (black), geometric analytic approximation for shorter repeats of length $L < 10$ (blue, continued as blue dashed line for comparison to distribution tail shape), numerical solutions to Equation SN48 with no repeat fission (orange), numerical solutions to Equation SN47 with fission out but without fission in (red), and numerical solutions to Equation SN53 with fission out and fission in (purple).

bioRxiv preprint doi: https://doi.org/10.1101/2025.01.09.631797; this version posted January 10, 2025. The copyright holder for this preprint (which was not certified by peer review) is the author/funder, who has granted bioRxiv a license to display the preprint in perpetuity. It is made available under aCC-BY 4.0 International license.

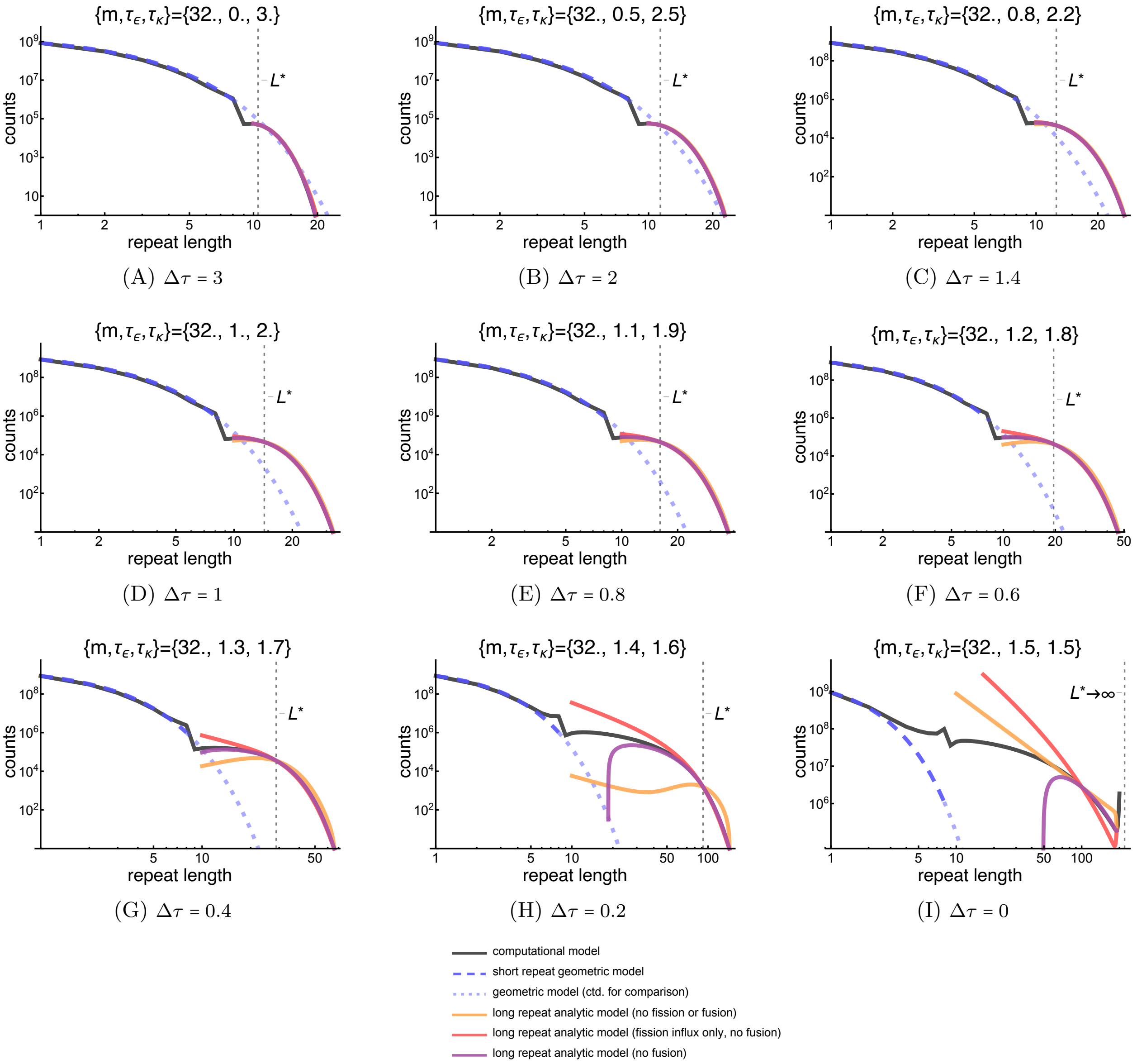

Figure SN7: *Comparison between computational model results and numerical solutions to steady state equations for* $m = 32$. Computationally modeled distributions are plotted for the same $\{\tau_\epsilon, \tau_\kappa\}$ (and therefore $\Delta\tau$) parameter combinations plotted in Figure SN1 (shown in the same location), but for $m = 32$. Each inset shows plots of the computationally modeled distribution at the final time point (black), geometric analytic approximation for shorter repeats of length $L < 10$ (blue, continued as blue dashed line for comparison to distribution tail shape), numerical solutions to Equation SN48 with no repeat fission (orange), numerical solutions to Equation SN47 with fission out but without fission in (red), and numerical solutions to Equation SN53 with fission out and fission in (purple).

bioRxiv preprint doi: https://doi.org/10.1101/2025.01.09.631797; this version posted January 10, 2025. The copyright holder for this preprint (which was not certified by peer review) is the author/funder, who has granted bioRxiv a license to display the preprint in perpetuity. It is made available under aCC-BY 4.0 International license.

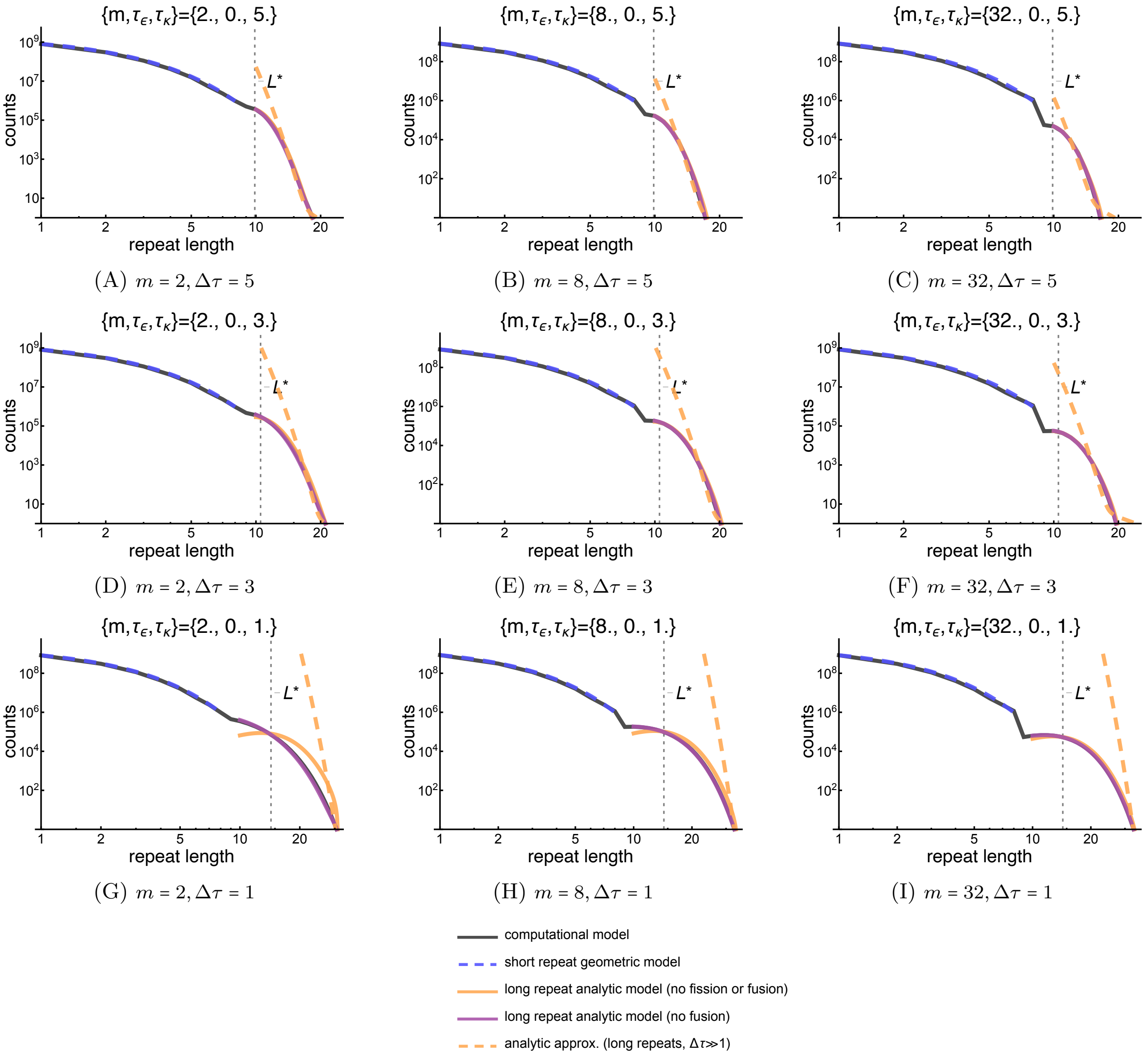

Figure SN8: *Accuracy of analytic approximation for distribution falloff in the $\Delta\tau \gg 1$ regime.* The rough approximation in Equation SN50 to the shape of the steady state distribution when $\Delta\tau \gg 1$ is shown for a wide range of parameter combinations with $\Delta\tau \geq 1$. Each row plots the same combination of $\{\tau_\epsilon, \tau_\kappa\}$ for multipliers $m = 2$ (left), $m = 8$ (middle), and $m = 32$. Each column plots the same value of m for $\Delta\tau = 5$ (top), $\Delta\tau = 3$ (middle), $\Delta\tau = 1$ (bottom). For $\Delta\tau = 5$, the analytic solution approximates the computationally modeled and numerically generated distributions, except at lengths adjacent to the short repeat regime (roughly $12 > L > 10$, noting axes are log spaced). The closed-form solution is independent of m; accuracy and the regime of validity show only a weak dependence on m for $\Delta\tau \gtrsim 3$.
